# Effects of a digital self-control intervention to increase physical activity in middle-aged adults

**DOI:** 10.1177/13591053231166756

**Published:** 2023-04-12

**Authors:** Mirjam Stieger, Mathias Allemand, Margie E Lachman

**Affiliations:** 1 Brandeis University, USA; 2 Lucerne University of Applied Sciences and Arts, Switzerland; 3 University of Zurich, Switzerland

**Keywords:** behavior change, digital intervention, physical activity, self-control

## Abstract

The goal of this study was to test the effects of a 7-week digital self-control intervention to increase physical activity using a two-arm randomized controlled trial. The self-control treatment group showed greater increases in self-reported physical activity (MET’s) than the comparison group. Both groups significantly increased their daily steps and self-control. Participants with higher initial levels of conscientiousness were better able to increase their daily steps during the intervention and participants who increased more in self-control showed greater increases in MET’s. These moderation effects were more pronounced in the self-control treatment group as compared to the comparison group. This study shows that the effects of physical activity interventions may depend on personality characteristics and outcomes may be improved when individual differences are considered and targeted.

An active lifestyle has broad benefits for cognitive, physical, and psychological health (Sanders et al., 2019) and a lack of physical activity is one of the leading causes of preventable death worldwide (Lopez et al., 2006). However, despite the highly publicized benefits of physical activity, 51.1% of middle-aged US adults between 35 and 65 years of age do not achieve the recommended minimum of physical activity needed to maintain health (Center for Disease Control and Prevention, 2020). Middle-aged adults, an often under-studied population, are in a pivotal period in their life course as they may be developing health-promoting behavioral patterns and forming habits that will endure into later life, when physical health problems increase and physical activity tends to decrease (Lachman et al., 2015; Mroczek et al., 2020). Those in midlife are often juggling work and family roles and may have limited time to focus on their own well-being (Lachman, 2004). Factors that contribute to an inactive lifestyle include not only an individual’s health status or the social and environmental context, but also psychological factors such as self-control (Bauman et al., 2012). As such, effective intervention programs that target psychosocial or behavioral factors to increase physical activity in middle-aged adults are urgently needed (Diehl et al., 2020). The present study targets self-control as a behavior change mechanism to promote an active lifestyle.

Self-control refers to the ability to suppress or control impulsive actions, emotions, and desires in favor of desired alternatives and enables people to reach long-term goals (such as sticking to regular exercise) despite the presence of short-term desires and distractions (Duckworth, 2011). If people frequently fail to reach their goals, this is often due to low levels of self-control, a facet of the conscientiousness personality trait. Recent research suggests that facets of conscientiousness such as self-control (Roberts et al., 2014) might be particularly suited to predicting exercise behaviors and goal achievement (Ludwig et al., 2019). High self-control is related to various positive outcomes including physical activity (Bogg and Roberts, 2004). In contrast, low self-control is linked to problematic health behaviors such as a sedentary lifestyle (Wilson and Dishman, 2015). Research suggests that a key to successfully keeping up an active lifestyle in the long run may lie in the ability to be self-controlled, as people with higher self-control are better able to deal with temptations in adaptive ways and form strong habits that last (Gillebaart and Adriaanse, 2017).

The modification of personality facets such as self-control through interventions to promote healthy aging is a research priority of the US National Institute of Aging (Chapman et al., 2014; NIA Workshop, 2016). Indeed, recent smartphone-based interventions showed that it is possible to change personality traits and facets such as conscientiousness and self-discipline with the help of interventions within just a few weeks (Stieger et al., 2020b, 2021b). Other experimental and intervention research also suggests that regular practice of self-control strategies may improve self-control with small-to-medium effects (de Ridder et al., 2020; Friese et al., 2017). The present research constitutes the first to test whether a digital self-control coaching intervention has an effect on physical activity. Digital coaching refers to an intervention, in which a digital coach (e.g. chatbot) is used to deliver the interventional ingredients (Allemand and Flückiger, 2022). Digital interventions are especially promising as they can be delivered independent of time and place and at a relatively high dosage (i.e. multiple times per day).

## The present study

The overall goal of this study was to explore the effects of a digital self-control coaching intervention to increase physical activity among sedentary adults who are at risk for poor health. To measure physical activity, we focused on the richness and complementary information that self-report and objective measurements can offer (Haskell, 2012). Specifically, we used a self-report questionnaire to capture time and intensity spent in specific activities or domains (e.g. work, leisure, transport). Also, a digital device (Fitbit) was used to objectively measure daily steps and daily active minutes and to address the potential social desirability bias introduced by self-report surveys.

The specific aims of the study were threefold. The first aim was to test the effects of a digital self-control intervention to increase physical activity by comparing the two conditions (i.e. treatment group vs comparison group). It was expected that those who received the self-control intervention, would show greater increases in physical activity compared to people in the comparison group, who did not receive the self-control intervention. As part of the first study aim, it was also expected that the effects in the treatment group would last longer compared to the changes in the comparison group. Also, based on prior research, which found that higher conscientiousness was associated with greater increases in daily steps during an intervention (Stieger et al., 2020a), we explored if participants with higher initial levels of conscientiousness were better able to work toward their goal to increase their physical activity over time and if this effect differed between the two conditions.

The second aim was to compare changes in self-control between the treatment group and the comparison group. We expected that participants in the self-control treatment group would show greater increases in self-control compared to participants in the comparison group and that self-control changes in the treatment group would last longer compared to changes in the comparison group.

The third aim was to examine whether participants who increased more in self-control also showed greater increases in their physical activity and if this effect was moderated by their condition. We expected that those who showed greater increases in self-control would also show greater increases in physical activity.

In addition, we explored whether participants changed in the secondary outcome measures (Big Five personality traits, exercise self-efficacy, satisfaction with life, sense of control, and cognitive performance) across time and whether these changes differed between conditions.

## Method

This study has been approved by the University’s Institutional Review Board at Brandeis University and the protocol has been registered at ClinicalTrials.gov (CT04522141) and is published (Stieger et al., 2021a).

### Participants

A total of 86 middle-aged adults were enrolled in the study between October 2020 and April 2021. Participants were recruited from across the US, with a majority in the Northeast, by posting advertisements on public boards and online. Advertisements specified: “Do you want to be more active, but find yourself making excuses to avoid exercise?” Participants received a Fitbit Charge 4 step counter and a $25 gift card for completing all aspects of the study.

Interested participants were asked to complete a screening questionnaire. Participants were included if they indicated they wanted to become more physically active, were between 35 and 65 years, fluent in English, fit enough to walk for at least 20 minutes at a time, and own a smartphone with a data plan. Participants were excluded if they experienced a fall or heart problem/condition in the last 6 months before the start of the study, were already participating in an exercise program or using a Smartwatch (e.g. Fitbit, Garmin, Apple Watch etc.), already exercised (all types of exercise as defined by the person) at least 3 times per week for more than 30 minutes at a time, and/or if a doctor had advised them to not walk due to health conditions.

As shown in Supplemental Table 1, the mean age of participants was 46.96 years (SD = 9.54) and 77.5% were female. Also, 66.3% of all participants identified their race as White. The average years of education completed were 16.51 (SD = 2.87). As shown in the CONSORT flow diagram (Supplemental Figure 1), a total of 81 participants provided data and were included in our intent-to-treat analyses (self-control treatment group: *n* = 42; comparison group: *n* = 39). Sample sizes for each measurement occasion in each condition are reported in Supplemental Table 1.

### Study design and procedure

This study used a two-arm randomized controlled trial design with a pretest (baseline), weekly assessments at the end of every week (weeks 1–7), a posttest (week 8), and a follow-up assessment (week 12). Participants were randomly assigned to one of the two conditions: the treatment group or the comparison group. The allocation was computer generated when participants declared interest for study participation and block randomization was used to ensure that the two groups are comparable in terms of sample size. Participants were blind to which condition they were in. The CONSORT flow diagram of the study is shown in Supplemental Figure 1. After the computer-generated randomization, a research assistant called all eligible participants to receive their oral informed consent by phone, to help them install the application on their own smartphone, and to answer any questions. Participants were randomized before they gave oral informed consent because the instructions on the phone were not identical. Study materials (i.e. Fitbit Charge 4, instruction materials, stylus pen, face mask) were shipped to their home address.

On the first study day, all participants were asked to complete an online pretest on Qualtrics and to wear their Fitbit Charge 4 every day for at least 8 weeks from when they wake up to when they go to bed. During the first study week, participants were only asked to wear the Fitbit to establish their baseline activity. Across the study, participants also completed weekly assessments of self-control. After 8 weeks (1 baseline week and 7 study weeks), participants were asked to complete an online posttest and after 12 weeks an online follow-up assessment.

### Self-control treatment group

The treatment group used the MindHike coaching application, which supports and guides people who would like to train their self-control with the help of a digital coach. The app delivered the same dialogs to all participant in the treatment group. There was no bi-directional communication, but participants clicked on predesignated responses to advance the chatbot dialog. The original MindHike coaching app targets self-control without focusing on the domain of physical activity (Allemand et al., 2020).

Participants were contacted by the app twice each day for 8 weeks (one baseline week and seven intervention weeks). During the baseline week, the first daily message was a push notification which reminded them to wear their Fitbit at customized times in the morning. The second daily push notification reminded participants to sync their step data and charge the Fitbit at customized times in the evening. During the seven intervention weeks, participants received an additional short daily chatbot-based self-control coaching input.

As part of the self-control intervention, participants set their own physical activity goal because physical activity interventions are more effective when individuals can choose their preferred activities (Mailey and Hsu, 2019). Examples of goals were: “*I will walk for 15 minutes in my neighborhood at least 4 days a week before I start work and at least one weekend day in the morning*.” or “*Every time I am on the phone, I will pace around.*” The self-control intervention provided participants with different strategies on how to achieve their personal goal. The intervention was based on the process model of self-control (Duckworth and Gross, 2020; Duckworth et al., 2016) and the goal was to target each stage of this theoretical model (i.e. (1) how to proactively select situations, (2) how to proactively change certain situations, (3) how to shift attention in a given situation, (4) how to change appraisal of a given situation, and (5) how to change behaviors in a given situation). Interventional components included specific behavioral tasks (e.g. implementation intentions, self-reflection tasks, and short film clips for psychoeducational purposes. Every week of the 7-week intervention, participants focused on one of the five stages of the self-control process model, while the first week was an introductory week and the last week served to practice all strategies. After the 8 weeks, participants were free to continue using the app, but they did not receive further chatbot inputs. For more details, see the study protocol of the present study (Stieger et al., 2021a).

### Comparison group

The comparison group used a minimal app version across the eight study weeks, which sent them two daily reminders. At customized times in the morning, they were reminded to wear the Fitbit and at customized times in the evening, they were reminded to sync their step data with the Fitbit app and to charge their Fitbit device. The reminder in the evening was chatbot-based to make sure the comparison group used the app at the same frequency as the treatment group.

### Primary outcome measures

The study included Qualtrics online questionnaires at the pretest, weekly assessments (weeks 1–7), a posttest (week 8), and a follow-up assessment (week 12).

#### Self-control

Self-control was assessed in two ways. First, we assessed overall self-control at pretest, at posttest, and follow-up assessment using the Brief Self-control Scale (BSCS; Tangney et al., 2004). All 13 items were rated on a scale ranging from not at all (1) to very much (5). All items were recoded such that higher scores indicated higher levels in self-control. Cronbach’s alpha was 0.83 at pretest, 0.86 at posttest, and 0.85 at the follow-up assessment.

Second, to measure weekly self-control, we asked participants to indicate to what extent the same 13 items from the BSCS applied to them with respect to *the past week*. Weekly self-control was assessed at the end of weeks 1–7. All items were rated on a scale ranging from not at all (0) to very much (10). All items were recoded such that higher scores indicated higher levels in self-control. Cronbach’s alphas ranged across the measurement occasions between 0.83 and 0.89.

#### Self-reported physical activity (MET’s)

Self-reported physical activity was assessed at pretest, posttest, and follow-up using the short version of the International Physical Activity Questionnaire (IPAQ; Craig et al., 2003), which assesses physical activity in four domains including leisure time, home (e.g. gardening), work and transport. The IPAQ short form asks about vigorous activities, moderate activities, and walking undertaken in the four domains. Sample items for the scale are “During the last 7 days, on how many days did you do vigorous physical activities like heavy lifting, digging, aerobics, or fast bicycling?.” Metabolic Equivalent of Task units (MET-minutes/week) were computed by multiplying the number of active minutes per day by the number of active days per week by the MET coefficient of activity intensity (vigorous activity = 8 MET, moderate activity = 4 MET, walking = 3.3 MET) (Craig et al., 2003).

#### Objectively assessed physical activity

Fitbit Charge 4 activity trackers (www.fitbit.com) were used to objectively assess physical activity. The Fitabase platform (www.fitabase.com) was used to access participant’s activity data. Daily steps and time spent in moderate-to-vigorous physical activity (MVPA) were used as objective physical activity measures.

##### Daily steps

As there were likely times when participants walked without wearing their Fitbit device, we only included days with more than 500 steps as valid days in our analyses. This is in line with prior studies using step data (Bisson et al., 2021; Robinson et al., 2019). Days with less than 500 steps were coded as missing values. This resulted in 300 measurement points (7.02%) which were coded as missing values of the 4272 total measurement points which were provided by participants. The two conditions did not differ significantly in the number of days with less than 500 steps.

##### MVPA

Daily steps and MVPA can be correlated, but MVPA can pick up on activity that is more strenuous than regular walking. Fitbit categorizes active time into four categories: sedentary, lightly active, fairly active, and very active. In this study, MVPA was calculated by adding up fairly active and very active minutes each day (Robinson et al., 2019). Also, to match these values with the step data, we recoded the daily MVPA values to missing values on days on which participants walked fewer than 500 steps and likely did not wear their Fitbit device.

### Secondary outcome measures

#### Conscientiousness

At pretest, posttest, and follow-up assessment, participants completed the 60-item BFI-2 to assess conscientiousness (Soto and John, 2017). All items were rated on a scale ranging from strongly disagree (1) to strongly agree (5). Cronbach’s alphas ranged across the measurement occasions between 0.81 and 0.87. All other secondary outcome measures (Big Five personality traits, exercise self-efficacy, satisfaction with life, sense of control, and cognitive performance) are not the focus of this article and reported in Supplemental Appendix A.

### Covariates

Age, sex (0 = female, 1 = male), education in years, race (0 = White, 1 = Non-White), self-reported functional health (SF-36(Ware and Sherbourne, 1992), and self-reported health status (GHQ-12(Goldberg and Hillier, 1979) were included in our analyses, as these covariates have been shown to be related to the outcomes variables and could also be related to the effectiveness of the intervention.

### Data analysis

Details on the power analyses are reported in Supplemental Appendix A.

We used longitudinal multilevel models and the lme4 package in R (R Core Team, 2022) to investigate the effect of the self-control intervention on all outcome measures. For the analyses, we estimated mixed models with random intercepts and fixed time slopes with two levels. The data structure included repeated assessments of the outcome measures (Level 1: Time) nested within participants (Level 2: Person). Preliminary descriptive analyses have shown that linear models describe the trajectories best. As such, linear time terms were used to be consistent across different analyses and to be able to compare changes in self-control and physical activity between the groups. All models were estimated with maximum likelihood to deal with missing data. For all analyses, we focused on the intent-to-treat sample, which included all participants who started with the study. As an additional robustness check, we ran the analyses with only the participants who completed all three measurement waves. The results were the same across these samples.

To test if participants showed significant changes in our outcome measures, we tested the main effects of time using multilevel models. In additional multilevel models, we tested if there was differential change between the two groups by adding a time by condition interaction term as a Level 2 predictor. Moreover, we tested if both groups were able to maintain their levels of physical activity and self-control until the follow-up assessment 4 weeks after the end of the intervention (main effect of time) and if there were differential maintenance effects between the two groups (time by condition interaction).

To explore if T1 conscientiousness predicted changes in physical activity, we used a time by T1 conscientiousness interaction term as a Level 2 predictor in our multilevel models. To test for any differences between the two conditions, we used a time by T1 conscientiousness by condition interaction term as a Level 2 predictor.

To test if participants who showed greater increases in self-control were better able to increase their physical activity, we added a time by change in self-control (follow-up minus pretest) interaction term as a Level 2 predictor in the multilevel models. Also, we used a time by change in self-control by condition interaction term as a Level 2 predictor to test for any group differences.

## Results

Randomization check and attrition analyses are reported in Appendix A. Of all participants, 42.5% used the app every day out of the 56 possible app usage days. On average, participants of both groups used the app on 47.44 days (SD = 15.19).

### Changes in physical activity

In a first step, we tested if participants showed an overall change in MET’s, daily steps, and MVPA. Supplemental Table 1 shows the descriptive statistics, effect sizes across time and stability coefficients for the primary outcome measures. As shown in the Time effects in Table 1, participants showed a significant overall increase in MET’s from pretest to follow-up (*d* = 0.33) and a significant overall increase in daily steps throughout the intervention (*d* = 0.17). Participants did not show a significant overall change in MVPA.

**Table 1.** Changes from pretest to posttest and follow-up in primary outcomes.

Fixed effects	Self-control		Self-control (Weekly)		MET’s		Daily Steps^a^		MVPA^a^	
	Without cov.	With cov.	Without cov.	With cov.	Without cov.	With cov.	Without cov.	With cov.	Without cov.	With cov.
*Intercept*										
Estimate (SE)	3.25*** (0.09)	3.15** (1.02)	6.05*** (0.16)	7.33** (2.31)	1236.4 (639.7)	5025.20 (5607.62)	6539.84*** (418.61)	−343.66 (4757.06)	25.9*** (4.65)	28.22 (42.47)
95% CI	3.07; 3.42	1.13; 5.17	5.72; 6.37	2.75; 11.91	−24.97; 2498.18	−6103.21; 16,132.55	5708.78; 7369.19	−9789.00; 9112.93	16.64; 35.07	−56.12; 112.62
*Time*										
Estimate (SE)	0.13*** (0.03)	0.13*** (0.03)	0.08*** (0.01)	0.09*** (0.01)	774.0** (262.9)	813.45** (267.64)	9.19** (3.32)	6.69* (3.23)	0.07 (0.04)	0.06 (0.04)
95% CI	0.08; 0.19	0.07; 0.18	0.05; 0.11	0.06; 0.12	252.29; 1291.92	283.58; 1340.89	2.68; 15.69	0.36; 13.02	−0.02; 0.16	−0.03; 0.14
*Condition*										
Estimate (SE)	−0.15 (0.18)	0.01 (0.19)	−0.34 (0.33)	−0.08 (0.34)	-1863.1 (1268.7)	–1986.25 (1320.89)	–125.61 (837.00)	433.18 (747.75)	−6.51 (9.23)	−1.22 (6.90)
95% CI	−0.50; 0.20	−0.35; 0.38	−0.99; 0.31	−0.76; 0.59	−4361.67; 635.43	−4588.03; 615.67	−1787.03; 1532.85	−1050.97; 1916.11	−24.80; 11.77	−14.90; 12.45
*Time by condition*										
Estimate (SE)	0.04 (0.06)	0.05 (0.06)	0.03 (0.03)	0.04 (0.03)	1039.5* (517.8)	1178.59* (527.08)	−5.47 (6.64)	−0.48 (6.46)	−0.14 (0.09)	−0.12 (0.09)
95% CI	−0.07; 0.15	−0.07; 0.16	−0.03; 0.09	−0.02; 0.10	16.00; 2060.72	138.76; 2219.89	−18.48; 7.54	−13.14; 12.18	−0.31; 0.03	−0.29; 0.05

Intent-to-treat sample; Covariates: age, sex, education, race, self-reported functional health, and self-reported health status. Condition: 1 = Treatment group, 0 = Comparison group.

a Until the end of the intervention (Day 56).

* *p* < 0.05. **p < 0.01. ***p < 0.001.

In a second step, we tested if the two groups differed in terms of changes in MET’s, daily steps, and MVPA. As shown in the Time by Condition effects in Table 1, the treatment group (*d* = 0.68) showed significantly greater increases in MET’s from pretest to follow-up as compared to the comparison group (*d* = 0.09) (Supplemental Figure 2). The lower stability coefficients in MET’s in the treatment group (*r* = 0.07) compared to the comparison group (*r* = 0.64) reflect group differences in change. The two groups did not differ significantly in terms of changes in daily steps or MVPA during the intervention. To put the changes in daily steps in context, we also report the percentage of participants who showed a meaningful clinically important difference (MCID) in steps as defined by a 1000 steps higher count (Ramsey et al., 2021). Of all participants, 31.1% increased at least 1000 steps to the posttest and 30.4% to the follow-up assessment. Specifically, in the treatment group, 38.9% increased at least 1000 steps to the posttest, 35.3% to the follow-up. In the comparison group, 23.7% increased at least 1000 steps to the posttest and 25.7% to the follow-up.

In a third step, we tested if changes in physical activity were maintained for 4 weeks after the end of the intervention (between posttest and follow-up). The results suggest that the two groups were able to maintain their levels of MET’s (*B* = 319.37, 95% CI [−554.42;1183.84], SE = 436.28, *p* = 0.467, *d* = 0.05), daily steps (*B* = -18.21, 95% CI [−43.04;6.60], SE = 12.65, *p* = 0.150, *d* = −0.13), and MVPA (*B* = 0.07, 95% CI [−0.18;0.32], SE = 0.13, *p* = 0.597, *d* = −0.37) until at least 4 weeks after the end of the intervention. There were no significant differences between the two conditions.

**Figure 1.**
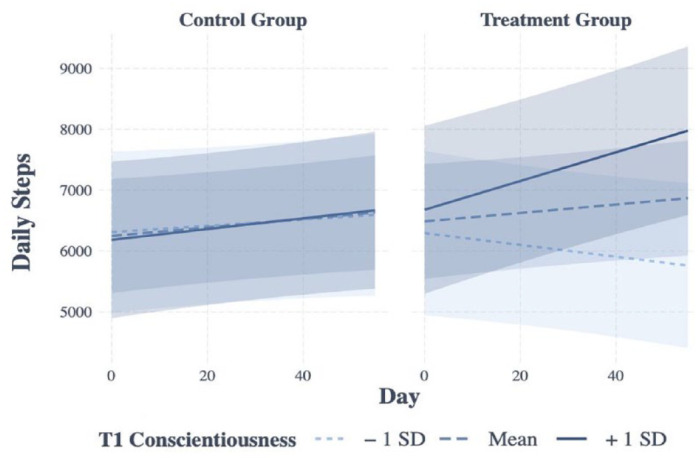
Associations between T1 conscientiousness and daily steps by condition. X-axis: Day 0 until Day 56; Shaded areas represent 95% CIs.

We also explored if participants with higher initial levels of conscientiousness were better able to work toward their goal to increase their physical activity over time. Conscientiousness at T1 was not significantly associated with changes in MET’s or MVPA. However, participants with higher initial levels of conscientiousness were better able to increase their daily steps during the intervention (*B* = 11.21, 95% CI [3.13;19.29], SE = 4.12, *p* = 0.007). Also, there was a significant three-way interaction (Time × Condition × T1 Conscientiousness). That is, participants of the treatment group with high T1 conscientiousness showed greater increases in their daily steps compared to participants of the comparison group with high T1 conscientiousness (*B* = 19.23, 95% CI [3.03;35.44], SE = 8.27, *p* = 0.020) (Figure 1).

### Changes in self-control

The Time main effects suggest a significant overall increase in self-control from pretest to posttest to follow-up (*d* = 0.52) as well as a significant overall increase in weekly measured self-control (Table 1). However, the Time by Condition effects show that the two groups did not differ significantly in terms of their changes in self-control over time (Table 1). Both groups were able to maintain their levels of self-control between the posttest and the follow-up assessment (*B* = 0.08, 95% CI [−0.01;0.16], SE = 0.04, *p* = 0.072, *d* = 0.14). Also, the Time by Condition effects show that the two groups did not differ significantly in the extent to which they were able to maintain their levels of self-control between the posttest and the follow-up.

Supplemental Tables 3–7 show the descriptive statistics, effect sizes across time, stability coefficients, and changes over time in all secondary outcome measures.^1^

### Association between changes in self-control and changes in physical activity

**Figure 2.**
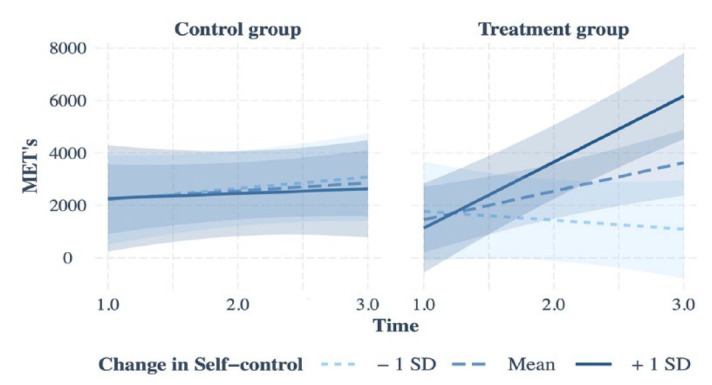
Associations between changes in self-control and changes in MET’s from pretest to follow-up by condition. X-axis: 1 = pretest, 2 = posttest, 3 = follow-up; Shaded areas represent 95% CIs.

We tested a Time by Change in Self-control interaction to examine if participants who showed greater increases in self-control were better able to increase their physical activity across time. The results suggest that participants who showed greater increases in self-control showed greater increases in MET’s (*B* = 1382.5, 95% CI [361.71; 2400.00], SE = 515.9, *p* = 0.009) and daily steps (*B* = 24.15, 95% CI [11.53; 33.76], SE = 6.44, *p* < 0.000), but they did not show greater increases in MVPA. We also explored a three-way interaction (Time × Condition × Change in Self-control) to examine if the effects differed between the two conditions. In terms of MET’s, the findings show that the effect was more pronounced among participants in the treatment group as compared to participants in the comparison group (*B* = 3097.27, 95% CI [1120.90; 5072.99], SE = 1000.64, *p* = 0.002). As such, changes in self-control moderated the relationship between condition and change in MET’s (Figure 2). Changes in self-control did not moderate the relationship between condition and changes in daily steps or MVPA.

The results of all multilevel analyses with covariates mirrored the findings of the models without covariates.

## Discussion

Three important findings emerged from the present study. First, the results suggest significant overall increases in physical activity and self-control in both conditions. Also, both groups maintained their levels of physical activity and self-control until 4 weeks after the end of the intervention. Second, participants of the treatment group showed greater increases in MET’s as compared to participants in the comparison group. Third, participants with higher initial levels of conscientiousness were better able to increase their daily steps during the intervention and participants who increased more in self-control showed greater increases in MET’s. These effects were more pronounced in the treatment group as compared to the comparison group. However, it should be noted that these three-way interactions involve relatively small numbers and should be interpreted with caution.

We found significant group differences in terms of changes in MET’s. However, changes in self-control, daily steps, and MVPA did not differ between the two conditions. The increases of the comparison group suggest that daily reminders and activity feedback on the Fitbit device alone are helpful micro interventions to support people on their journey of becoming more self-controlled and more active. In fact, a recent meta-analysis (Brickwood et al., 2019) found that uses of wearable activity trackers alone can increase physical activity. Also, a recent study found that goal reminders can be effective in promoting self-control and that more strategies simultaneously led to greater self-regulatory success (Milyavskaya et al., 2021). The Fitbit device and the daily reminders might have helped participants of the comparison group to focus on their goal of becoming more active which in turn might have led to increases in self-control. Future additional control groups could help to further tease out the mechanisms involved in behavior change.

The results differed somewhat depending on the activity outcome which was used (i.e. MET’s, daily steps or MVPA). That is, the treatment group showed greater increases in MET’s, but not in daily steps or MVPA, when compared to the comparison group. It is important to note that participants could choose what type of exercise they wanted to implement in their daily life and could set their own activity goal. The IPAQ was used to measure MET’s, which is a valid measure, has acceptable measurement properties, and shows good test-retest stability (Craig et al., 2003). The IPAQ asks individuals to report their active time at work, at home, travel from place to place, and any other activity they do solely for recreation, sport, exercise, or leisure. As such, activity measured with the IPAQ may pick up on more and diverse types of daily activities other than walking/steps. The discrepancy between self-report and activity measures with a tracker has also been found in prior studies (e.g. Garriguet and Colley, 2014; McLean et al., 2020) and is not surprising given that these different approaches measure different aspects of activity.

The results also suggest that those of the treatment group with higher initial levels in conscientiousness or greater increases in self-control were better able to increase their physical activity as compared to those of the comparison group. Previous research also found that participants with higher conscientiousness were better able to increase their levels of physical activity when taking part in a physical activity intervention (Stieger et al., 2020a). Future research is needed to better understand how to tailor interventions toward people who generally benefit less from such interventions such as individuals with low conscientiousness.

### Limitations and future directions

Based on the present findings, it is not entirely clear which micro interventions are most effective in changing self-control and physical activity. Future research is needed to disentangle the specific active ingredients of the intervention. The results on increasing self-control and physical activity in the comparison group, which received only daily app-based reminders and feedback on the Fitbit device, suggest the effectiveness of these micro interventions for physical activity promotion. Second, although the allocation into the two conditions was computer generated to ensure that the conditions were fully randomized with respect to participants’ baseline characteristics, the two conditions differed in some of the baseline characteristics. In our analyses, we controlled for these characteristics to statistically adjust those group differences. Third, the 1-week baseline before the beginning of the intervention might have been too short to track participants’ usual activity behavior. During the first week, participants might be highly motivated to be more active than they were previously just because of the new Fitbit device. In the future, a longer timespan to track usual pre-intervention activity behavior may be worthwhile before testing the effects of the intervention. Fourth, the sample size of this study is relatively small which limits generalizability of our findings. Future research is needed to replicate the findings in larger samples that would allow for further analyses (e.g. to identify for which participants the intervention is most effective). Finally, the 4-week follow-up period after the end of the intervention was relatively short. Future research is needed to replicate the current findings as well as to include longer follow-up periods to test the ideal timing of such an intervention.

## Conclusion

This study constitutes the first to test whether a digital self-control coaching intervention has an effect on physical activity. The results suggest that a digital intervention targeting self-control can help people increase their MET levels for physical activity. Also, individuals with higher levels of conscientiousness and those who show greater increases in self-control are more likely to become more physically active. This intervention study highlights the notion that the effects of behavior change interventions may depend on personality characteristics and outcomes may be improved when individual differences are considered and targeted.

## Supplemental Material

sj-docx-9-hpq-10.1177_13591053231166756 – Supplemental material for Effects of a digital self-control intervention to increase physical activity in middle-aged adults Click here for additional data file. Supplemental material, sj-docx-9-hpq-10.1177_13591053231166756 for Effects of a digital self-control intervention to increase physical activity in middle-aged adults by Mirjam Stieger, Mathias Allemand and Margie E Lachman in Journal of Health Psychology

sj-pdf-1-hpq-10.1177_13591053231166756 – Supplemental material for Effects of a digital self-control intervention to increase physical activity in middle-aged adults Click here for additional data file. sj-pdf-1-hpq-10.1177_13591053231166756 for Effects of a digital self-control intervention to increase physical activity in middle-aged adults by Mirjam Stieger, Mathias Allemand and Margie E Lachman in Journal of Health Psychology This article is distributed under the terms of the Creative Commons Attribution 4.0 License ( http://www.creativecommons.org/licenses/by/4.0/) which permits any use, reproduction and distribution of the work without further permission provided the original work is attributed as specified on the SAGE and Open Access pages ( https://us.sagepub.com/en-us/nam/open-access-at-sage).

sj-pdf-2-hpq-10.1177_13591053231166756 – Supplemental material for Effects of a digital self-control intervention to increase physical activity in middle-aged adults Click here for additional data file. sj-pdf-2-hpq-10.1177_13591053231166756 for Effects of a digital self-control intervention to increase physical activity in middle-aged adults by Mirjam Stieger, Mathias Allemand and Margie E Lachman in Journal of Health Psychology This article is distributed under the terms of the Creative Commons Attribution 4.0 License ( http://www.creativecommons.org/licenses/by/4.0/) which permits any use, reproduction and distribution of the work without further permission provided the original work is attributed as specified on the SAGE and Open Access pages ( https://us.sagepub.com/en-us/nam/open-access-at-sage).

sj-pdf-3-hpq-10.1177_13591053231166756 – Supplemental material for Effects of a digital self-control intervention to increase physical activity in middle-aged adults Click here for additional data file. sj-pdf-3-hpq-10.1177_13591053231166756 for Effects of a digital self-control intervention to increase physical activity in middle-aged adults by Mirjam Stieger, Mathias Allemand and Margie E Lachman in Journal of Health Psychology This article is distributed under the terms of the Creative Commons Attribution 4.0 License ( http://www.creativecommons.org/licenses/by/4.0/) which permits any use, reproduction and distribution of the work without further permission provided the original work is attributed as specified on the SAGE and Open Access pages ( https://us.sagepub.com/en-us/nam/open-access-at-sage).

sj-pdf-4-hpq-10.1177_13591053231166756 – Supplemental material for Effects of a digital self-control intervention to increase physical activity in middle-aged adults Click here for additional data file. sj-pdf-4-hpq-10.1177_13591053231166756 for Effects of a digital self-control intervention to increase physical activity in middle-aged adults by Mirjam Stieger, Mathias Allemand and Margie E Lachman in Journal of Health Psychology This article is distributed under the terms of the Creative Commons Attribution 4.0 License ( http://www.creativecommons.org/licenses/by/4.0/) which permits any use, reproduction and distribution of the work without further permission provided the original work is attributed as specified on the SAGE and Open Access pages ( https://us.sagepub.com/en-us/nam/open-access-at-sage).

sj-sav-5-hpq-10.1177_13591053231166756 – Supplemental material for Effects of a digital self-control intervention to increase physical activity in middle-aged adults Click here for additional data file. sj-sav-5-hpq-10.1177_13591053231166756 for Effects of a digital self-control intervention to increase physical activity in middle-aged adults by Mirjam Stieger, Mathias Allemand and Margie E Lachman in Journal of Health Psychology This article is distributed under the terms of the Creative Commons Attribution 4.0 License ( http://www.creativecommons.org/licenses/by/4.0/) which permits any use, reproduction and distribution of the work without further permission provided the original work is attributed as specified on the SAGE and Open Access pages ( https://us.sagepub.com/en-us/nam/open-access-at-sage).

sj-sav-6-hpq-10.1177_13591053231166756 – Supplemental material for Effects of a digital self-control intervention to increase physical activity in middle-aged adults Click here for additional data file. sj-sav-6-hpq-10.1177_13591053231166756 for Effects of a digital self-control intervention to increase physical activity in middle-aged adults by Mirjam Stieger, Mathias Allemand and Margie E Lachman in Journal of Health Psychology This article is distributed under the terms of the Creative Commons Attribution 4.0 License ( http://www.creativecommons.org/licenses/by/4.0/) which permits any use, reproduction and distribution of the work without further permission provided the original work is attributed as specified on the SAGE and Open Access pages ( https://us.sagepub.com/en-us/nam/open-access-at-sage).

sj-sav-7-hpq-10.1177_13591053231166756 – Supplemental material for Effects of a digital self-control intervention to increase physical activity in middle-aged adults Click here for additional data file. j-sav-7-hpq-10.1177_13591053231166756 for Effects of a digital self-control intervention to increase physical activity in middle-aged adults by Mirjam Stieger, Mathias Allemand and Margie E Lachman in Journal of Health Psychology This article is distributed under the terms of the Creative Commons Attribution 4.0 License ( http://www.creativecommons.org/licenses/by/4.0/) which permits any use, reproduction and distribution of the work without further permission provided the original work is attributed as specified on the SAGE and Open Access pages ( https://us.sagepub.com/en-us/nam/open-access-at-sage).

sj-sps-8-hpq-10.1177_13591053231166756 – Supplemental material for Effects of a digital self-control intervention to increase physical activity in middle-aged adults Click here for additional data file. sj-sps-8-hpq-10.1177_13591053231166756 for Effects of a digital self-control intervention to increase physical activity in middle-aged adults by Mirjam Stieger, Mathias Allemand and Margie E Lachman in Journal of Health Psychology This article is distributed under the terms of the Creative Commons Attribution 4.0 License ( http://www.creativecommons.org/licenses/by/4.0/) which permits any use, reproduction and distribution of the work without further permission provided the original work is attributed as specified on the SAGE and Open Access pages ( https://us.sagepub.com/en-us/nam/open-access-at-sage).

## Notes

1. Due to word limitations, we focus on the primary outcome variables in this study. We report the secondary outcome measures in the Supplemental Materials.
