# Supplementary material for: Effects of a digital self-control intervention to increase physical activity in middle-aged adults
Source: J Health Psychol. 2023 Apr 12;28(10):984–96. doi: 10.1177/13591053231166756 (PMC10466994; doi:10.1177/13591053231166756)
Supplement: sj-docx-9-hpq-10.1177_13591053231166756 – Supplemental material for Effects of a digital self-control intervention to increase physical activity in middle-aged adults [file sj-docx-9-hpq-10.1177_13591053231166756.docx]

**Supplementary Materials**

**Appendix A**

**Method**

**Power Analysis**

We conducted a power analysis for repeated measures, between factors based on the between-group effect size found in a previous Stage 1 study using implementation intentions to promote physical activity, which also included a comparison group with a Fitbit (Robinson et al., 2019). The power analysis with an α level of 0.05, a statistical power (1-β) of 0.80, a correlation of 0.50 between the pretest, posttest, and follow-up assessment, and a Cohen’s *f* = .30 suggests a sample size of at least *N* = 62 with at least 31 participants per condition. As we learned from similar digital physical activity intervention studies (Bisson et al., 2021; Robinson et al., 2019), about 10% of participants who sign up for the study discontinue their participation. As such, we had the goal to recruit at least 70 middle-aged adults (35 participants per condition).

**Secondary Outcome Measures**

Secondary outcome measures were assessed to explore potential transfer effects. Self-control is a sub-facet of conscientiousness. As such, we thought it was reasonable to expect changes in conscientiousness. A recent study found that a self-discipline intervention (also a facet of conscientiousness) led to changes in conscientiousness (Stieger et al., 2020). Also, we expected that positive change experiences may have effects on life satisfaction and other variables as well as potential transfer effects. Additional outcome measures include the Big Five personality traits, exercise self-efficacy, satisfaction with life, and sense of control. Moreover, there are a number of studies that show that even basic walking can have positive effects on cognitive functioning (Kramer & Erickson, 2007; Erickson et al., 2015). Thus, we also included measures of cognitive performance to see if they show changes over time.

**Big Five personality traits.** At pretest, posttest, and follow-up assessment, participants completed the 60-item BFI-2 (Soto & John, 2017). All items were rated on a scale ranging from strongly disagree (1) to strongly agree (5). Cronbach’s alphas ranged across the measurement occasions between .81 and .87 for conscientiousness, between .86 and .87 for openness, between .85 and .87 for extraversion, between .89 and .91 for neuroticism, and between .77 and .85 for agreeableness.

**Exercise self-efficacy.** Exercise self-efficacy was assessed at pretest, posttest, and follow-up using a modified self-efficacy scale ([Neupert, Lachman, & Whitbourne, 2009](https://www.ncbi.nlm.nih.gov/pmc/articles/PMC6440859/#R40)). Participants indicated on a scale of 1 (very sure) to 4 (not sure at all) across nine items how sure they were that they would perform exercise under different conditions or constraints, such as when they were tired and when they were feeling under pressure to get things done. Scores were reverse coded and averaged across items so that a higher number indicated greater self-efficacy. Cronbach’s alphas ranged across the measurement occasions between .92 and .95.

**Satisfaction with life.** Satisfaction with life was assessed at pretest, posttest, and follow-up using the Satisfaction with Life Scale (SWLS; Diener et al., 1985). All items were rated on a scale ranging from strongly disagree (1) to strongly agree (7). Cronbach’s alphas ranged across the measurement occasions between .87 and .92.

**Sense of control.** Perceived control was assessed with the MIDUS sense of control scale (Lachman & Weaver, 1998) at pretest, posttest, and follow-up assessment. The scale consisted of two subscales: personal mastery (e.g., I can do just about anything I really set my mind to) and perceived constraints (e.g., What happens in my life is often beyond my control). All items were rated on a scale ranging from strongly agree (1) to strongly disagree (7). All items were coded so that a higher score reflected greater personal mastery and greater perceived constraints, respectively. Cronbach’s alphas ranged across the measurement occasions between .66 and .84 for personal mastery and between .84 and .89 for perceived constraints.

**Cognitive performance.** Cognitive performance was assessed with three cognitive tests from the Brief Test of Adult Cognition by Telephone (BTACT; Lachman et al., 2014) presented using Qualtrics at pretest, posttest, and follow-up assessment. First, to measure episodic memory, we used the Immediate Word List Recall Task which measures free recall of 15 words. Second, to measure attention switching and inhibitory control, we used the newly developed online app version of the Stop & Go Switch Task (SGST; Liu et al., 2022; Tun & Lachman, 2008). The SGST requires alternating between the “normal” condition (i.e., respond “Go” to the stimulus “Green” and “Stop” to the stimulus “Red”) and the “reverse” condition (i.e., respond “Stop” to the stimulus “Green” and “Go” to the stimulus “Red”). For the SGST, the average of median reaction times and the total number of correct responses across the mixed trials were calculated. Third, the Backward Digit Span Task was used to measure working memory span. Participants were asked to repeat strings of digits in reverse order and the scores were the highest number of digits achieved.

**Results**

**Randomization Check and Attrition Analyses**

Randomization was checked by comparing demographic variables and primary outcome variables at pretest between the two conditions. As shown in Supplementary Table 1, the treatment group was significantly younger (*t*(61.52) = 2.03, *p*  = .024, *d* = 0.46), included fewer people which described themselves as White (*X^2^*(1) = 8.45, *p*  = .004, *d* = 0.53), had lower scores in their self-reported functional health (*t*(76.15) = 2.57, *p*  = .006, *d* = 0.57). We controlled for these initial group differences in all multilevel analyses. We also checked for groups differences in terms of app usage during the 56 possible app usage days. Of all participants, 42.5% used the app every day. Participants of the comparison condition (*M* = 50.39 days, *SD* = 12.82) used the app on more days compared to the treatment group (*M* = 44.76 days, *SD* = 16.76); (*t*(75.97) = 1.70, *p*  = 0.047, *d* = 0.38). There were no significant initial differences between the two conditions in terms of sex, education, self-reported physical activity (MET’s), steps (baseline week), or self-control. See Supplementary Table 1 for more details, including the means and standard deviations for study variables at pretest, postttest, and follow-up and Supplementary Figure 1 for more details on enrollment and retention. Also, Supplementary Table 2 shows the bivariate correlations between all primary outcome measures. Attrition analyses were conducted to test differences between participants who completed all three measurement waves (85%) versus participants who completed one or two assessments only (15%). The results of independent *t*-tests revealed that they did not differ in demographic and primary outcome variables assessed at T1.

**Changes in Secondary Outcome Measures**

As shown in Supplementary Table 3, the treatment group had a higher initial score in life satisfaction (*t*(78) = -1.69, *p*  = .048, *d* = -0.38), a higher initial count in the Word List Recall Task (*t*(78) = -2.66, *p*  = .005, *d* = -0.60), and had a higher initial count of correct responses in the Stop & Go Switch Task (*t*(47.72) = -1.628, *p*  = .055, *d* = -0.47). There were no significant initial differences between the two conditions in terms of the Big Five traits, exercise self-efficacy, personal mastery, perceived constraints, or in the reaction time in the Stop & Go Switch Task.

Supplementary Table 3 shows the descriptive statistics, effect sizes across time and stability coefficients for all secondary outcome measures. First, we examined changes over time in all secondary outcome measures. Supplementary Tables 4 and 5 show the results of the multilevel models. The findings indicate a significant overall decrease in agreeableness, exercise self-efficacy, and the Backward Digit Span Test, and a significant overall increase in life satisfaction. In a next step, we examined whether the two groups differed in their changes in the secondary outcome measures. Supplementary Tables 6 and 7 show the results of the Time by Condition interaction effects. The two groups did not differ in changes in secondary outcome measures with one exception. The comparison group showed a greater increase in their scores in the Word List Recall Task.

Supplementary Table 1. *Descriptive statistics and effect sizes across time*

|  |  | T1 | | | T2 | | | T3 | | | Effect size across time | | | Stability coefficients | | |
| --- | --- | --- | --- | --- | --- | --- | --- | --- | --- | --- | --- | --- | --- | --- | --- | --- |
| Outcome | Condition | *N* | *M (%)* | *SD* | *N* | *M* | *SD* | *N* | *M* | *SD* | *d_T1,T2_* | *d_T2,T3_* | *d_T1,T3_* | *r_T1,T2_* | *r_T2,T3_* | *r_T1,T3_* |
| Self-control | Treatment | 42 | 3.32 | 0.60 | 37 | 3.54 | 0.63 | 36 | 3.63 | 0.65 | 0.36 | 0.23 | 0.63 | .49** | .80** | .66** |
|  | Control | 38 | 3.42 | 0.75 | 35 | 3.62 | 0.76 | 34 | 3.63 | 0.75 | 0.46 | 0.03 | 0.41 | .83** | .92** | .77** |
| MET’s | Treatment | 38 | 1598.95 | 2461.04 | 35 | 3268.70 | 3754.73 | 32 | 3891.36 | 6362.59 | 0.52 | 0.27 | 0.68 | .16 | .81** | .07 |
|  | Control | 33 | 2460.11 | 4157.84 | 29 | 3122.72 | 3811.26 | 33 | 2778.18 | 3586.78 | 0.21 | -0.17 | 0.09 | .70** | .86** | .64** |
| Steps | Treatment | 36 | 6184.45 | 3084.61 | 37 | 6647.71 | 2787.96 | 35 | 6426.23 | 3167.05 | 0.24 | -0.13 | 0.12 | .81** | .82** | .77** |
|  | Control | 38 | 6671.81 | 4213.50 | 38 | 6932.25 | 6071.56 | 35 | 6722.13 | 4638.01 | 0.11 | -0.10 | 0.02 | .85** | .94** | .86** |
| MVPA | Treatment | 36 | 19.81 | 18.19 | 37 | 22.37 | 18.91 | 35 | 19.89 | 19.65 | 0.17 | -0.19 | 0.01 | .65** | .75** | .67** |
|  | Control | 38 | 29.78 | 47.97 | 38 | 32.92 | 13.94 | 35 | 29.05 | 41.93 | 0.12 | -0.53 | -0.03 | .84** | .86** | .87** |
| Age | Treatment | 42 | 44.90^♣^ | 7.16 |  |  |  |  |  |  |  |  |  |  |  |  |
|  | Control | 38 | 49.24^♣^ | 11.28 |  |  |  |  |  |  |  |  |  |  |  |  |
| Sex (Female) | Treatment | 42 | 78.6 | |  |  |  |  |  |  |  |  |  |  |  |  |
|  | Control | 38 | 76.3 | |  |  |  |  |  |  |  |  |  |  |  |  |
| Education | Treatment | 42 | 16.71 | 2.87 |  |  |  |  |  |  |  |  |  |  |  |  |
|  | Control | 36 | 16.28 | 2.89 |  |  |  |  |  |  |  |  |  |  |  |  |
| Race (White) | Treatment | 42 | 54.8^♣^ | |  |  |  |  |  |  |  |  |  |  |  |  |
|  | Control | 38 | 78.9^♣^ | |  |  |  |  |  |  |  |  |  |  |  |  |
| Health status^1^ | Treatment | 41 | 31.68 | 2.91 |  |  |  |  |  |  |  |  |  |  |  |  |
|  | Control | 39 | 30.50 | 3.23 |  |  |  |  |  |  |  |  |  |  |  |  |
| Functional health | Treatment | 42 | 55.60^♣^ | 22.85 |  |  |  |  |  |  |  |  |  |  |  |  |
|  | Control | 38 | 67.24^♣^ | 17.62 |  |  |  |  |  |  |  |  |  |  |  |  |

*Note.* Intent-to-treat sample; T1 = Pretest, T2 = Posttest, T3 = Follow-up; ^1^ Higher score = worse self-rated health. Steps/MVPA: T1 = Average of baseline week, T2 = Average between pretest and posttest, T3 = Average between posttest and follow-up. Effect size across time take correlation into account (Morris & DeShon, 2008). Treatment = Self-control treatment group, Control = Comparison group.

** *p* < 0.01; ^♣^ Significant group differences at T1.

Supplementary Table 2. *Bivariate Pearson correlations between primary outcome measures*

|  | 1 | 2 | 3 | 4 | 5 | 6 | 7 | 8 | 9 | 10 | 11 | 12 | 13 | 14 | 15 | 16 | 17 | 18 | 19 | 20 |
| --- | --- | --- | --- | --- | --- | --- | --- | --- | --- | --- | --- | --- | --- | --- | --- | --- | --- | --- | --- | --- |
| 1. Condition | - |  |  |  |  |  |  |  |  |  |  |  |  |  |  |  |  |  |  |  |
| 2. Age | -.23* | - |  |  |  |  |  |  |  |  |  |  |  |  |  |  |  |  |  |  |
| 3. Sex | .03 | -.06 | - |  |  |  |  |  |  |  |  |  |  |  |  |  |  |  |  |  |
| 4. Education | .08 | -.24* | .02 | - |  |  |  |  |  |  |  |  |  |  |  |  |  |  |  |  |
| 5. Race | .26* | -.29** | -.12 | .12 | - |  |  |  |  |  |  |  |  |  |  |  |  |  |  |  |
| 6. Dose | -.19 | .14 | -.04 | -.06 | .03 | - |  |  |  |  |  |  |  |  |  |  |  |  |  |  |
| 7. Self-rated Health | .18 | .06 | -.07 | -.14 | -.13 | -.07 | - |  |  |  |  |  |  |  |  |  |  |  |  |  |
| 8. Functional Health | -.28 | .17 | -.04 | .03 | .03 | -.05 | .10 | - |  |  |  |  |  |  |  |  |  |  |  |  |
| 9. T1 Self-control | -.07 | .25* | -.07 | -.11 | .04 | .00 | .02 | .37** | - |  |  |  |  |  |  |  |  |  |  |  |
| 10. T2 Self-control | -.06 | .18 | -.12 | -.17 | .05 | .05 | -.02 | .13 | .69** | - |  |  |  |  |  |  |  |  |  |  |
| 11. T3 Self-control | .00 | .17 | -.11 | -.10 | -.05 | -.02 | .05 | .20 | .72** | .87** | - |  |  |  |  |  |  |  |  |  |
| 12. T1 MET’s | -.13 | .11 | .06 | -.10 | -.03 | .05 | -.01 | .11 | .11 | .10 | .07 | - |  |  |  |  |  |  |  |  |
| 13. T2 MET’s | .02 | -.12 | .08 | -.11 | -.14 | -.18 | .02 | .04 | .06 | .13 | .13 | .47** | - |  |  |  |  |  |  |  |
| 14.T3 MET’s | .11 | -.17 | -.02 | -.05 | -.12 | -.40** | .20 | .02 | .09 | .22 | .25* | .30* | .79** | - |  |  |  |  |  |  |
| 15. T1 Steps | -.07 | 0.10 | .08 | -.04 | .05 | -.15 | -.01 | .13 | .16 | .15 | -.00 | .16 | .19 | .19 | - |  |  |  |  |  |
| 16. T2 Steps | -.04 | .10 | .18 | -.04 | -.01 | -.13 | .06 | .16 | .15 | .14 | .12 | .18 | .22 | .27* | .83** | - |  |  |  |  |
| 17. T3 Steps | -.04 | .05 | .16 | -.03 | -.06 | -.27* | .14 | .08 | .02 | .17 | .13 | .17 | .32* | .40** | .83** | .90** | - |  |  |  |
| 18. T1 MVPA | -.14 | .19 | -.11 | -.15 | -.09 | .10 | .09 | -.08 | -.08 | .11 | -.10 | .25* | .13 | .14 | .74** | .65** | .65** | - |  |  |
| 19. T2 MVPA | -.13 | .10 | -.02 | -.15 | -.13 | .13 | .07 | -.06 | -.07 | .10 | -.00 | .55** | .34** | .29** | .57** | .74** | .67** | .82** | - |  |
| 20. T3 MVPA | -.14 | .32** | -.08 | -.17 | -.21 | .10 | .07 | -.10 | -.05 | .27* | .11 | .22 | .24 | .26* | .66** | .72** | .79** | .84** | .84** | - |

*Note.* T1 = Pretest, T2 = Posttest, T3 = Follow-up; PA = Physical Activity; Steps/MVPA: T1 = Average of baseline week, T2 = Average between pretest and posttest, T3 = Average between posttest and follow-up.

* *p* < .05, ** *p* < 0.01.

Supplementary Table 3. *Descriptive statistics and effect size across time for secondary outcome measures*

|  |  | Pretest | | | Posttest | | | Follow-up | | | Effect size across time | | | Stability coefficients | | |
| --- | --- | --- | --- | --- | --- | --- | --- | --- | --- | --- | --- | --- | --- | --- | --- | --- |
| Outcome | Condition | *N* | *M* | *SD* | *N* | *M* | *SD* | *N* | *M* | *SD* | *d_T1,T2_* | *d_T2,T3_* | *d_T1,T3_* | *r_T1,T2_* | *r_T2,T3_* | *r_T1,T3_* |
| Extraversion | Treatment | 42 | 3.41 | .72 | 37 | 3.49 | .65 | 36 | 3.44 | .68 | 0.17 | -0.13 | 0.06 | .79** | .81** | .71** |
|  | Control | 38 | 3.34 | .86 | 35 | 3.43 | .87 | 34 | 3.35 | .93 | 0.19 | -0.22 | 0.03 | .85** | .91** | .92** |
| Agreeableness | Treatment | 42 | 3.95 | .62 | 37 | 3.90 | .59 | 39 | 3.70 | .63 | -0.11 | -0.42 | -0.53 | .75** | .68** | .71** |
|  | Control | 38 | 3.89 | .66 | 35 | 3.85 | .63 | 35 | 3.78 | .63 | -0.09 | -0.19 | -0.32 | .75** | .83** | .86** |
| Conscientiousness | Treatment | 42 | 3.87 | .72 | 37 | 3.92 | .66 | 39 | 3.88 | .67 | 0.10 | -0.08 | 0.02 | .75** | .67** | .62** |
|  | Control | 38 | 3.90 | .80 | 35 | 3.89 | .81 | 35 | 3.78 | .71 | -0.02 | -0.29 | -0.32 | .84** | .89** | .89** |
| Neuroticism | Treatment | 42 | 2.82 | .91 | 37 | 2.80 | .88 | 36 | 2.76 | .89 | -0.04 | -0.09 | -0.09 | .83** | .87** | .71** |
|  | Control | 38 | 2.90 | 1.00 | 35 | 2.82 | .89 | 34 | 2.83 | .94 | -0.16 | 0.03 | -0.15 | .87** | .93** | .89** |
| Open-Mindedness | Treatment | 42 | 4.07 | .54 | 37 | 3.93 | .69 | 36 | 3.92 | .63 | -0.29 | -0.02 | -0.39 | .60** | .76** | .75** |
|  | Control | 38 | 4.04 | .82 | 35 | 4.05 | .76 | 34 | 3.99 | .82 | 0.03 | -0.25 | -0.14 | .88** | .95** | .90** |
| Exercise Self-efficacy | Treatment | 42 | 2.43 | .89 | 37 | 2.33 | .78 | 36 | 2.26 | .81 | -0.10 | -0.13 | -0.21 | .33** | .76** | .58** |
|  | Control | 37 | 2.48 | .95 | 35 | 2.15 | .80 | 34 | 2.14 | .74 | -0.54 | -0.02 | -0.38 | .79** | .73** | .56** |
| Life Satisfaction | Treatment | 42 | 4.55^♣^ | 1.40 | 36 | 4.64 | 1.57 | 36 | 4.75 | 1.41 | 0.11 | 0.14 | 0.83 | .84** | .88** | .83** |
|  | Control | 38 | 4.00^♣^ | 1.49 | 35 | 4.24 | 1.61 | 34 | 4.08 | 1.58 | 0.40 | -0.22 | 0.11 | .92** | .90** | .89** |
| Personal Mastery | Treatment | 42 | 5.64 | 1.20 | 37 | 5.59 | 1.08 | 36 | 5.75 | .98 | -0.06 | 0.20 | 0.10 | .78** | .73** | .61** |
|  | Control | 38 | 5.75 | 1.27 | 35 | 5.66 | 1.03 | 34 | 5.41 | 1.32 | -0.09 | -0.35 | -0.36 | .66** | .76** | .72** |
| Perceived Constraints | Treatment | 42 | 3.33 | 1.35 | 37 | 3.35 | 1.44 | 36 | 3.25 | 1.52 | 0.02 | -0.12 | -0.10 | .76** | .83** | .81** |
|  | Control | 38 | 3.23 | 1.34 | 35 | 3.32 | 1.43 | 34 | 3.48 | 1.44 | 0.07 | 0.14 | 0.26 | .54** | .67** | .75** |
| Word List Recall | Treatment | 42 | 7.57^♣^ | 3.17 | 37 | 6.84 | 2.44 | 36 | 7.42 | 2.70 | -0.23 | 0.21 | -0.04 | .51** | .38* | .37* |
|  | Control | 38 | 5.84^♣^ | 2.58 | 35 | 6.69 | 3.37 | 34 | 7.41 | 2.80 | 0.30 | 0.23 | 0.54 | .30 | .57** | .36* |
| Stop & Go Switch Reaction Time | Treatment | 41 | .88 | .22 | 37 | .94 | .36 | 37 | .88 | .23 | 0.24 | -0.16 | 0.00 | .34* | .44** | .65** |
|  | Control | 34 | .90 | .22 | 35 | .97 | .32 | 34 | .94 | .23 | 0.36 | -0.12 | 0.18 | .60** | .68** | .50** |
| Stop & Go Switch Count | Treatment | 41 | 27.32^♣^ | 3.07 | 38 | 24.08 | 6.19 | 37 | 26.46 | 4.20 | -0.86 | 0.31 | -0.33 | .25 | .25 | .64** |
|  | Control | 36 | 24.72^♣^ | 7.48 | 36 | 25.14 | 6.30 | 34 | 25.44 | 4.53 | 0.05 | 0.06 | 0.08 | .26 | .71** | .19 |
| Backward Digit Span | Treatment | 42 | 5.17 | 2.84 | 37 | 4.30 | 3.13 | 36 | 4.11 | 3.11 | -0.33 | -0.07 | -0.37 | .58** | .59** | .50** |
|  | Control | 38 | 4.63 | 2.81 | 35 | 4.17 | 2.66 | 34 | 3.50 | 3.06 | -0.18 | -0.24 | -0.34 | .57** | .44** | .29 |

*Note.* Treatment = self-control treatment condition; Control = comparison condition.

* *p* < .05, ** *p* < 0.01, ****p* < .001.

^♣^ Significant group differences at T1.

Supplementary Table 4. *Changes over time in personality outcome measures, exercise self-efficacy and life satisfaction*

| Fixed effects | Extraversion | | Agreeableness | | Conscientiousness | | Neuroticism | | Open-Mindedness | | Exercise Self-Efficacy | | Life Satisfaction | |
| --- | --- | --- | --- | --- | --- | --- | --- | --- | --- | --- | --- | --- | --- | --- |
| Intercept |  |  |  |  |  |  |  |  |  |  |  |  |  |  |
| Estimate (SE) | 3.39*** (0.10) | 3.81** (1.22) | 4.02*** (0.08) | 3.20** (0.94) | 3.93*** (0.09) | 3.70** (1.12) | 2.90*** (0.11) | 5.22*** (1.26) | 4.09*** (0.09) | 3.82*** (1.09) | 2.51*** (0.12) | 2.16 (1.19) | 4.23*** (0.18) | 1.94 (2.21) |
| 95% CI | 3.20; 3.58 | 1.39; 6.22 | 3.86; 4.17 | 1.33; 5.07 | 3.75; 4.11 | 1.48; 5.93 | 2.68; 3.12 | 2.73; 7.72 | 3.92; 4.27 | 1.64; 6.00 | 2.28; 2.74 | -0.21; 4.52 | 3.87; 4.59 | -2.45; 6.32 |
| Time |  |  |  |  |  |  |  |  |  |  |  |  |  |  |
| Estimate (SE) | 0.01 (0.03) | 0.01 (0.03) | -0.09*** (0.02) | -0.09*** (0.03) | -0.03 (0.03) | -0.02 (0.03) | -0.04 (0.03) | -0.05 (0.03) | -0.04 (0.03) | -0.03 (0.02) | -0.10* (0.04) | -0.09* (0.04) | 0.09* (0.04) | 0.11* (0.04) |
| 95% CI | -0.04; 0.06 | -0.04; 0.06 | -0.14; -0.04 | -0.14; -0.04 | -0.08; 0.03 | -0.07; 0.04 | -0.10; 0.02 | -0.11; 0.01 | -0.09; 0.01 | -0.08; 0.02 | -0.19; -0.02 | -0.18; -0.01 | 0.01; 0.19 | 0.02; 0.20 |

*Note*. Intent-to-treat sample; Left column: Results without covariates; Right column: Results with covariates (age, sex, education, race, self-reported functional health, and self-reported health status); Condition: 1 = Self-control treatment group, 0 = Comparison group.

* *p* < .05, ** *p* < 0.01, ****p* < .001.

Supplementary Table 5. *Changes over time in sense of control and cognition*

| Fixed effects | Personal Mastery | | Perceived Constraints | | Word List Recall | | Stop & Go Switch Reaction Time | | Stop & Go Switch Count | | Backward Digit Span | |
| --- | --- | --- | --- | --- | --- | --- | --- | --- | --- | --- | --- | --- |
| Intercept |  |  |  |  |  |  |  |  |  |  |  |  |
| Estimate (SE) | 5.75*** (0.15) | 7.82*** (1.65) | 3.24*** (0.18) | 4.60* (1.89) | 6.36*** (0.44) | 0.80 (3.67) | 0.90*** (0.04) | 1.06** (0.34) | 26.30*** (0.83) | 20.55** (6.88) | 5.45*** (0.42) | 5.63 (4.14) |
| 95% CI | 5.44; 6.05 | 4.54; 11.09 | 2.88; 3.61 | 0.86; 8.35 | 5.50; 7.22 | -6.48; 8.09 | 0.82; 0.98 | 0.38; 1.73 | 24.65; 27.95 | 6.88; 34.18 | 4.61; 6.28 | -2.59; 13.84 |
| Time |  |  |  |  |  |  |  |  |  |  |  |  |
| Estimate (SE) | -0.05 (0.05) | -0.04 (0.05) | 0.04 (0.06) | 0.05 (0.06) | 0.32 (0.18) | 0.21 (0.17) | 0.00 (0.02) | 0.00 (0.02) | -0.34 (0.36) | -0.32 (0.37) | -0.55** (0.17) | -0.50** (0.17) |
| 95% CI | -0.16; 0.05 | -0.15; 0.06 | -0.08; 0.17 | -0.08; 0.17 | -0.04; 0.68 | -0.12; 0.55 | -0.03; 0.04 | -0.03; 0.03 | -1.04; 0.37 | -1.04; 0.41 | -0.88; -0.21 | -0.84; -0.16 |

*Note.* Intent-to-treat sample; Left column: Results without covariates; Right column: Results with covariates (age, sex, education, race, self-reported functional health, and self-reported health status); Condition: 1 = Self-control treatment group, 0 = Comparison group.

* *p* < .05, ** *p* < 0.01, ****p* < .001.

Supplementary Table 6. *Changes over time in personality outcome measures, exercise self-efficacy and life satisfaction between conditions*

| Fixed effects | Extraversion | | Agreeableness | | Conscientiousness | | Neuroticism | | Open-Mindedness | | Exercise Self-Efficacy | | Life Satisfaction | |
| --- | --- | --- | --- | --- | --- | --- | --- | --- | --- | --- | --- | --- | --- | --- |
| Intercept |  |  |  |  |  |  |  |  |  |  |  |  |  |  |
| Estimate (SE) | 3.35*** (0.14) | 3.94** (1.21) | 3.95*** (0.11) | 3.14** (0.94) | 3.98*** (0.13) | 3.84*** (1.12) | 2.95*** (0.16) | 5.25*** (1.26) | 4.06*** (0.13) | 3.78*** (1.10) | 2.56*** (0.17) | 2.39* (1.19) | 4.01*** (0.26) | 2.39 (2.17) |
| 95% CI | 3.08; 3.63 | 1.54; 6.35 | 3.72; 4.18 | 1.26; 5.02 | 3.71; 4.24 | 1.62; 6.06 | 2.63; 3.27 | 2.74; 7.76 | 3.81; 4.31 | 1.59; 5.96 | 2.22; 2.89 | 0.01; 4.75 | 3.49; 4.52 | -1.91; 6.68 |
| Time |  |  |  |  |  |  |  |  |  |  |  |  |  |  |
| Estimate (SE) | 0.02 (0.04) | 0.00 (0.04) | -0.06 (0.04) | -0.06 (0.04) | -0.06 (0.04) | -0.04 (0.04) | -0.05 (0.04) | -0.05 (0.04) | -0.02 (0.04) | -0.01 (0.04) | -0.16* (0.06) | -0.15* (0.06) | 0.05 (0.06) | 0.07 (0.07) |
| 95% CI | -0.32; 0.44 | -0.07; 0.08 | -0.13; 0.01 | -0.14; 0.01 | -0.14; 0.02 | -0.12; 0.04 | -0.13; 0.04 | -0.14; 0.03 | -0.09; 0.06 | -0.08; 0.07 | -0.28; -0.04 | -0.27; -0.02 | -0.08; 0.17 | -0.06; 0.19 |
| Condition |  |  |  |  |  |  |  |  |  |  |  |  |  |  |
| Estimate (SE) | 0.06 (0.19) | 0.19 (0.21) | 0.12 (0.16) | 0.08 (0.17) | -0.09 (0.18) | 0.08 (0.20) | -0.08 (0.22) | 0.00 (0.22) | 0.06 (0.18) | 0.13 (0.19) | -0.09 (0.23) | 0.01 (0.24) | 0.42 (0.36) | 0.47 (0.36) |
| 95% CI | -0.32; 0.44 | -0.21; 0.60 | -0.19; 0.44 | -0.26; 0.42 | -0.45; 0.27 | -0.31; 0.04 | -0.52; 0.36 | -0.43; 0.43 | -0.29; 0.40 | -0.25; 0.50 | -0.55; 0.37 | -0.47; 0.49 | -0.29; 1.13 | -0.24; 1.19 |
| Time by condition |  |  |  |  |  |  |  |  |  |  |  |  |  |  |
| Estimate (SE) | 0.01 (0.05) | 0.01 (0.05) | -0.05 (0.05) | -0.05 (0.05) | 0.06 (0.06) | 0.04 (0.06) | 0.01 (0.06) | 0.01 (0.06) | -0.04 (0.05) | -0.05 (0.05) | 0.11 (0.09) | 0.10 (0.09) | 0.09 (0.09) | 0.08 (0.09) |
| 95% CI | -0.09; 0.11 | -0.09; 0.12 | -0.15; 0.05 | -0.15; 0.05 | -0.05; 0.17 | -0.07; 0.15 | -0.11; 0.13 | -0.11; 0.13 | -0.14; 0.05 | -0.15; 0.04 | -0.06; 0.28 | -0.07; 0.27 | -0.08; 0.27 | -0.10; 0.26 |

*Note.* Intent-to-treat sample; Left column: Results without covariates; Right column: Results with covariates (age, sex, education, race, self-reported functional health, and self-reported health status); Condition: 1 = Self-control treatment group, 0 = Ccomparison group.

* *p* < .05, ** *p* < 0.01, ****p* < .001.

Supplementary Table 7. *Changes over time in sense of control and cognition between conditions*

| Fixed effects | Personal Mastery | | Perceived Constraints | | Word List Recall | | Stop & Go Switch Reaction Time | | Stop & Go Switch Count | | Backward Digit Span | | |
| --- | --- | --- | --- | --- | --- | --- | --- | --- | --- | --- | --- | --- | --- |
| Intercept |  |  |  |  |  |  |  |  |  |  |  |  |  |
| Estimate (SE) | 5.92*** (0.22) | 8.15*** (1.65) | 3.12*** (0.27) | 4.43* (1.90) | 5.12*** (0.62) | 0.30 (3.69) | 0.90*** (0.06) | 1.02** (0.34) | 25.47*** (1.22) | 20.28** (6.95) | 5.21*** (0.61) | 5.72 (4.15) |  |
| 95% CI | 5.49; 6.36 | 4.87; 11.41 | 2.60; 3.65 | -0.07; 0.29 | 3.90; 6.34 | -7.01; 7.62 | -0.03; 0.06 | 0.35; 1.69 | 23.06; 27.88 | 6.48; 34.04 | 4.01; 6.42 | -2.53; 13.96 |  |
| Time |  |  |  |  |  |  |  |  |  |  |  |  |  |
| Estimate (SE) | -0.15* (0.08) | -0.15 (0.08) | 0.11 (0.09) | 0.11 (0.09) | 0.77** (0.26) | 0.61* (0.24) | 0.02 (0.02) | 0.01 (0.02) | -0.10 (0.52) | -0.05 (0.54) | -0.53* (0.24) | -0.39 (0.25) |  |
| 95% CI | -0.30; -0.00 | -0.30; 0.00 | -0.06; 0.28 | -0.07; 0.29 | 0.27; 1.27 | 0.13; 1.09 | -0.03; 0.06 | -0.04; 0.05 | -1.13; 0.94 | -1.13; 1.02 | -1.02; -0.05 | -0.89; 0.10 |  |
| Condition |  |  |  |  |  |  |  |  |  |  |  |  |  |
| Estimate (SE) | -0.34 (0.30) | -0.18 (0.32) | 0.23 (0.36) | 0.15 (0.37) | 2.38** (0.86) | 1.86* (0.84) | 0.01 (0.08) | -0.01 (0.08) | 1.55 (1.67) | 1.39 (1.76) | 0.44 (0.85) | 0.86 (0.90) |  |
| 95% CI | -0.94; 0.26 | -0.80; 0.45 | -0.49; 0.95 | -0.58; 0.87 | 0.69; 4.07 | 0.20; 3.51 | -0.15; 0.17 | -0.17; 0.15 | -1.75; 4.84 | -2.08; 4.85 | -1.23; 2.12 | -0.91; 2.64 |  |
| Time by condition |  |  |  |  |  |  |  |  |  |  |  |  |  |
| Estimate (SE) | 0.20 (0.11) | 0.20 (0.11) | -0.12 (0.12) | -0.12 (0.12) | -0.87* (0.36) | -0.75* (0.33) | -0.02 (0.03) | -0.01 (0.03) | -0.43 (0.72) | -0.48 (0.74) | -0.03 (0.34) | -0.20 (0.34) |  |
| 95% CI | -0.01; 0.40 | -0.01; 0.41 | -0.37; 0.12 | -0.37; 0.12 | -1.57; -0.17 | -1.41; -0.09 | -0.09; 0.04 | -0.07; 0.05 | -1.85; 0.98 | -1.93; 0.97 | -0.70; 0.65 | -0.88; 0.47 |  |

*Note*. Intent-to-treat sample; Left column: Results without covariates; Right column: Results with covariates (age, sex, education, race, self-reported functional health, and self-reported health status); Condition: 1 = Self-control treatment group, 0 = Comparison group.

* *p* < .05, ** *p* < 0.01, ****p* < .001.

Supplementary Figure 1. *CONSORT Flow Diagram*


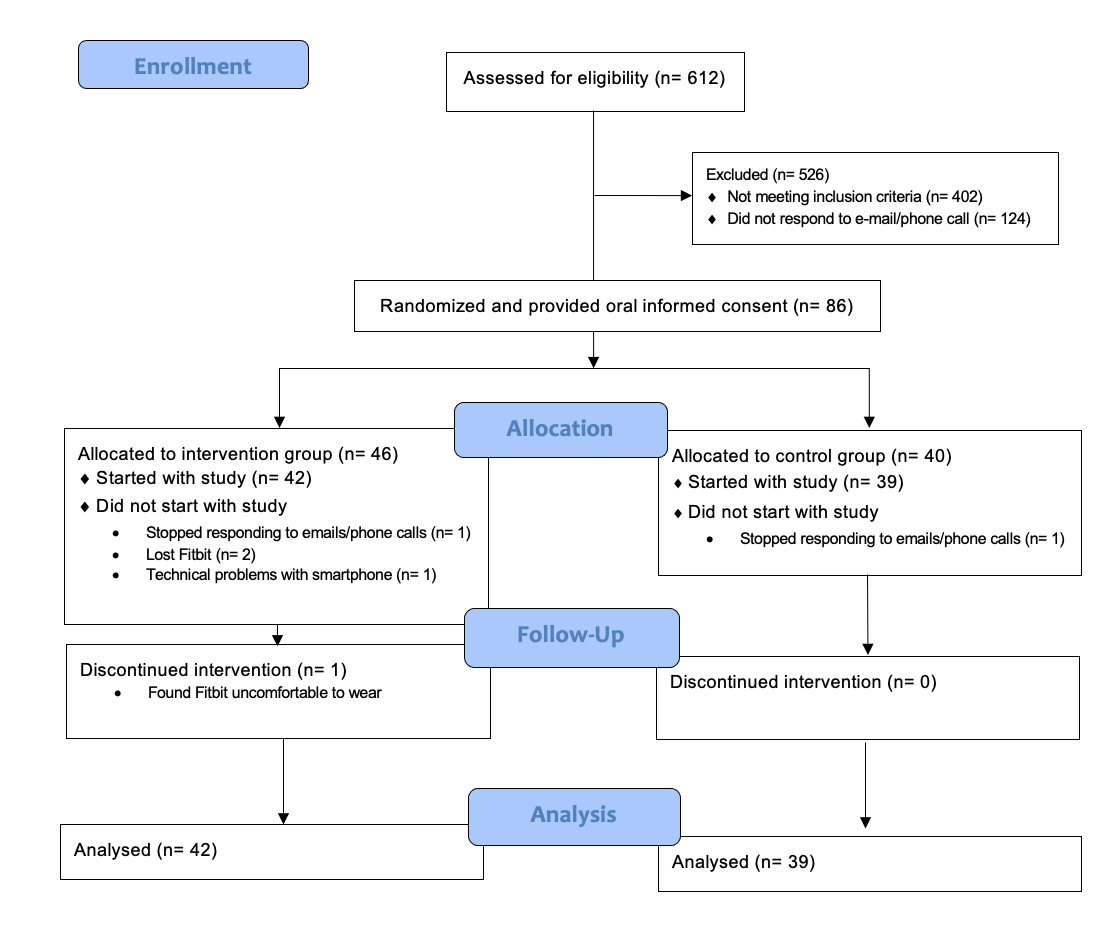


*Note.* Intervention group = self-control treatment condition; Control group = comparison condition.

Supplementary Figure 2. *Changes in MET’s between conditions*


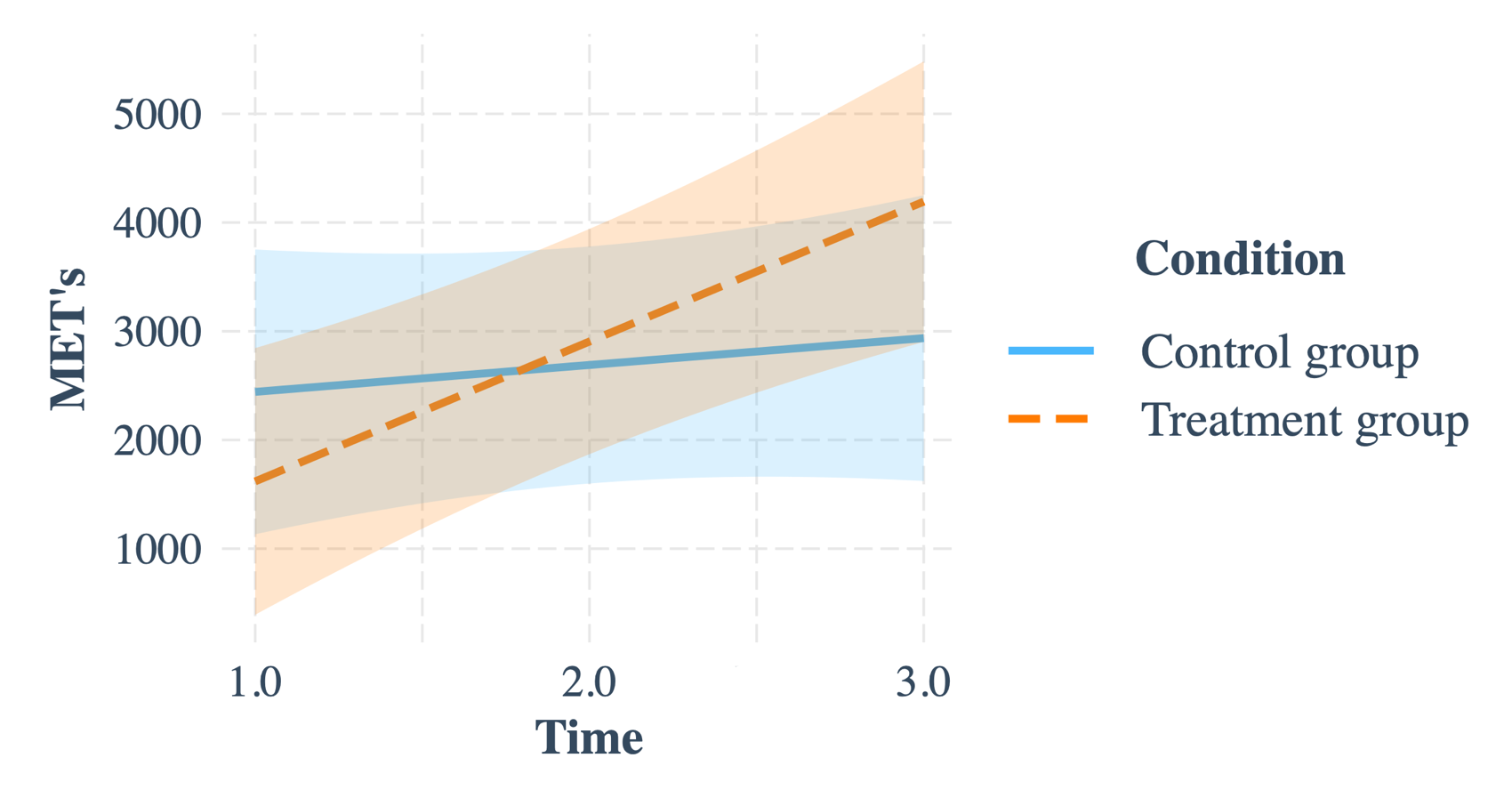


*Note.* Treatment group = self-control treatment condition; Control group = comparison condition; X-axis: 1 = pretest, 2 = posttest, 3 = follow-up; Shaded areas represent 95% CIs.
