# Supplementary material for: Effects of a digital self-control intervention to increase physical activity in middle-aged adults
Source: J Health Psychol. 2023 Apr 12;28(10):984–96. doi: 10.1177/13591053231166756 (PMC10466994; doi:10.1177/13591053231166756)
Supplement: sj-pdf-1-hpq-10.1177_13591053231166756 – Supplemental material for Effects of a digital self-control intervention to increase physical activity in middle-aged adults [file sj-pdf-1-hpq-10.1177_13591053231166756.pdf]

```r
library(foreign)

StepData = read.spss("DailyData_FigShare.sav",
                       to.data.frame = TRUE,
                       reencode = TRUE,
                       use.value.labels = FALSE)
library(interactions)
library(nlme)
library(lme4)
library(lmerTest)
library(psych)
library(multilevel)
library(dplyr)
library(ggplot2)
library(interplot)
library(cowplot)
library(jtools)
library("RColorBrewer")
library(reghelper)
library(sjstats)
library(longpower)
display.brewer.all()
library(MuMIn)
library(ggeffects)
display.brewer.all()
library(lsmeans)


StepData$Condition <- as.factor(StepData$Condition)
StepData$WhiteNonWhite <- as.factor(StepData$WhiteNonWhite)

Untilendofintervention<-filter(StepData, Day < 56)

####Overall Changes in Daily Steps during intervention###

###With covariates##

Steps1 <- lmer(Steps ~  Day + Age+ Gender + WhiteNonWhite + EducYrs + Gen_health_pre + GHQ_Comp_pre +
                 (1 | id),
               data = Untilendofintervention,
               REML = FALSE,
               na.action = "na.omit")


summary(Steps1)
confint(Steps1)
AIC(Steps1)
BIC(Steps1)
effectsize::standardize_parameters(Steps1)

###Without covariates##

Steps2 <- lmer(Steps ~  Day +
                 (1 | id),
               data = Untilendofintervention,
               REML = FALSE,
               na.action = "na.omit")


summary(Steps2)
confint(Steps2)
AIC(Steps2)
BIC(Steps2)
effectsize::standardize_parameters(Steps2)

####Overall Changes in MVPA during intervention###

###With covariates##

Steps3 <- lmer(MVPA ~  Day + Age+ Gender + WhiteNonWhite + EducYrs + Gen_health_pre + GHQ_Comp_pre +
                 (1 | id),
               data = Untilendofintervention,
               REML = FALSE,
               na.action = "na.omit")


summary(Steps3)
confint(Steps3)
AIC(Steps3)
BIC(Steps3)

###Without covariates##

Steps4 <- lmer(MVPA ~  Day +
                 (1 | id),
               data = Untilendofintervention,
               REML = FALSE,
               na.action = "na.omit")


summary(Steps4)
confint(Steps4)
AIC(Steps4)
BIC(Steps4)


####Differential changes between conditions in daily steps###

###With covariates##

Steps5 <- lmer(Steps ~  Day*Condition + Age+ Gender + WhiteNonWhite + EducYrs + Gen_health_pre + GHQ_Comp_pre +
                 (1 | id),
               data = Untilendofintervention,
               REML = FALSE,
               na.action = "na.omit")


summary(Steps5)
confint(Steps5)
AIC(Steps5)
BIC(Steps5)

###Without covariates##

Steps6 <- lmer(Steps ~  Day*Condition +
                 (1 | id),
               data = Untilendofintervention,
               REML = FALSE,
               na.action = "na.omit")


summary(Steps6)
confint(Steps6)
AIC(Steps6)
BIC(Steps6)

####Differential changes between conditions in MVPA###
```

```
###With covariates##

Steps7 <- lmer(MVPA ~  Day*Condition + Age+ Gender + WhiteNonWhite + EducYrs + Gen_health_pre + GHQ_Comp_pre +
                (1 | id),
              data = Untilendofintervention,
              REML = FALSE,
              na.action = "na.omit")


summary(Steps7)
confint(Steps7)
AIC(Steps7)
BIC(Steps7)


###Without covariates##

Steps8 <- lmer(MVPA ~  Day*Condition +
                (1 | id),
              data = Untilendofintervention,
              REML = FALSE,
              na.action = "na.omit")


summary(Steps8)
confint(Steps8)
AIC(Steps8)
BIC(Steps8)



#######Effects of changes in self-control on changes in daily steps by condition#######

###With covariates##

Steps9 <- lmer(Steps ~  Day*Change_Selfcontrol*Condition + Age+ Gender + WhiteNonWhite + EducYrs + Gen_health_pre + GHQ_Comp_pre +
                (1 | IDtoMerge),
              data = Untilendofintervention,
              REML = FALSE,
              na.action = "na.omit")


summary(Steps9)
confint(Steps9)
AIC(Steps9)
BIC(Steps9)

###Without covariates##

Steps10 <- lmer(Steps ~  Day*Change_Selfcontrol*Condition +
                (1 | IDtoMerge),
              data = Untilendofintervention,
              REML = FALSE,
              na.action = "na.omit")


summary(Steps10)
confint(Steps10)
AIC(Steps10)
BIC(Steps10)

#######Effects of changes in self-control on changes in MVPA by condition#######

###With covariates##

Steps11 <- lmer(MVPA ~  Day*Change_Selfcontrol*Condition + Age+ Gender + WhiteNonWhite + EducYrs + Gen_health_pre + GHQ_Comp_pre +
                (1 | id),
              data = Untilendofintervention,
              REML = FALSE,
              na.action = "na.omit")


summary(Steps11)
confint(Steps11)
AIC(Steps11)
BIC(Steps11)

###Without covariates##

Steps12 <- lmer(MVPA ~  Day*Change_Selfcontrol*Condition +
              data = Untilendofintervention,
              REML = FALSE,
              na.action = "na.omit")


summary(Steps12)
confint(Steps12)
AIC(Steps12)
BIC(Steps12)

####Effects of T1 Conscientiousness on changes in daily steps###

###With covariates##

Steps13 <- lmer(Steps ~  Day*B5_Con_pre*Condition + Age + Gender + WhiteNonWhite + EducYrs + Gen_health_pre + GHQ_Comp_pre +
                (1 | id),
              data = Untilendofintervention,
              REML = FALSE,
              na.action = "na.omit")


summary(Steps13)
confint(Steps13)
AIC(Steps13)
BIC(Steps13)

###Without covariates##

Steps14 <- lmer(Steps ~  Day*B5_Con_pre*Condition +
                (1 | id),
              data = Untilendofintervention,
              REML = FALSE,
              na.action = "na.omit")


summary(Steps14)
confint(Steps14)
AIC(Steps14)
BIC(Steps14)


####Effects of T1 Conscientiousness on changes in MVPA###

###With covariates##

Steps15 <- lmer(MVPA ~  Day*B5_Con_pre + Age+ Gender + WhiteNonWhite + EducYrs + Gen_health_pre + GHQ_Comp_pre +
```

```
                (1 | IDtoMerge),
              data = Untilendofintervention,
              REML = FALSE,
              na.action = "na.omit")


summary(Steps15)
confint(Steps15)
AIC(Steps15)
BIC(Steps15)

###Without covariates##

Steps16 <- lmer(MVPA ~  Day*B5_Con_pre +
                (1 | IDtoMerge),
              data = Untilendofintervention,
              REML = FALSE,
              na.action = "na.omit")


summary(Steps16)
confint(Steps16)
AIC(Steps16)
BIC(Steps16)
```
