# Supplementary material for: Effects of a digital self-control intervention to increase physical activity in middle-aged adults
Source: J Health Psychol. 2023 Apr 12;28(10):984–96. doi: 10.1177/13591053231166756 (PMC10466994; doi:10.1177/13591053231166756)
Supplement: sj-pdf-2-hpq-10.1177_13591053231166756 – Supplemental material for Effects of a digital self-control intervention to increase physical activity in middle-aged adults [file sj-pdf-2-hpq-10.1177_13591053231166756.pdf]

```
library(foreign)

Mindhike = read.spss("PrePostFollowLong_FigShare.sav",
                  to.data.frame = TRUE,
                  reencode = TRUE,
                  use.value.labels = FALSE)

library(interactions)
library(nlme)
library(lme4)
library(lmerTest)
library(psych)
library(multilevel)
library(dplyr)
library(ggplot2)
library(interplot)
library(cowplot)
library(jtools)
library("RColorBrewer")
library(reghelper)
library(sjstats)
library(longpower)
display.brewer.all()
library(MuMIn)
library(ggeffects)
display.brewer.all()
Mindhike$id <- as.factor(Mindhike$id)
Mindhike$Condition_pre <- as.factor(Mindhike$Condition_pre)
library(lsmeans)


####Overall changes in self control####

####With covariates####

Model1 <- lmer(Selfcontrol ~ Time + Age+ Gender + WhiteNonWhite + EducYrs + Gen_health_pre + GHQ_Comp_pre +
                  (1 | id),
                data = Mindhike,
                REML = FALSE,
                na.action = "na.omit")

summary(Model1)
confint(Model1)
rand(Model1)
AIC(Model1)
BIC(Model1)
r.squaredGLMM(Model1)

####Without covariates####

Model2 <- lmer(Selfcontrol ~ Time +
                 (1 | id),
               data = Mindhike,
               REML = FALSE,
               na.action = "na.omit")

summary(Model2)
confint(Model2)
rand(Model2)
AIC(Model2)
BIC(Model2)
r.squaredGLMM(Model2)

####Differential effects between conditions in change in self control####

####With covariates####

Model3 <- lmer(Selfcontrol ~ Time*Condition_pre +  Age+ Gender + WhiteNonWhite + EducYrs + Gen_health_pre + GHQ_Comp_pre +
                 (1 | id),
               data = Mindhike,
               REML = FALSE,
               na.action = "na.omit")

summary(Model3)
confint(Model3)
rand(Model3)
AIC(Model3)
BIC(Model3)
r.squaredGLMM(Model3)

####Without covariates####

Model4 <- lmer(Selfcontrol ~ Time*Condition_pre +
                 (1 | id),
               data = Mindhike,
               REML = FALSE,
               na.action = "na.omit")

summary(Model4)
confint(Model4)
rand(Model4)
AIC(Model4)
BIC(Model4)
r.squaredGLMM(Model4)

####Overall changes in METs####

####With covariates####

Model5 <- lmer(PhysicalActivity ~ Time  + Age+ Gender + WhiteNonWhite + EducYrs + Gen_health_pre + GHQ_Comp_pre +
                 (1 | id),
               data = Mindhike,
               REML = FALSE,
               na.action = "na.omit")

summary(Model5)
confint(Model5)
rand(Model5)
AIC(Model5)
BIC(Model5)
r.squaredGLMM(Model5)

####Without covariates####

Model6 <- lmer(PhysicalActivity ~ Time  +
                 (1 | id),
               data = Mindhike,
               REML = FALSE,
               na.action = "na.omit")

summary(Model6)
confint(Model6)
rand(Model6)
AIC(Model6)
BIC(Model6)
r.squaredGLMM(Model6)
```

```
####Differential effects between conditions in change in METs####

####With covariates####

Model7 <- lmer(PhysicalActivity ~ Time*Condition_pre  + Age+ Gender + WhiteNonWhite + EducYrs + Gen_health_pre + GHQ_Comp_pre +
                 (1 | id),
               data = Mindhike,
               REML = FALSE,
               na.action = "na.omit")

summary(Model7)
confint(Model7)
rand(Model7)
AIC(Model7)
BIC(Model7)
r.squaredGLMM(Model7)

####Without covariates####

Model8 <- lmer(PhysicalActivity ~ Time*Condition_pre +
                 (1 | id),
               data = Mindhike,
               REML = FALSE,
               na.action = "na.omit")

summary(Model8)
confint(Model8)
rand(Model8)
AIC(Model8)
BIC(Model8)
r.squaredGLMM(Model8)

####Effect of changes in self-control on changes in METs####

####With covariates####

Model9 <- lmer(PhysicalActivity ~ Time*Change_Selfcontrol +  Age+ Gender + WhiteNonWhite + EducYrs + Gen_health_pre + GHQ_Comp_pre +
                 (1 | id),
               data = Mindhike,
               REML = FALSE,
               na.action = "na.omit")

summary(Model9)
confint(Model9)
rand(Model9)
AIC(Model9)
BIC(Model9)
r.squaredGLMM(Model9)

####Without covariates####

Model10 <- lmer(PhysicalActivity ~ Time*Change_Selfcontrol +
                 (1 | id),
               data = Mindhike,
               REML = FALSE,
               na.action = "na.omit")

summary(Model10)
confint(Model10)
rand(Model10)
AIC(Model10)
BIC(Model10)
r.squaredGLMM(Model10)



####Effect of T1 conscientiousness on changes in METs####

####With covariates####

Model11 <- lmer(PhysicalActivity ~ Time*Condition_pre*B5_Con_pre  + Age+ Gender + WhiteNonWhite + EducYrs + Gen_health_pre +
                 (1 | id),
               data = Mindhike,
               REML = FALSE,
               na.action = "na.omit")

summary(Model11)
confint(Model11)
rand(Model11)
AIC(Model11)
BIC(Model11)
r.squaredGLMM(Model11)

####Without covariates####

Model12 <- lmer(PhysicalActivity ~ Time*Condition_pre*B5_Con_pre  +
                 (1 | id),
               data = Mindhike,
               REML = FALSE,
               na.action = "na.omit")

summary(Model12)
confint(Model12)
rand(Model12)
AIC(Model12)
BIC(Model12)
r.squaredGLMM(Model12)


####Differential effect between conditions of changes in self-control on changes in METs####

####With covariates####

Model13 <- lmer(PhysicalActivity ~ Time*Change_Selfcontrol*Condition_pre +  Age+ Gender + WhiteNonWhite + EducYrs + Gen_health_pre + GHQ_Comp_pre +
                 (1 | id),
               data = Mindhike,
               REML = FALSE,
               na.action = "na.omit")

summary(Model13)
confint(Model13)
rand(Model13)
AIC(Model13)
BIC(Model13)
r.squaredGLMM(Model13)

####Without covariates####

Model14 <- lmer(PhysicalActivity ~ Time*Change_Selfcontrol*Condition_pre +
                 (1 | id),
               data = Mindhike,
               REML = FALSE,
               na.action = "na.omit")

summary(Model14)
confint(Model14)
rand(Model14)
```

```
AIC(Model14)
BIC(Model14)
r.squaredGLMM(Model14)


####Differential effects between conditions of T1 conscientiousness on changes in METs####

####With covariates####

Model15 <- lmer(PhysicalActivity ~ Time*Condition_pre*B5_Con_pre*Condition_pre  + Age+ Gender + WhiteNonWhite + EducYrs + Gen_health_pre +
                  (1 | id),
               data = Mindhike,
               REML = FALSE,
               na.action = "na.omit")

summary(Model15)
confint(Model15)
rand(Model15)
AIC(Model15)
BIC(Model15)
r.squaredGLMM(Model15)

####Without covariates####

Model16 <- lmer(PhysicalActivity ~ Time*Condition_pre*B5_Con_pre*Condition_pre  +
                  (1 | id),
               data = Mindhike,
               REML = FALSE,
               na.action = "na.omit")

summary(Model16)
confint(Model16)
rand(Model16)
AIC(Model16)
BIC(Model16)
r.squaredGLMM(Model16)
```
