# Supplementary material for: Effects of a digital self-control intervention to increase physical activity in middle-aged adults
Source: J Health Psychol. 2023 Apr 12;28(10):984–96. doi: 10.1177/13591053231166756 (PMC10466994; doi:10.1177/13591053231166756)
Supplement: sj-pdf-3-hpq-10.1177_13591053231166756 – Supplemental material for Effects of a digital self-control intervention to increase physical activity in middle-aged adults [file sj-pdf-3-hpq-10.1177_13591053231166756.pdf]

Codebook Dataset "PrePostFollowWide_FigShare"

Pretest - Demographics			
Variable Name	Variable Label	Values	Recode?
Gender	Gender - Selected Choice	1 = Male<br>2 = Female<br>3 = Prefer to self-describe<br>4 = I do not wish to answer<br><br>22.5% Male (N=18)<br>77.5% Female (N=62)<br>N=80	No
Gender_3_TEXT	Gender - Prefer to self-describe - Text	Manual Entry	No
Race	Which category best describes your race?	1 = American Indian<br>2 = Asian<br>3 = Black/African American<br>4 = Native Hawaiian<br>5 = White<br>6 = More than one race<br>7 = I do not wish to answer<br><br>American Indian 0% (N=0)<br>Asian 10% (N=8)<br>Black/African American 13.8% (N=11)<br>White 71.3% (N=57)<br>More than one race 5% (N=4)<br>N=80	No
Ethnicity	Which category best describes your ethnicity/ethnic origin?	1 = Hispanic or Latin American<br>2 = Not Hispanic<br>3 = I do not wish to answer<br><br>Hispanic of Latin American 5% (N=4)<br>Not Hispanic 95% (N=76)<br>N=80	No
Education	How many years of education have you had?<br>(For example: 12 years = finished high school, 16 years = finished college, 18 years = finished 2 year	4 = How many years of education have you have<br>5 = Not applicable	No

	graduate program, etc.) - Selected Choice		
Education_4_TEXT	How many years of education have you had?<br><br>(For example: 12 years = finished high school, 16 years = finished college, 18 years = finished 2 year graduate program, etc.) - How many years of eduction have you had? - Text	Range: 12 – 26<br><br>M = 16.51<br>Max = 26<br>Min = 12<br>SD = 2.873<br>SE = -.325<br>N = 78<br><br>12 (N=6)<br>13 (N=3)<br>14 (N=8)<br>15 (N=4)<br>16 (N=29)<br>17 (N=8)<br>18 (N=9)<br>19 (N=1)<br>20 (N=2)<br>21 (N=1)<br>22 (N=3)<br>23 (N=2)<br>25 (N=1)<br>26 (N=1)	No
**Education_2**	What is the highest degree or level of education you have completed?	1 = Some grade school (1-6)<br>2 = Eighth grade/junior high school (7-8)<br>3 = Some high school (9-12, no diploma, no GED)<br>4 = GED<br>5 = Graduated from high school<br>6 = 1-2 years of college, no degree yet<br>7 = 3 or more years of college, no degree yet<br>8 = 2 year college, vocational school, or Associate’s degree<br>9 = Bachelor’s degree<br>10 = Some graduate school<br>11 = Master’s degree<br>12 = PhD, EdD, MD, DDS, LLB, LLD, JD, or other doctoral level professional degree<br>13 = Not applicable<br><br>M = 8.64<br>Max = 12<br>Min = 1	13=Missing

		SD = 2.206<br>SE = 0.247<br>N = 80<br><br>1 (N=1)<br>2 (N=0)<br>3 (N=0)<br>4 (N=1)<br>5 (N=5)<br>6 (N=10)<br>7 (N=3)<br>8 (N=9)<br>9 (N=28)<br>10 (N=4)<br>11 (N=12)<br>12 (N=7)	
**Marital_Status**	Which category best describes your relationship status?	1 = Single<br>2 = Married<br>3 = Separated<br>4 = Divorced<br>5 = Widowed<br>6 = With a long-term partner<br>7 = Not applicable<br><br>M = 2.46<br>Max = 6<br>Min = 1<br>SD = 1.567<br>SE = 0.175<br>N = 80<br><br>1 (N=20)<br>2 (N=41)<br>3 (N=1)<br>4 (N=7)<br>5 (N= 2)<br>6 (N= 9)<br>7 (N=0)	7 = Missing
Employment_1	Which category best describes your current employment situation? (Check all that apply) - Selected Choice	1 = Working now full time<br><br>N=39	No
Employment_2	Which category best describes your current employment situation? (Check all that apply) - Selected Choice	1 = Working now part time<br><br>N=17	No
Employment_3	Which category best describes your current employment situation? (Check all that apply) - Selected Choice	1 = Self-employed<br><br>N=6	No

Employment_4	Which category best describes your current employment situation? (Check all that apply) - Selected Choice	1 = Unemployed and looking for work<br><br>N=5	No
Employment_5	Which category best describes your current employment situation? (Check all that apply) - Selected Choice	1 = Temporarily laid off<br><br>N=1	No
Employment_6	Which category best describes your current employment situation? (Check all that apply) - Selected Choice	1 = Retired<br><br>N=3	No
Employment_7	Which category best describes your current employment situation? (Check all that apply) - Selected Choice	1 = Homemaker<br><br>N=10	No
Employment_8	Which category best describes your current employment situation? (Check all that apply) - Selected Choice	1 = Full time student<br><br>N=1	No
Employment_9	Which category best describes your current employment situation? (Check all that apply) - Selected Choice	1 = Part time student<br><br>N=1	No
Employment_10	Which category best describes your current employment situation? (Check all that apply) - Selected Choice	1 = Maternity/sick leave<br><br>N=0	No
Employment_11	Which category best describes your current employment situation? (Check all that apply) - Selected Choice	1 = Permanently disabled<br><br>N=5	No
Employment_12	Which category best describes your current employment situation? (Check all that apply) - Selected Choice	1 = Don’t know/not sure<br><br>N=0	No

Employment_13	Which category best describes your current employment situation? (Check all that apply) - Selected Choice	1 = Other – please specify<br><br>N=1	No
**Employment_14**	Which category best describes your current employment situation? (Check all that apply) - Selected Choice	1 = Not applicable	1 = Missing
Employment_13_TEXT	Which category best describes your current employment situation? (Check all that apply) - Other - please specify - Text	Manual Entry	No
**Income**	What is your total annual household income, considering all sources including wages, interest, social security, dividends, and other payments? - Selected Choice	4 = Total annual household income<br>5 = Not applicable	5 = Missing
Income_4_TEXT	What is your total annual household income, considering all sources including wages, interest, social security, dividends, and other payments? - Total annual household income - Text	Manual entry<br>Range: 10,000 – 800,000<br>N=72<br>M = 81898.50<br>Max = 800000<br>Min = 10000<br>SD = 96228.411<br>SE = 11340.627	No
**Height**	What is your height?<br><br>(For example: 5 ft 7 in = 5'7") - Selected Choice	4 = Height<br>5 = Not Applicable	5 = Missing
Height_4_TEXT	What is your height?<br><br>(For example: 5 ft 7 in = 5'7") - Height - Text	Manual Entry<br>Range: 4’10” – 6’5”	No
**Weight**	What is your current weight? (in pounds) - Selected Choice	4 = Current Weight<br>5 = Not Applicable	5 = Missing

Weight_4_TEXT	What is your current weight? (in pounds) - Current weight - Text	Manual Entry<br>Range: 94 – 435<br><br>M = 178.38<br>Max = 435<br>Min = 94<br>SD = 55.226<br>SE = 6.213<br>N = 79	No

**Functional Health<br>36-Item Short Form Survey (SF-36)**			
Variable Names:<br>• **Pretest**<br>• **Posttest**<br>• **Follow-up**	Variable Label	Values	Recode?
**FH1<br>Q479_post<br>Q492**	In general, would you say your health is:	1 = Excellent<br>2 = Very Good<br>3 = Good<br>4 = Fair<br>5 = Poor<br>6 = Not applicable	Yes<br>Recode to<br>1 = 100<br>2 = 75<br>3 = 50<br>4 = 25<br>5 = 0<br>6 = Missing<br><br>Pre: M=53.7500<br>SE=2.43994<br>SD=21.82352 Min=0<br>Max=100 N=80<br>Post: M=59.5890<br>SE=2.56341<br>SD=21.90182 Min=0<br>Max=100 N=73<br>Follow: M=58.3333<br>SE=2.42251<br>SD=20.55566 Min=25<br>Max=100 N=72
**FH2<br>Q463<br>Q493**	Compared to one year ago, how would your rate your health in general now?	1 = Much better now than one year ago<br>2 = Somewhat better now than one year ago<br>3 = About the same<br>4 = Somewhat worse now than one year ago<br>5 = Much worse now than one year ago<br>6 = Not applicable	Yes<br>Recode to<br>1 = 100<br>2 = 75<br>3 = 50<br>4 = 25<br>5 = 0<br>6 = Missing<br><br>Pre: M=45.9375<br>SE=2.53316<br>SD=22.65724 Min=0<br>Max=100 N=80

			Post: M=56.1644<br>SE=2.61625<br>SD=22.35323 Min=25<br>Max=100 N=73<br>Follow: M=59.3750<br>SE=2.72812<br>SD=23.14890 Min=25<br>Max=100 N=72
**FH3_1**<br>**Q481_1**<br>**Q494_1**	The following items are about activities you might do during a typical day. Does your health now limit you in these activities? If so, how much? - Vigorous activities, such as running, lifting heavy objects, participating in strenuous sports		Yes<br>Recode to<br>1 = 0<br>2 = 50<br>3 = 100<br>4 = Missing<br><br>Pre: M=62.5000<br>SE=4.50390<br>SD=39.26406 Min=0<br>Max=100 N=76<br>Post: M=59.2857<br>SE=4.80390<br>SD=40.19234 Min=0<br>Max=100 N=70<br>Follow: M=66.4179<br>SE=4.91769<br>SD=40.25304 Min=0<br>Max=100 N=67
**FH3_2**<br>**Q481_2**<br>**Q494_2**	The following items are about activities you might do during a typical day. Does your health now limit you in these activities? If so, how much? - Moderate activities, such as moving a table, pushing a vacuum cleaner, bowling, or playing golf	1 = Yes, limited a lot<br>2 = Yes, limited a little<br>3 = No, not limited at all<br>4 = Not applicable	Yes<br>Recode to<br>1 = 0<br>2 = 50<br>3 = 100<br>4 = Missing<br><br>Pre: M=87.9747<br>SE=2.73449<br>SD=24.30468 Min=0<br>Max=100 N=79<br>Post: M=88.0282<br>SE=2.91820<br>SD=24.58918 Min=0<br>Max=100 N=71<br>Follow: M=86.2319<br>SE=3.24544<br>SD=26.95865 Min=0<br>Max=100 N=69
**FH3_3**<br>**Q481_3**<br>**Q494_3**	The following items are about activities you might do during a typical day. Does your health now limit you in	1 = Yes, limited a lot<br>2 = Yes, limited a little<br>3 = No, not limited at all<br>4 = Not applicable	Yes<br>Recode to<br>1 = 0<br>2 = 50<br>3 = 100

	these activities? If so, how much? - Lifting or carrying groceries		4 = Missing<br><br>Pre: M=88.6076 SE=2.69476 SD=23.95156 Min=0 Max=100 N=79<br>Post: M=89.5833 SE=2.78632 SD=23.64273 Min=0 Max=100 N=72<br>Follow: M=89.8551 SE=2.84205 SD=23.60787 Min=0 Max=100 N=69
**FH3_4**<br>**Q481_4**<br>**Q494_4**	The following items are about activities you might do during a typical day. Does your health now limit you in these activities? If so, how much? - Climbing several flights of stairs	1 = Yes, limited a lot<br>2 = Yes, limited a little<br>3 = No, not limited at all<br>4 = Not applicable	Yes<br>Recode to<br>1 = 0<br>2 = 50<br>3 = 100<br>4 = Missing<br><br>Pre: M=77.8481 SE=3.45918 SD=30.74589 Min=0 Max=100 N=79<br>Post: M=83.3333 SE=3.42594 SD=29.07009 Min=0 Max=100 N=72<br>Follow: M=83.3333 SE=3.52598 SD=29.28896 Min=0 Max=100 N=69
**FH3_5**<br>**Q481_5**<br>**Q494_5**	The following items are about activities you might do during a typical day. Does your health now limit you in these activities? If so, how much? - Climbing one flight of stairs	1 = Yes, limited a lot<br>2 = Yes, limited a little<br>3 = No, not limited at all<br>4 = Not applicable	Yes<br>Recode to<br>1 = 0<br>2 = 50<br>3 = 100<br>4 = Missing<br><br>Pre: M=92.9487 SE=2.18301 SD=19.27982 Min=0 Max=100 N=78<br>Post: M=95.8333 SE=1.91516 SD=16.25068 Min=0 Max=100 N=72<br>Follow: M=92.5373 SE=2.65916 SD=21.76620 Min=0 Max=100 N=67

**FH3_6**<br>**Q481_6**<br>**Q494_6**	The following items are about activities you might do during a typical day. Does your health now limit you in these activities? If so, how much? - Bending, kneeling, or stooping	1 = Yes, limited a lot<br>2 = Yes, limited a little<br>3 = No, not limited at all<br>4 = Not applicable	Yes<br>Recode to<br>1 = 0<br>2 = 50<br>3 = 100<br>4 = Missing<br><br>Pre: M=81.6456<br>SE=3.14339<br>SD=27.93905 Min=0<br>Max=100 N=79<br>Post: M=81.9444<br>SE=3.86914<br>SD=32.83072 Min=0<br>Max=100 N=72<br>Follow: M=81.8841<br>SE=3.99422<br>SD=33.17846 Min=0<br>Max=100 N=69
**FH3_7**<br>**Q481_7**<br>**Q494_7**	The following items are about activities you might do during a typical day. Does your health now limit you in these activities? If so, how much? - Walking more than a mile	1 = Yes, limited a lot<br>2 = Yes, limited a little<br>3 = No, not limited at all<br>4 = Not applicable	Yes<br>Recode to<br>1 = 0<br>2 = 50<br>3 = 100<br>4 = Missing<br><br>Pre: M=88.6076<br>SE=2.84133<br>SD=25.25431 Min=0<br>Max=100 N=79<br>Post: M=88.1944<br>SE=2.88218<br>SD=24.45613 Min=0<br>Max=100 N=72<br>Follow: M=86.9565<br>SE=3.20714<br>SD=26.64055 Min=0<br>Max=100 N=69
**FH3_8**<br>**Q481_8**<br>**Q494_8**	The following items are about activities you might do during a typical day. Does your health now limit you in these activities? If so, how much? - Walking several blocks	1 = Yes, limited a lot<br>2 = Yes, limited a little<br>3 = No, not limited at all<br>4 = Not applicable	Yes<br>Recode to<br>1 = 0<br>2 = 50<br>3 = 100<br>4 = Missing<br><br>Pre: M=94.2308<br>SE=1.82044<br>SD=16.07767 Min=50<br>Max=100 N=78<br>Post: M=92.2535<br>SE=2.38369<br>SD=20.08533 Min=0<br>Max=100 N=71

			Follow: M=92.6471<br>SE=2.62207<br>SD=21.62210 Min=0<br>Max=100 N=68
**FH3_9**<br>**Q481_9**<br>**Q494_9**	The following items are about activities you might do during a typical day. Does your health now limit you in these activities? If so, how much? - Walking one block	1 = Yes, limited a lot<br>2 = Yes, limited a little<br>3 = No, not limited at all<br>4 = Not applicable	Yes<br>Recode to<br>1 = 0<br>2 = 50<br>3 = 100<br>4 = Missing<br><br>Pre: M=96.2025<br>SE=1.49980<br>SD=13.33049 Min=50<br>Max=100 N=79<br>Post: M=95.8333<br>SE=1.91516<br>SD=16.25068 Min=0<br>Max=100 N=72<br>Follow: M=94.8529<br>SE=2.37499<br>SD=19.58469 Min=0<br>Max=100 N=68
**FH3_10**<br>**Q481_10**<br>**Q494_10**	The following items are about activities you might do during a typical day. Does your health now limit you in these activities? If so, how much? - Bathing or dressing yourself	1 = Yes, limited a lot<br>2 = Yes, limited a little<br>3 = No, not limited at all<br>4 = Not applicable	Yes<br>Recode to<br>1 = 0<br>2 = 50<br>3 = 100<br>4 = Missing<br><br>Pre: M=97.4684<br>SE=1.24124<br>SD=11.03239 Min=50<br>Max=100 N=79<br>Post: M=98.6111<br>SE=0.97515<br>SD=8.27444 Min=50<br>Max=100 N=72<br>Follow: M=94.7761<br>SE=2.40945<br>SD=19.72218 Min=0<br>Max=100 N=67
**FH4_1**<br>**Q465_1**<br>**Q495_1**	During the past 4 weeks, have you had any of the following problems with your work or other regular daily activities as a result of your physical health? - Cut down the amount of time you spent on work or other activities	1 = Yes<br>2 = No<br>3 = Not applicable	Yes<br>Recode to<br>1 = 0<br>2 = 100<br>3 = Missing<br><br>Pre: M=79.2208<br>SE=4.65401<br>SD=40.83878 Min=0<br>Max=100 N=77

			Post: M=80.8824<br>SE=4.80404<br>SD=39.61514 Min=0<br>Max=100 N=68<br>Follow: M=85.5072<br>SE=4.26896<br>SD=35.46068 Min=0<br>Max=100 N=69
**FH4_2**<br>**Q465_2**<br>**Q495_2**	During the past 4 weeks, have you had any of the following problems with your work or other regular daily activities as a result of your physical health? - Accomplished less than you would like	1 = Yes<br>2 = No<br>3 = Not applicable	Yes<br>Recode to<br>1 = 0<br>2 = 100<br>3 = Missing<br><br>Pre: M=54.4304<br>SE=5.63912<br>SD=50.12157 Min=0<br>Max=100 N=79<br>Post: M=71.4286<br>SE=5.43848<br>SD=45.50158 Min=0<br>Max=100 N=70<br>Follow: M=78.2609<br>SE=5.00195<br>SD=41.54928 Min=0<br>Max=100 N=69
**FH4_3**<br>**Q465_3**<br>**Q495_3**	During the past 4 weeks, have you had any of the following problems with your work or other regular daily activities as a result of your physical health? - Were limited in the kind of work or other activities	1 = Yes<br>2 = No<br>3 = Not applicable	Yes<br>Recode to<br>1 = 0<br>2 = 100<br>3 = Missing<br><br>Pre: M=74.6667<br>SE=5.05584<br>SD=43.78490 Min=0<br>Max=100 N=75<br>Post: M=78.8732<br>SE=4.87902<br>SD=41.11132 Min=0<br>Max=100 N=71<br>Follow: M=82.6087<br>SE=4.59647<br>SD=38.18115 Min=0<br>Max=100 N=69
**FH4_4**<br>**Q465_4**<br>**Q495_4**	During the past 4 weeks, have you had any of the following problems with your work or other regular daily activities as a result of your physical health? - Had difficulty performing work or	1 = Yes<br>2 = No<br>3 = Not applicable	Yes<br>Recode to<br>1 = 0<br>2 = 100<br>3 = Missing<br><br>Pre: M=71.4286<br>SE=5.18197

	other activities (for example, it took extra effort)		SD=45.47163 Min=0 Max=100 N=77<br>Post: M=77.4648 SE=4.99383 SD=42.07878 Min=0 Max=100 N=71<br>Follow: M=80.0000 SE=4.81543 SD=40.28881 Min=0 Max=100 N=70
**FH5_1**<br>**Q467_1**<br>**Q496_1**	During the past 4 weeks, have you had any of the following problems with your work or other regular daily activities as a result of any emotional problems (such as feeling depressed or anxious)? - Cut down the amount of time you spent on work or other activities	1 = Yes<br>2 = No<br>3 = Not applicable	Yes<br>Recode to<br>1 = 0<br>2 = 100<br>3 = Missing<br><br>Pre: M=63.6364 SE=5.51797 SD=48.42001 Min=0 Max=100 N=77<br>Post: M=74.2857 SE=5.26157 SD=44.02145 Min=0 Max=100 N=70<br>Follow: M=77.4648 SE=4.99383 SD=42.07878 Min=0 Max=100 N=71
**FH5_2**<br>**Q467_2**<br>**Q496_2**	During the past 4 weeks, have you had any of the following problems with your work or other regular daily activities as a result of any emotional problems (such as feeling depressed or anxious)? - Accomplished less than you would like	1 = Yes<br>2 = No<br>3 = Not applicable	Yes<br>Recode to<br>1 = 0<br>2 = 100<br>3 = Missing<br><br>Pre: M=53.8462 SE=5.68115 SD=50.17452 Min=0 Max=100 N=78<br>Post: M=62.8571 SE=5.81688 SD=48.66755 Min=0 Max=100 N=70<br>Follow: M=63.3803 SE=5.75818 SD=5.75818 Min=0 Max=100 N=71
**FH5_3**<br>**Q467_3**<br>**Q496_3**	During the past 4 weeks, have you had any of the following problems with your work or other regular daily activities as a result of any emotional	1 = Yes<br>2 = No<br>3 = Not applicable	Yes<br>Recode to<br>1 = 0<br>2 = 100<br>3 = Missing

	problems (such as feeling depressed or anxious)? - Didn't do work or other activities as carefully as usual		Pre: M=57.8947<br>SE=5.70108<br>SD=49.70086 Min=0<br>Max=100 N=76<br>Post: M=71.0145<br>SE=5.50186<br>SD=45.70188 Min=0<br>Max=100 N=69<br>Follow: M=83.0986<br>SE=4.47929<br>SD=37.74318 Min=0<br>Max=100 N=71
**FH6**<br>**Q473**<br>**Q497**	During the past 4 weeks, to what extent has your physical health or emotional problems interfered with your normal social activities with family, friends, neighbors, or groups?	1 = Not at all<br>2 = Slightly<br>3 = Moderately<br>4 – Quite a bit<br>5 = Extremely<br>6 = Not applicable	Yes<br>Recode to<br>1 = 100<br>2 = 75<br>3 = 50<br>4 = 25<br>5 = 0<br>6 = Missing<br><br>Pre: M=71.1538<br>SE=3.06295<br>SD=27.05121 Min=0<br>Max=100 N=78<br>Post: M=80.7143<br>SE=2.74764<br>SD=22.98843 Min=0<br>Max=100 N=70<br>Follow: M=78.7879<br>SE=2.81740<br>SD=22.88870 Min=0<br>Max=100 N=66
**FH7**<br>**Q475**<br>**Q498**	How much bodily pain have you had during the past 4 weeks?	1= None<br>2 = Very Mild<br>3 = Mild<br>4 = Moderate<br>5 = Severe<br>6 = Very Severe<br>7 = Not applicable	Yes<br>Recode to<br>1 = 100<br>2 = 80<br>3 = 60<br>4 = 40<br>5 = 20<br>6 = 0<br>7 = Missing<br><br>Pre: M=69.7500<br>SE=2.49033<br>SD=22.27418 Min=0<br>Max=100 N=80<br>Follow: M=70.1449<br>SE=3.07960<br>SD=25.58106 Min=0<br>Max=100 N=69
**FH8**<br>**Q469**	During the past 4 weeks, how much did	1 = Not at all<br>2 = A little bit	Yes<br>Recode to

**Q499**	pain interfere with your normal work (including both work outside the home and housework)?	3 = Moderately<br>4 = Quite a bit<br>5 = Extremely<br>6= Not applicable	1 = 100<br>2 = 75<br>3 = 50<br>4 = 25<br>5 = 0<br>6 = Missing<br><br>Pre: M=82.5949 SE=2.12949 SD=18.92734 Min=25 Max=100 N=79<br>Post: M=81.4286 SE=2.67676 SD=22.39537 Min=25 Max=100 N=70<br>Follow: M=80.3030 SE=3.17126 SD=25.76340 Min=0 Max=100 N=66
**FH9_1**<br>**Q483_1**<br>**Q500_1**	These questions are about how you feel and how things have been with you during the past 4 weeks. For each question, please give the one answer that comes closest to the way you have been feeling.<br><br>How much of the time during the past 4 weeks... - Did you feel full of pep?	1 = All of the time<br>2 = Most of the time<br>3 = A good bit of the time<br>4 = Some of the time<br>5 = A little of the time<br>6 = None of the time<br>7 = Not applicable	Yes<br>Recode to<br>1 = 100<br>2 = 80<br>3 = 60<br>4 = 40<br>5 = 20<br>6 = 0<br>7 = Missing<br><br>Pre: M=39.7500 SE=2.57775 SD=23.05607 Min=0 Max=100 N=80<br>Post: M=48.3333 SE=2.61661 SD=22.20265 Min=0 Max=100 N=72<br>Follow: M=46.5714 SE=2.72163 SD=22.77080 Min=0 Max=100 N=70
**FH9_2**<br>**Q483_2**<br>**Q500_2**	These questions are about how you feel and how things have been with you during the past 4 weeks. For each question, please give the one answer that comes closest to the way you have been feeling.<br><br>How much of the time during the past 4	1 = All of the time<br>2 = Most of the time<br>3 = A good bit of the time<br>4 = Some of the time<br>5 = A little of the time<br>6 = None of the time<br>7 = Not applicable	Yes<br>Recode to<br>1 = 0<br>2 = 20<br>3 = 40<br>4 = 60<br>5 = 80<br>6 = 100<br>7 = Missing<br><br>Pre: M=66.8421 SE=3.00999

	weeks... - Have you been a very nervous person?		SD=26.24045 Min=0 Max=100 N=76<br>Post: M=72.5714 SE=2.91129 SD=24.35758 Min=0 Max=100 N=70<br>Follow: M=72.1739 SE=3.06834 SD=25.48757 Min=0 Max=100 N=69
**FH9_3**<br>**Q483_3**<br>**Q500_3**	These questions are about how you feel and how things have been with you during the past 4 weeks. For each question, please give the one answer that comes closest to the way you have been feeling.<br><br>How much of the time during the past 4 weeks... - Have you felt so down in the dumps that nothing could cheer you up?	1 = All of the time<br>2 = Most of the time<br>3 = A good bit of the time<br>4 = Some of the time<br>5 = A little of the time<br>6 = None of the time<br>7 = Not applicable	Yes<br>Recode to<br>1 = 0<br>2 = 20<br>3 = 40<br>4 = 60<br>5 = 80<br>6 = 100<br>7 = Missing<br><br>Pre: M=82.8205 SE=2.32905 SD=20.56964 Min=20 Max=100 N=78<br>Post: M=83.4783 SE=2.33424 SD=19.38966 Min=20 Max=100 N=69<br>Follow: M=80.8696 SE=2.68984 SD=22.34352 Min=20 Max=100 N=69
**FH9_4**<br>**Q483_4**<br>**Q500_4**	These questions are about how you feel and how things have been with you during the past 4 weeks. For each question, please give the one answer that comes closest to the way you have been feeling.<br><br>How much of the time during the past 4 weeks... - Have you felt calm and peaceful?	1 = All of the time<br>2 = Most of the time<br>3 = A good bit of the time<br>4 = Some of the time<br>5 = A little of the time<br>6 = None of the time<br>7 = Not applicable	Yes<br>Recode to<br>1 = 100<br>2 = 80<br>3 = 60<br>4 = 40<br>5 = 20<br>6 = 0<br>7 = Missing<br><br>Pre: M=46.7500 SE=2.66111 SD=23.80166 Min=0 Max=100 N=80<br>Post: M=49.7222 SE=2.85248 SD=24.20407 Min=0 Max=100 N=72<br>Follow: M=50.5714 SE=2.56498

			SD=21.46016 Min=20 Max=80 N=70
**FH9_5 Q483_5 Q500_5**	These questions are about how you feel and how things have been with you during the past 4 weeks. For each question, please give the one answer that comes closest to the way you have been feeling.<br><br>How much of the time during the past 4 weeks... - Did you have a lot of energy?	1 = All of the time<br>2 = Most of the time<br>3 = A good bit of the time<br>4 = Some of the time<br>5 = A little of the time<br>6 = None of the time<br>7 = Not applicable	Yes<br>Recode to<br>1 = 100<br>2 = 80<br>3 = 60<br>4 = 40<br>5 = 20<br>6 = 0<br>7 = Missing<br><br>Pre: M=39.5000 SE=2.58954 SD=23.16151 Min=0 Max=100 N=80<br>Post: M=49.7222 SE=2.40608 SD=20.41625 Min=0 Max=80 N=72<br>Follow: M=45.3521 SE=2.80723 SD=23.65411 Min=0 Max=100 N=71
**FH9_6 Q483_6 Q500_6**	These questions are about how you feel and how things have been with you during the past 4 weeks. For each question, please give the one answer that comes closest to the way you have been feeling.<br><br>How much of the time during the past 4 weeks... - Have you felt downhearted and blue?	1 = All of the time<br>2 = Most of the time<br>3 = A good bit of the time<br>4 = Some of the time<br>5 = A little of the time<br>6 = None of the time<br>7 = Not applicable	Yes<br>Recode to<br>1 = 0<br>2 = 20<br>3 = 40<br>4 = 60<br>5 = 80<br>6 = 100<br>7 = Missing<br><br>Pre: M=75.1282 SE=2.34221 SD=20.68588 Min=20 Max=100 N=78<br>Post: M=77.1014 SE=2.19569 SD=18.23873 Min=20 Max=100 N=69<br>Follow: M=76.8571 SE=2.46334 SD=20.60975 Min=20 Max=100 N=70
**FH9_7 Q483_7 Q500_7**	These questions are about how you feel and how things have been with you during the past 4 weeks. For each question, please give	1 = All of the time<br>2 = Most of the time<br>3 = A good bit of the time<br>4 = Some of the time<br>5 = A little of the time	Yes<br>Recode to<br>1 = 0<br>2 = 20<br>3 = 40<br>4 = 60

	the one answer that comes closest to the way you have been feeling.<br><br>How much of the time during the past 4 weeks... - Did you feel worn out?	6 = None of the time<br>7 = Not applicable	5 = 80<br>6 = 100<br>7 = Missing<br><br>Pre: M=54.3590 SE=2.89447 SD=25.56325 Min=0 Max=100 N=78<br>Post: M=61.6667 SE=2.92713 SD=24.83750 Min=0 Max=100 N=72<br>Follow: M=62.2857 SE=3.03309 SD=25.37667 Min=0 Max=100 N=70
**FH9_8**<br>**Q483_8**<br>**Q500_8**	These questions are about how you feel and how things have been with you during the past 4 weeks. For each question, please give the one answer that comes closest to the way you have been feeling.<br><br>How much of the time during the past 4 weeks... - Have you been a happy person?	1 = All of the time<br>2 = Most of the time<br>3 = A good bit of the time<br>4 = Some of the time<br>5 = A little of the time<br>6 = None of the time<br>7 = Not applicable	Yes<br>Recode to<br>1 = 100<br>2 = 80<br>3 = 60<br>4 = 40<br>5 = 20<br>6 = 0<br>7 = Missing<br><br>Pre: M=56.0000 SE=2.62413 SD=23.47096 Min=20 Max=100 N=80<br>Post: M=58.0556 SE=2.88430 SD=24.47412 Min=0 Max=100 N=72<br>Follow: M=58.0282 SE=2.57382 SD=21.68737 Min=20 Max=100 N=71
**FH9_9**<br>**Q483_9**<br>**Q500_9**	These questions are about how you feel and how things have been with you during the past 4 weeks. For each question, please give the one answer that comes closest to the way you have been feeling.<br><br>How much of the time during the past 4 weeks... - Did you feel tired?	1 = All of the time<br>2 = Most of the time<br>3 = A good bit of the time<br>4 = Some of the time<br>5 = A little of the time<br>6 = None of the time<br>7 = Not applicable	Yes<br>Recode to<br>1 = 0<br>2 = 20<br>3 = 40<br>4 = 60<br>5 = 80<br>6 = 100<br>7 = Missing<br><br>Pre: M=48.6076 SE=2.87540 SD=25.55708 Min=0 Max=100 N=79<br>Post: M=53.6111 SE=3.00824

			SD=25.52577 Min=0<br>Max=100 N=72<br>Follow: M=57.1429<br>SE=3.15994<br>SD=26.43794 Min=0<br>Max=100 N=70
**FH10**<br>**Q471**<br>**Q501**	During the past 4 weeks, how much of the time has your physical health or emotional problems interfered with your social activities (like visiting with friends, relatives, etc.)?	1 = All of the time<br>2 = Most of the time<br>3 = Some of the time<br>4 = A little of the time<br>5 = None of the time<br>6 = Not applicable	Yes<br>Recode to<br>1 = 0<br>2 = 25<br>3 = 50<br>4 = 75<br>5 = 100<br>6 = Missing<br><br>Pre: M=68.7500<br>SE=3.11071<br>SD=27.11857 Min=0<br>Max=100 N=76<br>Post: M=76.0870<br>SE=3.24127<br>SD=26.92404 Min=0<br>Max=100 N=69<br>Follow: M=75.3846<br>SE=3.31138<br>SD=26.69720 Min=0<br>Max=100 N=65
**FH11_1**<br>**Q477_1**<br>**Q502_1**	How TRUE or FALSE is each of the following statements for you. - I seem to get sick a little easier than other people	1 = Definitely true<br>2 = Mostly true<br>3 = Don't know<br>4 = Mostly false<br>5 = Definitely false<br>6 = Not applicable	Yes<br>Recode to<br>1 = 0<br>2 = 25<br>3 = 50<br>4 = 75<br>5 = 100<br>6 = Missing<br><br>Pre: M=78.1646<br>SE=3.22802<br>SD=28.69130 Min=0<br>Max=100 N=79<br>Post: M=76.4085<br>SE=3.20679<br>SD=27.02093 Min=0<br>Max=100 N=71<br>Follow: M=77.5362<br>SE=3.50621<br>SD=29.12477 Min=0<br>Max=100 N=69
**FH11_2**<br>**Q477_2**<br>**Q502_2**	How TRUE or FALSE is each of the following statements for you. - I	1 = Definitely true<br>2 = Mostly true<br>3 = Don't know<br>4 = Mostly false	Yes<br>Recode to<br>1 = 100<br>2 = 75

	am as healthy as anybody I know	5 = Definitely false<br>6 = Not applicable	3 = 50<br>4 = 25<br>5 = 0<br>6 = Missing<br><br>Pre: M=59.4937 SE=2.99788 SD=26.64576 Min=0 Max=100 N=79<br>Post: M=63.8889 SE=3.56202 SD=30.22477 Min=0 Max=100 N=72<br>Follow: M=60.5634 SE=3.42577 SD=28.86606 Min=0 Max=100 N=71
**FH11_3**<br>**Q477_3**<br>**Q502_3**	How TRUE or FALSE is each of the following statements for you. - I expect my health to get worse	1 = Definitely true<br>2 = Mostly true<br>3 = Don't know<br>4 = Mostly false<br>5 = Definitely false<br>6 = Not applicable	Yes<br>Recode to<br>1 = 0<br>2 = 25<br>3 = 50<br>4 = 75<br>5 = 100<br>6 = Missing<br><br>Pre: M=63.6076 SE=3.54062 SD=31.46973 Min=0 Max=100 N=79<br>Post: M=63.2143 SE=3.40204 SD=28.46350 Min=0 Max=100 N=70<br>Follow: M=62.5000 SE=3.55197 SD=29.71788 Min=0 Max=100 N=70
**FH11_4**<br>**Q477_4**<br>**Q502_4**	How TRUE or FALSE is each of the following statements for you. - My health is excellent	1 = Definitely true<br>2 = Mostly true<br>3 = Don't know<br>4 = Mostly false<br>5 = Definitely false<br>6 = Not applicable	Yes<br>Recode to<br>1 = 100<br>2 = 75<br>3 = 50<br>4 = 25<br>5 = 0<br>6 = Missing<br><br>Pre: M=52.5316 SE=3.29728 SD=29.30688 Min=0 Max=100 N=79<br>Post: M=57.2917 SE=3.71487

			SD=31.52171 Min=0 Max=100 N=72<br>Follow: M=60.5634 SE=3.27565 SD=27.60110 Min=0 Max=100 N=71

Composite Functional Health Scores			
Variable Names:<br>• **Pretest**<br>• **Posttest**<br>• **Follow-up**	Variable Label	Values	Recode?
**Phys_funct_pre**<br>**Phys_funct_post**<br>**Phys_funct_follow**	Physical Functioning	FH3_1 + FH3_2 + FH3_3 + FH3_4 + FH3_5 + FH3_6 + FH3_7 + FH3_8 + FH3_9 + FH3_10<br><br>Pretest:<br>M = 86.9198<br>Max = 100<br>Min = 35<br>SD = 16.67540<br>SE = 1.87613<br>N = 79<br><br>Post-test:<br>M = 87.4074<br>Max = 100<br>Min = 10<br>SD = 17.86510<br>SE = 2.10542<br>N = 72<br><br>Follow-up:<br>M = 87.1349<br>Max = 100<br>Min = 5<br>SD = 20.42154<br>SE = 2.44084<br>N = 70	No (the variables we are summing need to be recoded, see above)
**Lim_phys_pre**<br>**Lim_phys_post**<br>**Lim_phys_follow**	Role limitations due to physical health	FH4_1 + FH4_2 + FH4_3 + FH4_4<br><br>Pretest:<br>M = 69.5148<br>Max = 100<br>Min = 0<br>SD = 33.75497<br>SE = 3.79773<br>N = 79<br><br>Post-test:	No

		M = 77.4648<br>Max = 100<br>Min = 0<br>SD = 35.89542<br>SE = 4.26000<br>N = 71<br><br>Follow-up:<br>M = 81.7857<br>Max = 100<br>Min = 10<br>SD = 34.81874<br>SE = 4.16164<br>N = 70	
**Lim_Emot_pre**<br>**Lim_Emot_post**<br>**Lim_Emot_follow**	Role limitations due to emotional problems	FH5_1 + FH5_2 + FH5_3<br><br>Pretest:<br>M = 59.0717<br>Max = 100<br>Min = 0<br>SD = 42.93352<br>SE = 4.83040<br>N = 79<br><br>Follow-up<br>M = 74.6479<br>Max = 100<br>Min = 0<br>SD = 37.99114<br>SE = 4.50872<br>N = 71	No
**Fatigue_Ener_pre**<br>**Fatigue_Ener_post**<br>**Fatigue_Ener_follow**	Energy/fatigue	FH9_1 + FH9_5 + FH9_7 + FH9_9<br><br>Pretest:<br>M = 45.7292<br>Max = 93.33<br>Min = 0<br>SD = 19.64675<br>SE = 2.19657<br>N = 80<br><br>Post-test:<br>M = 53.3333<br>Max = 90<br>Min = 15<br>SD = 18.03752<br>SE = 2.12574<br>N = 72<br><br>Follow-up:<br>M = 52.7230<br>Max = 95	No

		Min = 0<br>SD = 21.17995<br>SE = 2.51360<br>N = 71	
**Emot_well_pre**<br>**Emot_well_post**<br>**Emot_well_follow**	Emotional well-being	FH9_2 + FH9_3 + FH9_4 + FH9_6 + FH9_8<br><br>Pretest:<br>M = 65.5208<br>Max = 100<br>Min = 16<br>SD = 16.86797<br>SE = 1.88590<br>N = 80<br><br>Post-test:<br>M = 68.1528<br>Max = 96<br>Min = 16<br>SD = 15.74513<br>SE = 1.85558<br>N = 72<br><br>Follow-up:<br>M = 67.4178<br>Max = 98<br>Min = 20<br>SD = 17.66961<br>SE = 2.09700<br>N = 71	No
**Soc_funct_pre**<br>**Soc_funct_post**<br>**Soc_funct_follow**	Social functioning	FH6 + FH10<br><br>Pretest:<br>M = 70.7278<br>Max = 100<br>Min = 12.50<br>SD = 25.38849<br>SE = 2.85643<br>N = 79<br><br>Post-test:<br>M = 78.6972<br>Max = 100<br>Min = 12.50<br>SD = 23.08702<br>SE = 2.7399<br>N = 71<br><br>Follow-up:<br>M = 77.3551<br>Max = 100<br>Min = 0<br>SD = 23.60787	No

		SE = 2.84205<br>N = 69	
**Pain_pre**<br>**Pain_post**<br>**Pain_follow**	Pain	FH7 + FH8<br><br>Pretest:<br>M = 75.9063<br>Max = 87.50<br>Min = 12.50<br>SD = 19.43117<br>SE = 2.17247<br>N = 80<br><br>Post-test:<br>M = 75.7746<br>Max = 100<br>Min = 22.50<br>SD = 22.19070<br>SE = 2.63355<br>N = 71<br><br>Follow-up:<br>M = 75.3623<br>Max = 100<br>Min = 0<br>SD = 24.07202<br>SE = 2.89793<br>N = 69	No
**Gen_health_pre**<br>**Gen_health_post**<br>**Gen_health_follow**	General Health	FH1 + FH11_1 + FH11_2 + FH11_3 + FH11_4<br><br>Pretest:<br>M = 61.1250<br>Max = 100<br>Min = 15<br>SD = 21.22774<br>SE = 2.37333<br>N = 80<br><br>Post-test:<br>M = 64.0354<br>Max = 100<br>Min = 10<br>SD = 21.31355<br>SE = 2.49456<br>N = 73<br><br>Follow-up:<br>M = 64.0278<br>Max = 100<br>Min = 10<br>SD = 20/85562<br>SE = 2.45786<br>N = 72	No

Health Status			
Variable Name	Variable Label	Values	Recode?
**HS_1**	Have you recently... - Lost much sleep over worry?	1 = Not at all<br>2 = No more than usual<br>3 = More than usual<br>4 = Much more than usual<br>6 = Not applicable	Yes<br>Reverse Score/<br>Recode 6 = Missing<br><br>Pre: M=2.8205<br>SE=0.09952<br>SD=0.87895 Min=1<br>Max=4 N=78
**HS_2**	Have you recently... - Felt constantly under strain?	1 = Not at all<br>2 = No more than usual<br>3 = More than usual<br>4 = Much more than usual<br>6 = Not applicable	Yes<br>Reverse Score<br>Recode 6 = Missing<br><br>Pre: M=2.6154<br>SE=0.09523<br>SD=0.84101 Min=1<br>Max=4 N=78
**HS_3**	Have you recently... - Felt you couldn't overcome your difficulties?	1 = Not at all<br>2 = No more than usual<br>3 = More than usual<br>4 = Much more than usual<br>6 = Not applicable	Yes<br>Reverse Score<br>Recode 6 = Missing<br><br>Pre: M=3.1013<br>SE=0.09801<br>SD=0.87112 Min=1<br>Max=4 N=79
**HS_4**	Have you recently... - Been feeling unhappy or depressed?	1 = Not at all<br>2 = No more than usual<br>3 = More than usual<br>4 = Much more than usual<br>6 = Not applicable<br><br>Pre: M=2.7975<br>SE=0.09427<br>SD=0.83788 Min=1<br>Max=4 N=79	Yes<br>Reverse Score<br>Recode 6 = Missing<br><br>Pre: M=2.7975<br>SE=0.09427<br>SD=0.83788 Min=1<br>Max=4 N=79
**HS_5**	Have you recently... - Been losing confidence in yourself?	1 = Not at all<br>2 = No more than usual<br>3 = More than usual<br>4 = Much more than usual<br>6 = Not applicable	Yes<br>Reverse Score<br>Recode 6 = Missing<br><br>Pre: M=3.0633<br>SE=0.09069<br>SD=0.80609 Min=1<br>Max=4 N=79

**HS_6**	Have you recently... - Been thinking of yourself as a worthless person?	1 = Not at all<br>2 = No more than usual<br>3 = More than usual<br>4 = Much more than usual<br>6 = Not applicable	Yes<br>Reverse Score<br>Recode 6 = Missing<br><br>Pre: M=3.4177<br>SE=0.08937<br>SD=0.79433 Min=1<br>Max=4 N=77
**HS2_1**	Have you recently... - Felt you were playing a useful part in things?	1 = More than usual<br>2 = Same as usual<br>3 = Less than usual<br>4 = Much less than usual<br>5 = Not applicable	No<br>Recode 5 = Missing<br><br>Pre: M=2.3247<br>SE=0.08758<br>SD=0.76848 Min=1<br>Max=4 N=77
**HS2_2**	Have you recently... - Felt capable about making decisions about things?	1 = More than usual<br>2 = Same as usual<br>3 = Less than usual<br>4 = Much less than usual<br>5 = Not applicable	No<br>Recode 5 = Missing<br><br>Pre: M=2.0128<br>SE=0.05911<br>SD=0.52207 Min=1<br>Max=3 N=78
**HS2_3**	Have you recently... - Been able to enjoy your normal day-to-day activities?	1 = More than usual<br>2 = Same as usual<br>3 = Less than usual<br>4 = Much less than usual<br>5 = Not applicable	No<br>Recode 5 = Missing<br><br>Pre: M=2.3797<br>SE=0.07494<br>SD=0.66610 Min=1<br>Max=4 N=79
**HS2_4**	Have you recently... - Been able to face up to your problems?	1 = More than usual<br>2 = Same as usual<br>3 = Less than usual<br>4 = Much less than usual<br>5 = Not applicable	No<br>Recode 5 = Missing<br><br>Pre: M=2.1538<br>SE=0.07086<br>SD=0.62579 Min=1<br>Max=4 N=78
**HS2_5**	Have you recently... - Been feeling reasonably happy, all things considered?	1 = More than usual<br>2 = Same as usual<br>3 = Less than usual<br>4 = Much less than usual<br>5 = Not applicable	No<br>Recode 5 = Missing<br><br>Pre: M=2.3038<br>SE=0.07718<br>SD=0.68602 Min=1<br>Max=4 N=79
**HS3_1**	Have you recently... - Been able to concentrate on what you're doing?	1 = Better than usual<br>2 = Same as usual<br>3 = Less than usual<br>4 = Much less than usual	No<br>Recode 5 = Missing<br><br>Pre: M=2.3038<br>SE=0.06826

		5 = Not applicable	SD=0.60668 Min=1 Max=4 N=79

Composite GHQ Health States			
**GHQ_Comp_pre**	Composite General Health Score<br><br>Higher numbers indicating worse conditions	HS_1 + HS_2 + HS_3 + HS_4 + HS_5 + HS_6 + HS2_1 + HS2_2 + HS2_3 + HS2_4 + HS2_5 + HS3_1<br><br>M = 31.1139<br>Max = 39<br>Min = 21<br>SD = 3.23438<br>SE = 0.36390<br>N = 79	No

Physical Activity Questionnaire			
Variable Names:<br>• **Pretest**<br>• **Posttest**<br>• **Follow-up**	Variable Label	Values	Recode?
**PA2**<br>**Q82_post**<br>**Q82_follow**	During the last 7 days, on how many days did you do vigorous physical activities like heavy lifting, digging, aerobics, or fast bicycling? - Selected Choice	1 = Days per week<br>2 = No vigorous physical activities<br>4 = Not applicable	4 = 0<br>SYSMIS = 0
**PA2_1_TEXT**<br>**Q82_1_TEXT_post**<br>**Q82_1_TEXT_follow**	During the last 7 days, on how many days did you do vigorous physical activities like heavy lifting, digging, aerobics, or fast bicycling? - Days per week - Text	Manual Entry<br>Range: 0 – 7	SYSMIS = 0<br><br>Pre: M=0.8500 SE=0.16792 SD=1.50190 Min=0 Max=7 N=80<br>Post: M=1.6750 SE=0.22274 SD=1.99223 Min=0 Max=7 N=80<br>Follow: M=1.2000 SE=0.19761 SD=1.76750 Min=0 Max=7 N=80
**PA3**<br>**Q88_post**<br>**Q88_follow**	How much time did you usually spend doing vigorous physical activities on one of those days?	1 = Hours per day<br>2 = Minutes per day<br>4 = Don’t know/Not sure<br>5 = Not applicable	5 = 0<br>SYSMIS = 0

	If less than an hour per day, just fill out minutes per day. - Selected Choice		
**PA3_1_TEXT**<br>**Q88_1_TEXT_post**<br>**Q88_1_TEXT_follow**	How much time did you usually spend doing vigorous physical activities on one of those days?<br><br>If less than an hour per day, just fill out minutes per day. - Hours per day - Text	Manual Entry<br>Range: 0 – 6	SYSMIS = 0<br><br>Pre: M=0.2250 SE=0.08147 SD=0.72871 Min=0 Max=5 N=80<br>Post: M=0.3625 SE=0.09113 SD=0.81511 Min=0 Max=4 N=80<br>Follow: M=0.2375 SE=0.09113 SD=0.81511 Min=0 Max=6 N=80
**PA3_2_TEXT**<br>**Q88_2_TEXT_post**<br>**Q88_2_TEXT_follow**	How much time did you usually spend doing vigorous physical activities on one of those days?<br><br>If less than an hour per day, just fill out minutes per day. - Minutes per day - Text	Manual Entry<br>Range: 1 – 90	SYSMIS = 0<br><br>Pre: M=6.6125 SE=1.66958 SD=14.93318 Min=0 Max=60 N=80<br>Post: M=7.5375 SE=1.71525 SD=15.34163 Min=0 Max=60 N=80<br>Follow: M=7.4875 SE=1.96271 SD=17.55497 Min=0 Max=90 N=80
**PA5**<br>**Q80_post**<br>**Q80_follow**	During the last 7 days, on how many days did you do moderate physical activities like carrying light loads, bicycling at a regular pace, or doubles tennis? Do not include walking. - Selected Choice	1 = Days per week<br>2 = No moderate physical activity<br>4 = Not applicable	4 = 0<br>SYSMIS = 0
**PA5_1_TEXT**<br>**Q80_1_TEXT_post**<br>**Q80_1_TEXT_follow**	During the last 7 days, on how many days did you do moderate physical activities like carrying light loads, bicycling at a regular pace, or doubles tennis? Do not include walking. - Days per week - Text	Manual Entry<br>Range: 0 – 7	SYSMIS = 0<br><br>Pre: M=1.6125 SE=0.20935 SD=1.87248 Min=0 Max=7 N=80<br>Post: M=2.5000 SE=0.26326

			SD=2.35463 Min=0 Max=7 N=80<br>Follow: M=2.4125 SE=0.28778 SD=2.57394 Min=0 Max=7 N=80
**PA6**<br>**Q86_post**<br>**Q86_follow**	How much time did you usually spend doing moderate physical activities on one of those days?<br><br>If less than an hour per day, just fill out minutes per day. - Selected Choice	1 = Hours per day<br>2 = Minutes per day<br>3 = Don't know/Not sure<br>4 = Not applicable	4 = 0<br>SYSMIS = 0
**PA6_1_TEXT**<br>**Q86_1_TEXT_post**<br>**Q86_1_TEXT_follow**	How much time did you usually spend doing moderate physical activities on one of those days?<br><br>If less than an hour per day, just fill out minutes per day. - Hours per day - Text	Manual Entry<br>Range: 1 – 8	SYSMIS = 0<br><br>Pre: M=0.4125 SE=0.11208 SD=1.00245 Min=0 Max=7 N=80<br>Post: M=0.6875 SE=0.16554 SD=1.48062 Min=0 Max=8 N=80<br>Follow: M=0.6750 SE=0.17135 SD=1.53256 Min=0 Max=8 N=80
**PA6_2_TEXT**<br>**Q86_2_TEXT_post**<br>**Q86_2_TEXT_follow**	How much time did you usually spend doing moderate physical activities on one of those days?<br><br>If less than an hour per day, just fill out minutes per day. - Minutes per day - Text	Manual Entry<br>Range: 1 – 120	SYSMIS = 0<br><br>Pre: M=8.5875 SE=1.71033 SD=15.29763 Min=0 Max=60 N=80<br>Post: M=8.8125 SE=2.12959 SD=19.04764 Min=0 Max=120 N=80<br>Follow: M=9.7500 SE=2.30900 SD=20.65234 Min=0 Max=100 N=80
**PA8**<br>**Q84_post**<br>**Q84_follow**	During the last 7 days, on how many days did you walk for at least 10 minutes at a time? - Selected Choice	1 = Days per week<br>2 = No walking<br>4 = Not applicable	4 = 0<br>SYSMIS = 0

**PA8_1_TEXT**<br>**Q84_1_TEXT_post**<br>**Q84_1_TEXT_follow**	During the last 7 days, on how many days did you walk for at least 10 minutes at a time? - Days per week - Text	Manual Entry<br>Range: 1 – 7	SYSMIS = 0<br><br>Pre: M=3.8375 SE=0.26710 SD=2.38902 Min=0 Max=7 N=80<br>Post: M=3.8375 SE=0.29416 SD=2.63109 Min=0 Max=7 N=80<br>Follow: M=4.2250 SE=0.29499 SD=2.63845 Min=0 Max=7 N=80
**PA9**<br>**Q90_post**<br>**Q90_follow**	How much time did you usually spend walking on one of those days?<br><br>If less than an hour per day, just fill out minutes per day. - Selected Choice	1 = Hours per day<br>2 = Minutes per day<br>3 = Don't know/Not sure<br>4 = Not applicable	4 = 0<br>SYSMIS = 0
**PA9_1_TEXT**<br>**Q90_1_TEXT_post**<br>**Q90_1_TEXT_follow**	How much time did you usually spend walking on one of those days?<br><br>If less than an hour per day, just fill out minutes per day. - Hours per day - Text	Manual Entry<br>Range: 1 – 8	SYSMIS = 0<br><br>Pre: M=0.6500 SE=0.16119 SD=1.44169 Min=0 Max=8 N=80<br>Post: M=0.7750 SE=0.17964 SD=1.60675 Min=0 Max=8 N=80<br>Follow: M=0.6125 SE=0.13721 SD=1.22726 Min=0 Max=8 N=80
**PA9_2_TEXT**<br>**Q90_2_TEXT_post**<br>**Q90_2_TEXT_follow**	How much time did you usually spend walking on one of those days?<br><br>If less than an hour per day, just fill out minutes per day. - Minutes per day - Text	Manual Entry<br>Range: 10 – 120	SYSMIS = 0<br><br>Pre: M=14.9625 SE=2.23744 SD=20.01230 Min=0 Max=120 N=80<br>Post: M=10.1500 SE=1.61736 SD=14.46611 Min=0 Max=45 N=80<br>Follow: M=14.0875 SE=2.13183 SD=19.06763 Min=0 Max=85 N=80

**PA11**<br>**Q78_post**<br>**Q78_follow**	During the last 7 days, how much time did you spend sitting on a week day?<br><br>If less than an hour per day, just fill out minutes per day. - Selected Choice	1 = Hours per day<br>2 = Minutes per day<br>3 = Don't know/Not sure<br>4 = Not applicable	4 = 0<br>SYSMIS = 0
**PA11_1_TEXT**<br>**Q78_1_TEXT_post**<br>**Q78_1_TEXT_follow**	During the last 7 days, how much time did you spend sitting on a week day?<br><br>If less than an hour per day, just fill out minutes per day. - Hours per day - Text	Manual Entry<br>Range: 1 – 24	SYSMIS = 0<br><br>Pre: M=6.4375 SE=0.48026 SD=4.29555 Min=0 Max=18 N=80<br>Post: M=4.5500 SE=0.41335 SD=3.69707 Min=0 Max=16 N=80<br>Follow: M=4.7625 SE=0.46020 SD=4.11617 Min=0 Max=20 N=80
**PA11_2_TEXT**<br>**Q78_2_TEXT_post**<br>**Q78_2_TEXT_follow**	During the last 7 days, how much time did you spend sitting on a week day?<br><br>If less than an hour per day, just fill out minutes per day. - Minutes per day - Text	Manual Entry<br>Range: 20 – 400	SYSMIS = 0<br><br>Pre: M=3.7500 SE=3.75000 SD=33.54102 Min=0 Max=300 N=80<br>Post: M=0.7500 SE=0.52696 SD=4.71330 Min=0 Max=30 N=80<br>Follow: M=8.0625 SE=5.47325 SD=48.95421 Min=0 Max=400 N=80

**Composite Physical Activity**			
**Tot_MET_pre**<br>**Tot_MET_post**<br>**Tot_MET_follow**	Total physical activity MET-minutes/week	Vig_MET + Mod_MET + Walk_MET<br><br>Pretest<br>M = 1999.2056<br>Max = 20292<br>Min = 63.10<br>SD = 3360.27405<br>SE = 398.79116<br>N = 71<br><br>Post-test<br>M = 3202.5547	No

		Max = 14895<br>Min = 73<br>SD = 3750.95932<br>SE = 468.86991<br>N = 64<br><br>Follow-up<br>M = 3326.2062<br>Max = 30186<br>Min = 105<br>SD = 5133.78966<br>SE = 636.76824<br>N = 65	

Self-Control			
Variable Names:<br>• **Pretest**<br>• **Posttest**<br>• **Follow-up**	Variable Label	Values	Higher values = higher self-control
**Self_Control_1**<br>**Q22_1**<br>**Q21_1**	For each of the following statements please indicate how much each of the following statements reflects how you typically are. - I am good at resisting temptation.	1 = A great deal<br>2 = A lot<br>3 = A moderate amount<br>4 = A little<br>5 = None at all<br>6 = Not applicable	Yes<br>Reverse Coded<br><br>Recode 6 = Missing<br><br>Pre: M=2.6375 SE=0.11144 SD=0.99675 Min=1 Max=5 N=80<br>Post: M=3.0139 SE=0.12894 SD=1.09407 Min=1 Max=5 N=72<br>Follow: M=2.9857 SE=0.12949 SD=1.08338 Min=1 Max=5 N=70
**Self_Control_2**<br>**Q22_2**<br>**Q21_2**	For each of the following statements please indicate how much each of the following statements reflects how you typically are. - I have a hard time breaking bad habits.	1 = A great deal<br>2 = A lot<br>3 = A moderate amount<br>4 = A little<br>5 = None at all<br>6 = Not applicable	Recode 6 = Missing<br><br>Pre: M=2.7875 SE=0.13394 SD=1.19803 Min=1 Max=5 N=80<br>Post: M=3.2917 SE=0.14047 SD=1.19196 Min=1 Max=5 N=72<br>Follow: M=3.3478 SE=0.14128 SD=1.17356 Min=1 Max=5 N=69

**Self_Control_3**<br>**Q22_3**<br>**Q21_3**	For each of the following statements please indicate how much each of the following statements reflects how you typically are. - I am lazy.	1 = A great deal<br>2 = A lot<br>3 = A moderate amount<br>4 = A little<br>5 = None at all<br>6 = Not applicable	Recode 6 = Missing<br><br>Pre: M=3.8861 SE=0.12349 SD=1.09764 Min=1 Max=5 N=79<br>Post: M=4.0714 SE=0.11921 SD=0.99741 Min=1 Max=5 N=70<br>Follow: M=4.0286 SE=0.13339 SD=1.11604 Min=1 Max=5 N=70
**Self_Control_4**<br>**Q22_4**<br>**Q21_4**	For each of the following statements please indicate how much each of the following statements reflects how you typically are. - I say inappropriate things.	1 = A great deal<br>2 = A lot<br>3 = A moderate amount<br>4 = A little<br>5 = None at all<br>6 = Not applicable	Recode 6 = Missing<br><br>Pre: M=4.0000 SE=0.12643 SD=1.11658 Min=1 Max=5 N=78<br>Post: M=4.1268 SE=0.11856 SD=0.99899 Min=1 Max=5 N=71<br>Follow: M=4.2286 SE=0.12068 SD=1.00968 Min=1 Max=5 N=70
**Self_Control_5**<br>**Q22_5**<br>**Q21_5**	For each of the following statements please indicate how much each of the following statements reflects how you typically are. - I do certain things that are bad for me, if they are fun.	1 = A great deal<br>2 = A lot<br>3 = A moderate amount<br>4 = A little<br>5 = None at all<br>6 = Not applicable	Recode 6 = Missing<br><br>Pre: M=3.9000 SE=0.10464 SD=0.93592 Min=1 Max=5 N=80<br>Post: M=3.9429 SE=0.09074 SD=0.75921 Min=2 Max=5 N=70<br>Follow: M=4.0294 SE=0.11081 SD=0.91375 Min=1 Max=5 N=68
**Self_Control_6**<br>**Q22_6**<br>**Q21_6**	For each of the following statements please indicate how much each of the following statements reflects how you typically are. - I refuse things that are bad for me.	1 = A great deal<br>2 = A lot<br>3 = A moderate amount<br>4 = A little<br>5 = None at all<br>6 = Not applicable	Yes<br>Reverse Coded<br><br>Recode 6 = Missing<br><br>Pre: M=2.9494 SE=0.11380 SD=1.01146 Min=1 Max=5 N=79<br>Post: M=3.0278 SE=0.13265

			SD=1.12553 Min=1<br>Max=5 N=72<br>Follow: M=3.2714<br>SE=0.13017<br>SD=1.08910 Min=1<br>Max=5 N=70
**Self_Control_7**<br>**Q22_7**<br>**Q21_7**	For each of the following statements please indicate how much each of the following statements reflects how you typically are. - I wish I had more self-discipline.	1 = A great deal<br>2 = A lot<br>3 = A moderate amount<br>4 = A little<br>5 = None at all<br>6 = Not applicable	Recode 6 = Missing<br><br>Pre: M=2.6076<br>SE=0.16256<br>SD=1.44486 Min=1<br>Max=5 N=79<br>Post: M=2.9028<br>SE=0.16089<br>SD=1.36516 Min=1<br>Max=5 N=72<br>Follow: M=2.9714<br>SE=0.16148<br>SD=1.35102 Min=1<br>Max=5 N=70
**Self_Control_8**<br>**Q22_8**<br>**Q21_8**	For each of the following statements please indicate how much each of the following statements reflects how you typically are. - People would say that I have iron self-discipline.	1 = A great deal<br>2 = A lot<br>3 = A moderate amount<br>4 = A little<br>5 = None at all<br>6 = Not applicable	Yes<br>Reverse Coded<br><br>Recode 6 = Missing<br><br>Pre: M=2.3544<br>SE=0.13120<br>SD=1.16617 Min=1<br>Max=5 N=79<br>Post: M=2.8571<br>SE=0.14140<br>SD=1.18304 Min=1<br>Max=5 N=70<br>Follow: M=2.6232<br>SE=0.14319<br>SD=1.18943 Min=1<br>Max=5 N=69
**Self_Control_9**<br>**Q22_9**<br>**Q21_9**	For each of the following statements please indicate how much each of the following statements reflects how you typically are. - Pleasure and fun sometimes keep me from getting work done.	1 = A great deal<br>2 = A lot<br>3 = A moderate amount<br>4 = A little<br>5 = None at all<br>6 = Not applicable	Recode 6 = Missing<br><br>Pre: M=3.8205<br>SE=0.14339<br>SD=1.26638 Min=1<br>Max=5 N=78<br>Post: M=3.9014<br>SE=0.12391<br>SD=1.04410 Min=1<br>Max=5 N=71<br>Follow: M=4.0435<br>SE=0.13128<br>SD=1.09053 Min=1<br>Max=5 N=69

**Self_Control_10**<br>**Q22_10**<br>**Q21_10**	For each of the following statements please indicate how much each of the following statements reflects how you typically are. - I have trouble concentrating.	1 = A great deal<br>2 = A lot<br>3 = A moderate amount<br>4 = A little<br>5 = None at all<br>6 = Not applicable	Recode 6 = Missing<br><br>Pre: M=3.5897 SE=0.13950 SD=1.23200 Min=1 Max=5 N=78<br>Post: M=3.7183 SE=0.13779 SD=1.16107 Min=1 Max=5 N=71<br>Follow: M=3.7714 SE=0.13976 SD=1.16931 Min=1 Max=5 N=70
**Self_Control_11**<br>**Q22_11**<br>**Q21_11**	For each of the following statements please indicate how much each of the following statements reflects how you typically are. - I am able to work effectively toward long-term goals.	1 = A great deal<br>2 = A lot<br>3 = A moderate amount<br>4 = A little<br>5 = None at all<br>6 = Not applicable	Yes<br>Reverse Coded<br><br>Recode 6 = Missing<br><br>Pre: M=3.2375 SE=0.12227 SD=1.09364 Min=1 Max=5 N=80<br>Post: M=3.5139 SE=0.11532 SD=0.97855 Min=2 Max=5 N=72<br>Follow: M=3.5286 SE=0.13608 SD=1.13854 Min=1 Max=5 N=70
**Self_Control_12**<br>**Q22_12**<br>**Q21_12**	For each of the following statements please indicate how much each of the following statements reflects how you typically are. - Sometimes I can't stop myself from doing something, even if I know it is wrong.	1 = A great deal<br>2 = A lot<br>3 = A moderate amount<br>4 = A little<br>5 = None at all<br>6 = Not applicable	Recode 6 = Missing<br><br>Pre: M=3.9737 SE=0.11990 SD=1.04529 Min=1 Max=5 N=76<br>Post: M=4.0417 SE=0.11090 SD=0.94104 Min=2 Max=5 N=72<br>Follow: M=4.1449 SE=0.10783 SD=0.89567 Min=1 Max=5 N=69
**Self_Control_13**<br>**Q22_13**<br>**Q21_13**	For each of the following statements please indicate how much each of the following statements reflects how you typically are. - I often act without thinking	1 = A great deal<br>2 = A lot<br>3 = A moderate amount<br>4 = A little<br>5 = None at all<br>6 = Not applicable	Recode 6 = Missing<br><br>Pre: M=4.0759 SE=0.12452 SD=1.10676 Min=1 Max=5 N=79<br>Post: M=4.1571 SE=0.10889

	through all the alternatives.		SD=0.91105 Min=1 Max=5 N=70<br>Follow: M=4.2319 SE=0.11886 SD=0.98735 Min=1 Max=5 N=69

**Composite Self-Control**			
Variable Names:<br>• **Pretest**<br>• **Posttest**<br>• **Follow-up**	Variable Label	Values	Recode
**Self_Cont_Comp_pre**<br>**Self_Cont_Comp_post**<br>**Self_Cont_Comp_follow**	Composite Self-Control	Self_Control_1 +<br>Self_Control_2 +<br>Self_Control_3 +<br>Self_Control_4 +<br>Self_Control_5 +<br>Self_Control_6 +<br>Self_Control_7 +<br>Self_Control_8 +<br>Self_Control_9 +<br>Self_Control_10 +<br>Self_Control_11 +<br>Self_Control_12 +<br>Self_Control_13<br><br>Pretest<br>M = 3.3660<br>Max = 4.77<br>Min = 1.38<br>SD = 0.67112<br>SE = 0.07503<br>N = 80<br><br>Post-test<br>M = 3.5787<br>Max = 5<br>Min = 2.08<br>SD = 0.69573<br>SE = 0.08199<br>N = 72<br><br>Follow-up<br>M = 3.6300<br>Max = 4.92<br>Min = 1.62<br>SD = 0.69825<br>SE = 0.08346<br>N = 70	No (individual component variables need recoding, see above)

**Big 5 Inventory**			
Variable Names:	Variable Label	Values	Recode?

• **Pretest**<br>• **Posttest**<br>• **Follow-up**			
**B5___Part_1_1**<br>**BF___Part_1_1**<br>**Q22_1_follow**	Here are a number of characteristics that may or may not apply to you. For example, do you agree that you are someone who likes to spend time with others?<br><br>For each of the following statements please indicate how much you agree/disagree that each characteristic applies to you.<br><br>I am someone who... - Is outgoing, sociable.	1 = Disagree strongly<br>2 = Disagree a little<br>3 = Neutral; no opinion<br>4 = Agree a little<br>5 = Agree strongly<br>6 = Not applicable<br><br>Pre: M= SE= SD= Min= Max= N=<br>Post: M= SE= SD= Min= Max= N=<br>Follow: M= SE= SD= Min= Max= N=	Recode 6 = Missing
**B5___Part_1_2**<br>**BF___Part_1_2**<br>**Q22_2_follow**	Here are a number of characteristics that may or may not apply to you. For example, do you agree that you are someone who likes to spend time with others?<br><br>For each of the following statements please indicate how much you agree/disagree that each characteristic applies to you.<br><br>I am someone who... - Is compassionate, has a soft heart.	1 = Disagree strongly<br>2 = Disagree a little<br>3 = Neutral; no opinion<br>4 = Agree a little<br>5 = Agree strongly<br>6 = Not applicable<br><br>Pre: M= SE= SD= Min= Max= N=<br>Post: M= SE= SD= Min= Max= N=<br>Follow: M= SE= SD= Min= Max= N=	Recode 6 = Missing
**B5___Part_1_3**<br>**BF___Part_1_3**<br>**Q22_3_follow**	Here are a number of characteristics that may or may not apply to you. For example, do you agree that you are someone who likes to spend time with others?<br><br>For each of the following statements please indicate how much you agree/disagree that each characteristic applies to you.	1 = Disagree strongly<br>2 = Disagree a little<br>3 = Neutral; no opinion<br>4 = Agree a little<br>5 = Agree strongly<br>6 = Not applicable<br><br>Pre: M= SE= SD= Min= Max= N=<br>Post: M= SE= SD= Min= Max= N=<br>Follow: M= SE= SD= Min= Max= N=	Yes (Reverse)<br><br>Recode 6 = Missing

	I am someone who... - Tends to be disorganized.		
**B5___Part_1_4**<br>**BF___Part_1_4**<br>**Q22_4_follow**	Here are a number of characteristics that may or may not apply to you. For example, do you agree that you are someone who likes to spend time with others?<br><br>For each of the following statements please indicate how much you agree/disagree that each characteristic applies to you.<br><br>I am someone who... - Is relaxed, handles stress well.	1 = Disagree strongly<br>2 = Disagree a little<br>3 = Neutral; no opinion<br>4 = Agree a little<br>5 = Agree strongly<br>6 = Not applicable<br><br>Pre: M= SE= SD= Min= Max= N=<br>Post: M= SE= SD= Min= Max= N=<br>Follow: M= SE= SD= Min= Max= N=	Yes (Reverse)<br><br>Recode 6 = Missing
**B5___Part_1_5**<br>**BF___Part_1_5**<br>**Q22_5_follow**	Here are a number of characteristics that may or may not apply to you. For example, do you agree that you are someone who likes to spend time with others?<br><br>For each of the following statements please indicate how much you agree/disagree that each characteristic applies to you.<br><br>I am someone who... - Has few artistic interests.	1 = Disagree strongly<br>2 = Disagree a little<br>3 = Neutral; no opinion<br>4 = Agree a little<br>5 = Agree strongly<br>6 = Not applicable<br><br>Pre: M= SE= SD= Min= Max= N=<br>Post: M= SE= SD= Min= Max= N=<br>Follow: M= SE= SD= Min= Max= N=	Yes (Reverse)<br><br>Recode 6 = Missing
**B5___Part_1_6**<br>**BF___Part_1_6**<br>**Q22_6_follow**	Here are a number of characteristics that may or may not apply to you. For example, do you agree that you are someone who likes to spend time with others?<br><br>For each of the following statements please indicate how much you agree/disagree that each characteristic applies to you.<br><br>I am someone who... - Has an assertive personality.	1 = Disagree strongly<br>2 = Disagree a little<br>3 = Neutral; no opinion<br>4 = Agree a little<br>5 = Agree strongly<br>6 = Not applicable<br><br>Pre: M= SE= SD= Min= Max= N=<br>Post: M= SE= SD= Min= Max= N=<br>Follow: M= SE= SD= Min= Max= N=	Recode 6 = Missing

**B5___Part_1_7**<br>**BF___Part_1_7**<br>**Q22_7_follow**	Here are a number of characteristics that may or may not apply to you. For example, do you agree that you are someone who likes to spend time with others?<br><br>For each of the following statements please indicate how much you agree/disagree that each characteristic applies to you.<br><br>I am someone who... - Is respectful, treats others with respect.	1 = Disagree strongly<br>2 = Disagree a little<br>3 = Neutral; no opinion<br>4 = Agree a little<br>5 = Agree strongly<br>6 = Not applicable<br><br>Pre: M= SE= SD= Min= Max= N=<br>Post: M= SE= SD= Min= Max= N=<br>Follow: M= SE= SD= Min= Max= N=	Recode 6 = Missing
**B5___Part_1_8**<br>**BF___Part_1_8**<br>**Q22_8_follow**	Here are a number of characteristics that may or may not apply to you. For example, do you agree that you are someone who likes to spend time with others?<br><br>For each of the following statements please indicate how much you agree/disagree that each characteristic applies to you.<br><br>I am someone who... - Tends to be lazy.	1 = Disagree strongly<br>2 = Disagree a little<br>3 = Neutral; no opinion<br>4 = Agree a little<br>5 = Agree strongly<br>6 = Not applicable<br><br>Pre: M= SE= SD= Min= Max= N=<br>Post: M= SE= SD= Min= Max= N=<br>Follow: M= SE= SD= Min= Max= N=	Yes (Reverse)<br><br>Recode 6 = Missing
**B5___Part_1_9**<br>**BF___Part_1_9**<br>**Q22_9_follow**	Here are a number of characteristics that may or may not apply to you. For example, do you agree that you are someone who likes to spend time with others?<br><br>For each of the following statements please indicate how much you agree/disagree that each characteristic applies to you.<br><br>I am someone who... - Stays optimistic after experiencing a setback.	1 = Disagree strongly<br>2 = Disagree a little<br>3 = Neutral; no opinion<br>4 = Agree a little<br>5 = Agree strongly<br>6 = Not applicable<br><br>Pre: M= SE= SD= Min= Max= N=<br>Post: M= SE= SD= Min= Max= N=<br>Follow: M= SE= SD= Min= Max= N=	Yes (Reverse)<br><br>Recode 6 = Missing
**B5___Part_1_10**<br>**BF___Part_1_10**<br>**Q22_10_follow**	Here are a number of characteristics that may or may not apply to you.	1 = Disagree strongly<br>2 = Disagree a little	Recode 6 = Missing

	For example, do you agree that you are someone who likes to spend time with others?<br><br>For each of the following statements please indicate how much you agree/disagree that each characteristic applies to you.<br><br>I am someone who... - Stays optimistic after experiencing a setback.	3 = Neutral; no opinion<br>4 = Agree a little<br>5 = Agree strongly<br>6 = Not applicable<br><br>Pre: M= SE= SD= Min= Max= N=<br>Post: M= SE= SD= Min= Max= N=<br>Follow: M= SE= SD= Min= Max= N=	
**B5___Part_1_11**<br>**BF___Part_1_11**<br>**Q22_11_follow**	Here are a number of characteristics that may or may not apply to you. For example, do you agree that you are someone who likes to spend time with others?<br><br>For each of the following statements please indicate how much you agree/disagree that each characteristic applies to you.<br><br>I am someone who... - Stays optimistic after experiencing a setback.	1 = Disagree strongly<br>2 = Disagree a little<br>3 = Neutral; no opinion<br>4 = Agree a little<br>5 = Agree strongly<br>6 = Not applicable<br><br>Pre: M= SE= SD= Min= Max= N=<br>Post: M= SE= SD= Min= Max= N=<br>Follow: M= SE= SD= Min= Max= N=	Yes (Reverse)<br><br>Recode 6 = Missing
**B5___Part_1_12**<br>**BF___Part_1_12**<br>**Q22_12_follow**	Here are a number of characteristics that may or may not apply to you. For example, do you agree that you are someone who likes to spend time with others?<br><br>For each of the following statements please indicate how much you agree/disagree that each characteristic applies to you.<br><br>I am someone who... - Tends to find fault with others.	1 = Disagree strongly<br>2 = Disagree a little<br>3 = Neutral; no opinion<br>4 = Agree a little<br>5 = Agree strongly<br>6 = Not applicable<br><br>Pre: M= SE= SD= Min= Max= N=<br>Post: M= SE= SD= Min= Max= N=<br>Follow: M= SE= SD= Min= Max= N=	Yes (Reverse)<br><br>Recode 6 = Missing
**B5___Part_1_13**<br>**BF___Part_1_13**<br>**Q22_13_follow**	Here are a number of characteristics that may or may not apply to you. For example, do you agree that you are	1 = Disagree strongly<br>2 = Disagree a little<br>3 = Neutral; no opinion<br>4 = Agree a little	Recode 6 = Missing

	someone who likes to spend time with others?<br><br>For each of the following statements please indicate how much you agree/disagree that each characteristic applies to you.<br><br>I am someone who... - Is dependable, steady.	5 = Agree strongly<br>6 = Not applicable<br><br>Pre: M= SE= SD= Min= Max= N=<br>Post: M= SE= SD= Min= Max= N=<br>Follow: M= SE= SD= Min= Max= N=	
**B5___Part_1_14**<br>**BF___Part_1_14**<br>**Q22_14_follow**	Here are a number of characteristics that may or may not apply to you. For example, do you agree that you are someone who likes to spend time with others?<br><br>For each of the following statements please indicate how much you agree/disagree that each characteristic applies to you.<br><br>I am someone who... - Is moody, has up and down mood swings.	1 = Disagree strongly<br>2 = Disagree a little<br>3 = Neutral; no opinion<br>4 = Agree a little<br>5 = Agree strongly<br>6 = Not applicable<br><br>Pre: M= SE= SD= Min= Max= N=<br>Post: M= SE= SD= Min= Max= N=<br>Follow: M= SE= SD= Min= Max= N=	Recode 6 = Missing
**B5___Part_1_15**<br>**BF___Part_1_15**<br>**Q22_15_follow**	Here are a number of characteristics that may or may not apply to you. For example, do you agree that you are someone who likes to spend time with others?<br><br>For each of the following statements please indicate how much you agree/disagree that each characteristic applies to you.<br><br>I am someone who... - Is inventive, finds clever ways to do things.	1 = Disagree strongly<br>2 = Disagree a little<br>3 = Neutral; no opinion<br>4 = Agree a little<br>5 = Agree strongly<br>6 = Not applicable<br><br>Pre: M= SE= SD= Min= Max= N=<br>Post: M= SE= SD= Min= Max= N=<br>Follow: M= SE= SD= Min= Max= N=	Recode 6 = Missing
**B5___Part_1_16**<br>**BF___Part_1_16**<br>**Q22_16_follow**	Here are a number of characteristics that may or may not apply to you. For example, do you agree that you are someone who likes to spend time with others?	1 = Disagree strongly<br>2 = Disagree a little<br>3 = Neutral; no opinion<br>4 = Agree a little<br>5 = Agree strongly<br>6 = Not applicable	Yes (Reverse)<br><br>Recode 6 = Missing

	For each of the following statements please indicate how much you agree/disagree that each characteristic applies to you.<br><br>I am someone who... - Tends to be quiet.	Pre: M= SE= SD= Min= Max= N=<br>Post: M= SE= SD= Min= Max= N=<br>Follow: M= SE= SD= Min= Max= N=	
**B5___Part_1_17**<br>**BF___Part_1_17**<br>**Q22_17_follow**	Here are a number of characteristics that may or may not apply to you. For example, do you agree that you are someone who likes to spend time with others?<br><br>For each of the following statements please indicate how much you agree/disagree that each characteristic applies to you.<br><br>I am someone who... - Feels little sympathy for others.	1 = Disagree strongly<br>2 = Disagree a little<br>3 = Neutral; no opinion<br>4 = Agree a little<br>5 = Agree strongly<br>6 = Not applicable<br><br>Pre: M= SE= SD= Min= Max= N=<br>Post: M= SE= SD= Min= Max= N=<br>Follow: M= SE= SD= Min= Max= N=	Yes (Reverse)<br><br>Recode 6 = Missing
**B5___Part_1_18**<br>**BF___Part_1_18**<br>**Q22_18_follow**	Here are a number of characteristics that may or may not apply to you. For example, do you agree that you are someone who likes to spend time with others?<br><br>For each of the following statements please indicate how much you agree/disagree that each characteristic applies to you.<br><br>I am someone who... - Is systematic, likes to keep things in order.	1 = Disagree strongly<br>2 = Disagree a little<br>3 = Neutral; no opinion<br>4 = Agree a little<br>5 = Agree strongly<br>6 = Not applicable<br><br>Pre: M= SE= SD= Min= Max= N=<br>Post: M= SE= SD= Min= Max= N=<br>Follow: M= SE= SD= Min= Max= N=	Recode 6 = Missing
**B5___Part_1_19**<br>**BF___Part_1_19**<br>**Q22_19_follow**	Here are a number of characteristics that may or may not apply to you. For example, do you agree that you are someone who likes to spend time with others?<br><br>For each of the following statements please	1 = Disagree strongly<br>2 = Disagree a little<br>3 = Neutral; no opinion<br>4 = Agree a little<br>5 = Agree strongly<br>6 = Not applicable<br><br>Pre: M= SE= SD= Min= Max= N=	Recode 6 = Missing

	indicate how much you agree/disagree that each characteristic applies to you.<br><br>I am someone who... - Can be tense.	Post: M= SE= SD= Min= Max= N=<br>Follow: M= SE= SD= Min= Max= N=	
**B5___Part_1_20**<br>**BF___Part_1_20**<br>**Q22_20_follow**	Here are a number of characteristics that may or may not apply to you. For example, do you agree that you are someone who likes to spend time with others?<br><br>For each of the following statements please indicate how much you agree/disagree that each characteristic applies to you.<br><br>I am someone who... - Is fascinated by art, music, or literature.	1 = Disagree strongly<br>2 = Disagree a little<br>3 = Neutral; no opinion<br>4 = Agree a little<br>5 = Agree strongly<br>6 = Not applicable<br><br>Pre: M= SE= SD= Min= Max= N=<br>Post: M= SE= SD= Min= Max= N=<br>Follow: M= SE= SD= Min= Max= N=	Recode 6 = Missing
**B5___Part_2_1**<br>**BF___Part_2_1**<br>**Q23_1_follow**	Here are a number of characteristics that may or may not apply to you. For example, do you agree that you are someone who likes to spend time with others?<br><br>For each of the following statements please indicate how much you agree/disagree that each characteristic applies to you.<br><br>I am someone who... - Is dominant, acts as a leader.	1 = Disagree strongly<br>2 = Disagree a little<br>3 = Neutral; no opinion<br>4 = Agree a little<br>5 = Agree strongly<br>6 = Not applicable<br><br>Pre: M= SE= SD= Min= Max= N=<br>Post: M= SE= SD= Min= Max= N=<br>Follow: M= SE= SD= Min= Max= N=	Recode 6 = Missing
**B5___Part_2_2**<br>**BF___Part_2_2**<br>**Q23_2_follow**	Here are a number of characteristics that may or may not apply to you. For example, do you agree that you are someone who likes to spend time with others?<br><br>For each of the following statements please indicate how much you agree/disagree that each	1 = Disagree strongly<br>2 = Disagree a little<br>3 = Neutral; no opinion<br>4 = Agree a little<br>5 = Agree strongly<br>6 = Not applicable<br><br>Pre: M= SE= SD= Min= Max= N=<br>Post: M= SE= SD= Min= Max= N=	Yes (Reverse)<br><br>Recode 6 = Missing

	characteristic applies to you.<br><br>I am someone who... - Starts argument with others.	Follow: M= SE= SD= Min= Max= N=	
**B5___Part_2_3**<br>**BF___Part_2_3**<br>**Q23_3_follow**	Here are a number of characteristics that may or may not apply to you. For example, do you agree that you are someone who likes to spend time with others?<br><br>For each of the following statements please indicate how much you agree/disagree that each characteristic applies to you.<br><br>I am someone who... - Has difficulty getting started on tasks.	1 = Disagree strongly<br>2 = Disagree a little<br>3 = Neutral; no opinion<br>4 = Agree a little<br>5 = Agree strongly<br>6 = Not applicable<br><br>Pre: M= SE= SD= Min= Max= N=<br>Post: M= SE= SD= Min= Max= N=<br>Follow: M= SE= SD= Min= Max= N=	Yes (Reverse)<br><br>Recode 6 = Missing
**B5___Part_2_4**<br>**BF___Part_2_4**<br>**Q23_4_follow**	Here are a number of characteristics that may or may not apply to you. For example, do you agree that you are someone who likes to spend time with others?<br><br>For each of the following statements please indicate how much you agree/disagree that each characteristic applies to you.<br><br>I am someone who... - Feels secure, comfortable with self.	1 = Disagree strongly<br>2 = Disagree a little<br>3 = Neutral; no opinion<br>4 = Agree a little<br>5 = Agree strongly<br>6 = Not applicable<br><br>Pre: M= SE= SD= Min= Max= N=<br>Post: M= SE= SD= Min= Max= N=<br>Follow: M= SE= SD= Min= Max= N=	Yes (Reverse)<br><br>Recode 6 = Missing
**B5___Part_2_5**<br>**BF___Part_2_5**<br>**Q23_5_follow**	Here are a number of characteristics that may or may not apply to you. For example, do you agree that you are someone who likes to spend time with others?<br><br>For each of the following statements please indicate how much you agree/disagree that each characteristic applies to you.	1 = Disagree strongly<br>2 = Disagree a little<br>3 = Neutral; no opinion<br>4 = Agree a little<br>5 = Agree strongly<br>6 = Not applicable<br><br>Pre: M= SE= SD= Min= Max= N=<br>Post: M= SE= SD= Min= Max= N=<br>Follow: M= SE= SD= Min= Max= N=	Yes (Reverse)<br><br>Recode 6 = Missing

	I am someone who... - Avoids intellectual, philosophical discussions.		
**B5___Part_2_6**<br>**BF___Part_2_6**<br>**Q23_6_follow**	Here are a number of characteristics that may or may not apply to you. For example, do you agree that you are someone who likes to spend time with others?<br><br>For each of the following statements please indicate how much you agree/disagree that each characteristic applies to you.<br><br>I am someone who... - Is less active than other people.	1 = Disagree strongly<br>2 = Disagree a little<br>3 = Neutral; no opinion<br>4 = Agree a little<br>5 = Agree strongly<br>6 = Not applicable<br><br>Pre: M= SE= SD= Min= Max= N=<br>Post: M= SE= SD= Min= Max= N=<br>Follow: M= SE= SD= Min= Max= N=	Yes (Reverse)<br><br>Recode 6 = Missing
**B5___Part_2_7**<br>**BF___Part_2_7**<br>**Q23_7_follow**	Here are a number of characteristics that may or may not apply to you. For example, do you agree that you are someone who likes to spend time with others?<br><br>For each of the following statements please indicate how much you agree/disagree that each characteristic applies to you.<br><br>I am someone who... - Has a forgiving nature.	1 = Disagree strongly<br>2 = Disagree a little<br>3 = Neutral; no opinion<br>4 = Agree a little<br>5 = Agree strongly<br>6 = Not applicable<br><br>Pre: M= SE= SD= Min= Max= N=<br>Post: M= SE= SD= Min= Max= N=<br>Follow: M= SE= SD= Min= Max= N=	Recode 6 = Missing
**B5___Part_2_8**<br>**BF___Part_2_8**<br>**Q23_8_follow**	Here are a number of characteristics that may or may not apply to you. For example, do you agree that you are someone who likes to spend time with others?<br><br>For each of the following statements please indicate how much you agree/disagree that each characteristic applies to you.	1 = Disagree strongly<br>2 = Disagree a little<br>3 = Neutral; no opinion<br>4 = Agree a little<br>5 = Agree strongly<br>6 = Not applicable	Yes (Reverse)<br><br>Recode 6 = Missing

	I am someone who... - Can be somewhat careless.		
**B5___Part_2_9**<br>**BF___Part_2_9**<br>**Q23_9_follow**	Here are a number of characteristics that may or may not apply to you. For example, do you agree that you are someone who likes to spend time with others?<br><br>For each of the following statements please indicate how much you agree/disagree that each characteristic applies to you.<br><br>I am someone who... - Is emotionally stable, not easily upset.	1 = Disagree strongly<br>2 = Disagree a little<br>3 = Neutral; no opinion<br>4 = Agree a little<br>5 = Agree strongly<br>6 = Not applicable	Yes (Reverse)<br><br>Recode 6 = Missing
**B5___Part_2_10**<br>**BF___Part_2_10**<br>**Q23_10_follow**	Here are a number of characteristics that may or may not apply to you. For example, do you agree that you are someone who likes to spend time with others?<br><br>For each of the following statements please indicate how much you agree/disagree that each characteristic applies to you.<br><br>I am someone who... - Has little creativity.	1 = Disagree strongly<br>2 = Disagree a little<br>3 = Neutral; no opinion<br>4 = Agree a little<br>5 = Agree strongly<br>6 = Not applicable	Yes (Reverse)<br><br>Recode 6 = Missing
**B5___Part_2_11**<br>**BF___Part_2_11**<br>**Q23_11_follow**	Here are a number of characteristics that may or may not apply to you. For example, do you agree that you are someone who likes to spend time with others?<br><br>For each of the following statements please indicate how much you agree/disagree that each characteristic applies to you.<br><br>I am someone who... - Is sometimes shy, introverted.	1 = Disagree strongly<br>2 = Disagree a little<br>3 = Neutral; no opinion<br>4 = Agree a little<br>5 = Agree strongly<br>6 = Not applicable	Yes (Reverse)<br><br>Recode 6 = Missing

**B5___Part_2_12**<br>**BF___Part_2_12**<br>**Q23_12_follow**	Here are a number of characteristics that may or may not apply to you. For example, do you agree that you are someone who likes to spend time with others?<br><br>For each of the following statements please indicate how much you agree/disagree that each characteristic applies to you.<br><br>I am someone who... - Is helpful and unselfish with others.	1 = Disagree strongly<br>2 = Disagree a little<br>3 = Neutral; no opinion<br>4 = Agree a little<br>5 = Agree strongly<br>6 = Not applicable	Recode 6 = Missing
**B5___Part_2_13**<br>**BF___Part_2_13**<br>**Q23_13_follow**	Here are a number of characteristics that may or may not apply to you. For example, do you agree that you are someone who likes to spend time with others?<br><br>For each of the following statements please indicate how much you agree/disagree that each characteristic applies to you.<br><br>I am someone who... - Keeps things neat and tidy.	1 = Disagree strongly<br>2 = Disagree a little<br>3 = Neutral; no opinion<br>4 = Agree a little<br>5 = Agree strongly<br>6 = Not applicable	Recode 6 = Missing
**B5___Part_2_14**<br>**BF___Part_2_14**<br>**Q23_14_follow**	Here are a number of characteristics that may or may not apply to you. For example, do you agree that you are someone who likes to spend time with others?<br><br>For each of the following statements please indicate how much you agree/disagree that each characteristic applies to you.<br><br>I am someone who... - Worries a lot.	1 = Disagree strongly<br>2 = Disagree a little<br>3 = Neutral; no opinion<br>4 = Agree a little<br>5 = Agree strongly<br>6 = Not applicable	Recode 6 = Missing
**B5___Part_2_15**<br>**BF___Part_2_15**<br>**Q23_15_follow**	Here are a number of characteristics that may or may not apply to you.	1 = Disagree strongly<br>2 = Disagree a little	Recode 6 = Missing

	For example, do you agree that you are someone who likes to spend time with others?<br><br>For each of the following statements please indicate how much you agree/disagree that each characteristic applies to you.<br><br>I am someone who... - Values art and beauty.	3 = Neutral; no opinion<br>4 = Agree a little<br>5 = Agree strongly<br>6 = Not applicable	
**B5___Part_2_16**<br>**BF___Part_2_16**<br>**Q23_16_follow**	Here are a number of characteristics that may or may not apply to you. For example, do you agree that you are someone who likes to spend time with others?<br><br>For each of the following statements please indicate how much you agree/disagree that each characteristic applies to you.<br><br>I am someone who... - Finds it hard to influence people.	1 = Disagree strongly<br>2 = Disagree a little<br>3 = Neutral; no opinion<br>4 = Agree a little<br>5 = Agree strongly<br>6 = Not applicable	Yes (Reverse)<br><br>Recode 6 = Missing
**B5___Part_2_17**<br>**BF___Part_2_17**<br>**Q23_17_follow**	Here are a number of characteristics that may or may not apply to you. For example, do you agree that you are someone who likes to spend time with others?<br><br>For each of the following statements please indicate how much you agree/disagree that each characteristic applies to you.<br><br>I am someone who... - Is sometimes rude to others.	1 = Disagree strongly<br>2 = Disagree a little<br>3 = Neutral; no opinion<br>4 = Agree a little<br>5 = Agree strongly<br>6 = Not applicable	Yes (Reverse)<br><br>Recode 6 = Missing
**B5___Part_2_18**<br>**BF___Part_2_18**<br>**Q23_18_follow**	Here are a number of characteristics that may or may not apply to you. For example, do you agree that you are	1 = Disagree strongly<br>2 = Disagree a little<br>3 = Neutral; no opinion<br>4 = Agree a little<br>5 = Agree strongly	Recode 6 = Missing

	someone who likes to spend time with others?<br><br>For each of the following statements please indicate how much you agree/disagree that each characteristic applies to you.<br><br>I am someone who... - Is efficient, gets things done.	6 = Not applicable	
**B5___Part_2_19**<br>**BF___Part_2_19**<br>**Q23_19_follow**	Here are a number of characteristics that may or may not apply to you. For example, do you agree that you are someone who likes to spend time with others?<br><br>For each of the following statements please indicate how much you agree/disagree that each characteristic applies to you.<br><br>I am someone who... - Often feels sad.	1 = Disagree strongly<br>2 = Disagree a little<br>3 = Neutral; no opinion<br>4 = Agree a little<br>5 = Agree strongly<br>6 = Not applicable	Recode 6 = Missing
**B5___Part_2_20**<br>**BF___Part_2_20**<br>**Q23_20_follow**	Here are a number of characteristics that may or may not apply to you. For example, do you agree that you are someone who likes to spend time with others?<br><br>For each of the following statements please indicate how much you agree/disagree that each characteristic applies to you.<br><br>I am someone who... - Is complex, a deep thinker.	1 = Disagree strongly<br>2 = Disagree a little<br>3 = Neutral; no opinion<br>4 = Agree a little<br>5 = Agree strongly<br>6 = Not applicable	Recode 6 = Missing
**BF___Part_3_1**<br>**BF___Part_3_1_post**<br>**Q24_1_follow**	Here are a number of characteristics that may or may not apply to you. For example, do you agree that you are someone who likes to spend time with others?	1 = Disagree strongly<br>2 = Disagree a little<br>3 = Neutral; no opinion<br>4 = Agree a little<br>5 = Agree strongly<br>6 = Not applicable	Recode 6 = Missing

	For each of the following statements please indicate how much you agree/disagree that each characteristic applies to you.<br><br>I am someone who... - Is full of energy.		
**BF___Part_3_2<br>BF___Part_3_2_post<br>Q24_2_follow**	Here are a number of characteristics that may or may not apply to you. For example, do you agree that you are someone who likes to spend time with others?<br><br>For each of the following statements please indicate how much you agree/disagree that each characteristic applies to you.<br><br>I am someone who... - Is suspicious of others' intentions.	1 = Disagree strongly<br>2 = Disagree a little<br>3 = Neutral; no opinion<br>4 = Agree a little<br>5 = Agree strongly<br>6 = Not applicable	Yes (Reverse)<br><br>Recode 6 = Missing
**BF___Part_3_3<br>BF___Part_3_3_post<br>Q24_3_follow**	Here are a number of characteristics that may or may not apply to you. For example, do you agree that you are someone who likes to spend time with others?<br><br>For each of the following statements please indicate how much you agree/disagree that each characteristic applies to you.<br><br>I am someone who... - Is reliable, can always be counted on.	1 = Disagree strongly<br>2 = Disagree a little<br>3 = Neutral; no opinion<br>4 = Agree a little<br>5 = Agree strongly<br>6 = Not applicable	Recode 6 = Missing
**BF___Part_3_4<br>BF___Part_3_4_post<br>Q24_4_follow**	Here are a number of characteristics that may or may not apply to you. For example, do you agree that you are someone who likes to spend time with others?<br><br>For each of the following statements please indicate how much you	1 = Disagree strongly<br>2 = Disagree a little<br>3 = Neutral; no opinion<br>4 = Agree a little<br>5 = Agree strongly<br>6 = Not applicable	Yes (Reverse)<br><br>Recode 6 = Missing

	agree/disagree that each characteristic applies to you.<br><br>I am someone who... - Keeps their emotions under control.		
**BF___Part_3_5**<br>**BF___Part_3_5_post**<br>**Q24_5_follow**	Here are a number of characteristics that may or may not apply to you. For example, do you agree that you are someone who likes to spend time with others?<br><br>For each of the following statements please indicate how much you agree/disagree that each characteristic applies to you.<br><br>I am someone who... - Has difficulty imagining things.	1 = Disagree strongly<br>2 = Disagree a little<br>3 = Neutral; no opinion<br>4 = Agree a little<br>5 = Agree strongly<br>6 = Not applicable	Yes (Reverse)<br><br>Recode 6 = Missing
**BF___Part_3_6**<br>**BF___Part_3_6_post**<br>**Q24_6_follow**	Here are a number of characteristics that may or may not apply to you. For example, do you agree that you are someone who likes to spend time with others?<br><br>For each of the following statements please indicate how much you agree/disagree that each characteristic applies to you.<br><br>I am someone who... - Is talkative.	1 = Disagree strongly<br>2 = Disagree a little<br>3 = Neutral; no opinion<br>4 = Agree a little<br>5 = Agree strongly<br>6 = Not applicable	Recode 6 = Missing
**BF___Part_3_7**<br>**BF___Part_3_7_post**<br>**Q24_7_follow**	Here are a number of characteristics that may or may not apply to you. For example, do you agree that you are someone who likes to spend time with others?<br><br>For each of the following statements please indicate how much you agree/disagree that each characteristic applies to you.	1 = Disagree strongly<br>2 = Disagree a little<br>3 = Neutral; no opinion<br>4 = Agree a little<br>5 = Agree strongly<br>6 = Not applicable	Yes (Reverse)<br><br>Recode 6 = Missing

	I am someone who... - Can be cold and uncaring.		
**BF___Part_3_8**<br>**BF___Part_3_8_post**<br>**Q24_8_follow**	Here are a number of characteristics that may or may not apply to you. For example, do you agree that you are someone who likes to spend time with others?<br><br>For each of the following statements please indicate how much you agree/disagree that each characteristic applies to you.<br><br>I am someone who... - Leaves a mess, doesn't clean up.	1 = Disagree strongly<br>2 = Disagree a little<br>3 = Neutral; no opinion<br>4 = Agree a little<br>5 = Agree strongly<br>6 = Not applicable	Yes (Reverse)<br><br>Recode 6 = Missing
**BF___Part_3_9**<br>**BF___Part_3_9_post**<br>**Q24_9_follow**	Here are a number of characteristics that may or may not apply to you. For example, do you agree that you are someone who likes to spend time with others?<br><br>For each of the following statements please indicate how much you agree/disagree that each characteristic applies to you.<br><br>I am someone who... - Rarely feels anxious or afraid.	1 = Disagree strongly<br>2 = Disagree a little<br>3 = Neutral; no opinion<br>4 = Agree a little<br>5 = Agree strongly<br>6 = Not applicable	Yes (Reverse)<br><br>Recode 6 = Missing
**BF___Part_3_10**<br>**BF___Part_3_10_post**<br>**Q24_10_follow**	Here are a number of characteristics that may or may not apply to you. For example, do you agree that you are someone who likes to spend time with others?<br><br>For each of the following statements please indicate how much you agree/disagree that each characteristic applies to you.	1 = Disagree strongly<br>2 = Disagree a little<br>3 = Neutral; no opinion<br>4 = Agree a little<br>5 = Agree strongly<br>6 = Not applicable	Yes (Reverse)<br><br>Recode 6 = Missing

	I am someone who... - Thinks poetry and plays are boring.		
**BF___Part_3_11**<br>**BF___Part_3_11_post**<br>**Q24_11_follow**	Here are a number of characteristics that may or may not apply to you. For example, do you agree that you are someone who likes to spend time with others?<br><br>For each of the following statements please indicate how much you agree/disagree that each characteristic applies to you.<br><br>I am someone who... - Prefers to have others take charge.	1 = Disagree strongly<br>2 = Disagree a little<br>3 = Neutral; no opinion<br>4 = Agree a little<br>5 = Agree strongly<br>6 = Not applicable	Yes (Reverse)<br><br>Recode 6 = Missing
**BF___Part_3_12**<br>**BF___Part_3_12_post**<br>**Q24_12_follow**	Here are a number of characteristics that may or may not apply to you. For example, do you agree that you are someone who likes to spend time with others?<br><br>For each of the following statements please indicate how much you agree/disagree that each characteristic applies to you.<br><br>I am someone who... - Is polite, courteous to others.	1 = Disagree strongly<br>2 = Disagree a little<br>3 = Neutral; no opinion<br>4 = Agree a little<br>5 = Agree strongly<br>6 = Not applicable	Recode 6 = Missing
**BF___Part_3_13**<br>**BF___Part_3_13_post**<br>**Q24_13_follow**	Here are a number of characteristics that may or may not apply to you. For example, do you agree that you are someone who likes to spend time with others?<br><br>For each of the following statements please indicate how much you agree/disagree that each characteristic applies to you.	1 = Disagree strongly<br>2 = Disagree a little<br>3 = Neutral; no opinion<br>4 = Agree a little<br>5 = Agree strongly<br>6 = Not applicable	Recode 6 = Missing

	I am someone who... - Is persistent, works until the task is finished.		
**BF___Part_3_14**<br>**BF___Part_3_14_post**<br>**Q24_14_follow**	Here are a number of characteristics that may or may not apply to you. For example, do you agree that you are someone who likes to spend time with others?<br><br>For each of the following statements please indicate how much you agree/disagree that each characteristic applies to you.<br><br>I am someone who... - Tends to feel depressed, blue.	1 = Disagree strongly<br>2 = Disagree a little<br>3 = Neutral; no opinion<br>4 = Agree a little<br>5 = Agree strongly<br>6 = Not applicable	Recode 6 = Missing
**BF___Part_3_15**<br>**BF___Part_3_15_post**<br>**Q24_15_follow**	Here are a number of characteristics that may or may not apply to you. For example, do you agree that you are someone who likes to spend time with others?<br><br>For each of the following statements please indicate how much you agree/disagree that each characteristic applies to you.<br><br>I am someone who... - Has little interest in abstract ideas.	1 = Disagree strongly<br>2 = Disagree a little<br>3 = Neutral; no opinion<br>4 = Agree a little<br>5 = Agree strongly<br>6 = Not applicable	Yes (Reverse)<br><br>Recode 6 = Missing
**BF___Part_3_16**<br>**BF___Part_3_16_post**<br>**Q24_16_follow**	Here are a number of characteristics that may or may not apply to you. For example, do you agree that you are someone who likes to spend time with others?<br><br>For each of the following statements please indicate how much you agree/disagree that each characteristic applies to you.	1 = Disagree strongly<br>2 = Disagree a little<br>3 = Neutral; no opinion<br>4 = Agree a little<br>5 = Agree strongly<br>6 = Not applicable	Recode 6 = Missing

	I am someone who... - Shows a lot of enthusiasm.		
**BF___Part_3_17**<br>**BF___Part_3_17_post**<br>**Q24_17_follow**	Here are a number of characteristics that may or may not apply to you. For example, do you agree that you are someone who likes to spend time with others?<br><br>For each of the following statements please indicate how much you agree/disagree that each characteristic applies to you.<br><br>I am someone who... - Assumes the best about people.	1 = Disagree strongly<br>2 = Disagree a little<br>3 = Neutral; no opinion<br>4 = Agree a little<br>5 = Agree strongly<br>6 = Not applicable	Recode 6 = Missing
**BF___Part_3_18**<br>**BF___Part_3_18_post**<br>**Q24_18_follow**	Here are a number of characteristics that may or may not apply to you. For example, do you agree that you are someone who likes to spend time with others?<br><br>For each of the following statements please indicate how much you agree/disagree that each characteristic applies to you.<br><br>I am someone who... - Sometimes behaves irresponsibly.	1 = Disagree strongly<br>2 = Disagree a little<br>3 = Neutral; no opinion<br>4 = Agree a little<br>5 = Agree strongly<br>6 = Not applicable	Yes (Reverse)<br><br>Recode 6 = Missing
**BF___Part_3_19**<br>**BF___Part_3_19_post**<br>**Q24_19_follow**	Here are a number of characteristics that may or may not apply to you. For example, do you agree that you are someone who likes to spend time with others?<br><br>For each of the following statements please indicate how much you agree/disagree that each characteristic applies to you.	1 = Disagree strongly<br>2 = Disagree a little<br>3 = Neutral; no opinion<br>4 = Agree a little<br>5 = Agree strongly<br>6 = Not applicable	Recode 6 = Missing

	I am someone who... - Is temperamental, gets emotional easily.		
**BF___Part_3_20**<br>**BF___Part_3_20_post**<br>**Q24_20_follow**	Here are a number of characteristics that may or may not apply to you. For example, do you agree that you are someone who likes to spend time with others?<br><br>For each of the following statements please indicate how much you agree/disagree that each characteristic applies to you.<br><br>I am someone who... - Is original, comes up with new ideas.	1 = Disagree strongly<br>2 = Disagree a little<br>3 = Neutral; no opinion<br>4 = Agree a little<br>5 = Agree strongly<br>6 = Not applicable	Recode 6 = Missing

**Personality Big 5 Composite Scores**			
**Domain Scales**			
Variable Names:<br>• **Pretest**<br>• **Posttest**<br>• **Follow-up**	Variable Label	Values	Recode
**B5_Ext_pre**<br>**B5_Ext_post**<br>**B5_Ext_follow**	Composite Extraversion	B5___Part_1_1 +<br>B5___Part_1_6 +<br>B5___Part_1_11 (R) +<br>B5___Part_1_16 (R) +<br>B5___Part_2_1 +<br>B5___Part_2_6 (R) +<br>B5___Part_2_11 (R) +<br>B5___Part_2_16 (R) +<br>BF___Part_3_1 +<br>BF___Part_3_6 +<br>BF___Part_3_11 (R)<br>+ BF___Part_3_16<br><br>Pretest<br>M = 3.3774<br>Max = 5<br>Min = 1.75<br>SD = 0.78621<br>SE = 0.08790<br>N = 80<br><br>Post-test<br>M = 3.4577<br>Max = 5<br>Min = 1.50<br>SD = 0.76145	No

		SE = 0.08974<br>N = 72<br><br>Follow-up<br>M = 3.3930<br>Max = 5<br>Min = 1.58<br>SD = 0.80685<br>SE = 0.09644<br>N = 70	
**B5_Agr_pre**<br>**B5_Agr_post**<br>**B5_Agr_follow**	Composite Agreeableness	B5___Part_1_2 +<br>B5___Part_1_7 +<br>B5___Part_1_12 (R) +<br>B5___Part_1_17 (R) +<br>B5___Part_2_2 (R) +<br>B5___Part_2_7 +<br>B5___Part_2_12 +<br>B5___Part_2_17 (R) +<br>BF___Part_3_2 (R) +<br>BF___Part_3_7 (R) +<br>BF___Part_3_12 +<br>BF___Part_3_17<br><br>Pretest<br>M = 3.9181<br>Max = 4.92<br>Min = 2.17<br>SD = 0.63682<br>SE = 0.07120<br>N = 80<br><br>Post-test<br>M = 3.8750<br>Max = 5<br>Min = 2.42<br>SD = 0.60330<br>SE = 0.07110<br>N = 72<br><br>Follow-up<br>M = 3.7415<br>Max = 5<br>Min = 2.25<br>SD = 0.62915<br>SE = 0.07314<br>N =74	No
**B5_Con_pre**<br>**B5_Con_post**<br>**B5_Con_follow**	Composite Conscientiousness	B5___Part_1_3 (R) +<br>B5___Part_1_8 (R) +<br>B5___Part_1_13 +<br>B5___Part_1_18 +<br>B5___Part_2_3 (R) +<br>B5___Part_2_8 (R) +<br>B5___Part_2_13 +<br>B5___Part_2_18 +	No

		BF___Part_3_3 +<br>BF___Part_3_8 (R) +<br>BF___Part_3_13 +<br>BF___Part_3_18 (R)<br><br>Pretest<br>M = 3.8872<br>Max = 5<br>Min = 1.83<br>SD = 0.75468<br>SE = 0.08438<br>N = 80<br><br>Post-test<br>M = 3.9063<br>Max = 5<br>Min = 2.33<br>SD = 0.72836<br>SE = 0.08584<br>N = 72<br><br>Follow-up<br>M = 3.8314<br>Max = 5<br>Min = 2<br>SD = 0.68999<br>SE = 0.08021<br>N =74	
**B5_Neuro_pre**<br>**B5_Neuro_post**<br>**B5_Neuro_follow**	Composite Negative Emotionality	B5___Part_1_4 (R) +<br>B5___Part_1_9 (R) +<br>B5___Part_1_14 +<br>B5___Part_1_19 +<br>B5___Part_2_4 (R) +<br>B5___Part_2_9 (R) +<br>B5___Part_2_14 +<br>B5___Part_2_19 +<br>BF___Part_3_4 (R) +<br>BF___Part_3_9 (R) +<br>BF___Part_3_14 +<br>BF___Part_3_19<br><br>Pretest<br>M = 2.8615<br>Max = 5<br>Min = 1<br>SD = 0.94982<br>SE = 0.10619<br>N = 80<br><br>Post-test<br>M = 2.8056<br>Max = 4.50<br>Min = 1.10<br>SD = 0.87502<br>SE = 0.10312	No

		N = 72<br><br>Follow-up<br>M = 2.7952<br>Max = 4.75<br>Min = 1.08<br>SD = 0.90597<br>SE = 0.10828<br>N = 70	
**B5_Open_pre**<br>**B5_Open_post**<br>**B5_Open_follow**	Composite Open-Mindedness	B5___Part_1_5 (R) + B5___Part_1_10 + B5___Part_1_15 + B5___Part_1_20 + B5___Part_2_5 (R) + B5___Part_2_10 (R) + B5___Part_2_15 + B5___Part_2_20 + BF___Part_3_5 (R) + BF___Part_3_10 (R) + BF___Part_3_15 (R) + BF___Part_3_20<br><br>Pretest<br>M = 4.0559<br>Max = 5<br>Min = 2.25<br>SD = 0.68609<br>SE = 0.07671<br>N = 80<br><br>Post-test<br>M = 3.9898<br>Max = 5<br>Min = 2.17<br>SD = 0.72650<br>SE = 0.08562<br>N = 72<br><br>Follow-up<br>M = 3.9513<br>Max = 5<br>Min = 2.08<br>SD = 0.72441<br>SE = 0.08658<br>N = 70	No

**Big Five Facet Scales**			
**Variable Names:**<br>• **Pretest**<br>• **Posttest**<br>• **Follow-up**	Variable Label	Values	Recode?

**B5_Soc_pre**<br>**B5_Soc_post**<br>**B5_Soc_follow**	Composite Sociability	B5___Part_1_1 +<br>B5___Part_1_16 (R) +<br>B5___Part_2_11 (R) +<br>BF___Part_3_6<br><br>Pretest<br>M = 3.2500<br>Max = 5<br>Min = 1<br>SD = 1.07105<br>SE = 0.11975<br>N = 80<br><br>Post-test<br>M = 3.2269<br>Max = 5<br>Min = 1<br>SD = 0.98891<br>SE = 0.11654<br>N = 72<br><br>Follow-up<br>M = 3.2821<br>Max = 5<br>Min = 1.25<br>SD = 1.01343<br>SE = 0.12113<br>N = 70	No
**B5_Assert_pre**<br>**B5_Assert_post**<br>**B5_Assert_follow**	Composite Assertiveness	B5___Part_1_6 +<br>B5___Part_2_1 +<br>B5___Part_2_16 (R) +<br>BF___Part_3_11 (R)<br><br>Pretest<br>M = 3.4302<br>Max = 5<br>Min = 1.25<br>SD = 0.95351<br>SE = 0.10661<br>N = 80<br><br>Post-test<br>M = 3.4583<br>Max = 5<br>Min = 1<br>SD = 1.01697<br>SE = 0.11985<br>N = 72<br><br>Follow-up<br>M = 3.3167<br>Max = 5<br>Min = 1.25<br>SD = 1.03274<br>SE = 0.12344	No

		N = 70	
**B5_Ene_pre**<br>**B5_Ene_post**<br>**B5_Ene_follow**	Composite Energy Level	B5___Part_1_11 (R) +<br>B5___Part_2_6 (R) +<br>BF___Part_3_1 +<br>BF___Part_3_16<br><br>Pretest<br>M = 3.4500<br>Max = 5<br>Min = 1.75<br>SD = 0.83666<br>SE = 0.09354<br>N = 80<br><br>Post-test<br>M = 3.6829<br>Max = 5<br>Min = 1.25<br>SD = 0.84332<br>SE = 0.09939<br>N = 72<br><br>Follow-up<br>M = 3.5798<br>Max = 5<br>Min = 1.75<br>SD = 0.85884<br>SE = 0.10265<br>N = 70	No
**B5_Com_pre**<br>**B5_Com_post**<br>**B5_Com_follow**	Composite Compassion	B5___Part_1_2 +<br>B5___Part_1_17 (R) +<br>B5___Part_2_12 +<br>BF___Part_3_7 (R)<br><br>Pretest<br>M = 4.2271<br>Max = 5<br>Min = 2.25<br>SD = 0.70436<br>SE = 0.07875<br>N = 80<br><br>Post-test<br>M = 4.1065<br>Max = 5<br>Min = 2.50<br>SD = 0.78314<br>SE = 0.09229<br>N = 72<br><br>Follow-up<br>M = 4.0679<br>Max = 5<br>Min = 2.50	No

		SD = 0.74079<br>SE = 0.08854<br>N = 70	
**B5_Res_pre**<br>**B5_Res_post**<br>**B5_Res_follow**	Composite Respectfulness	B5___Part_1_7 +<br>B5___Part_2_2 (R) +<br>B5___Part_2_17 (R) +<br>BF___Part_3_12<br><br>Pretest<br>M = 4.2469<br>Max = 5<br>Min = 2.50<br>SD = 0.73508<br>SE = 0.08218<br>N = 80<br><br>Post-test<br>M = 4.2095<br>Max = 5<br>Min = 2.50<br>SD = 0.66092<br>SE = 0.07789<br>N = 72<br><br>Follow-up<br>M = 4.2179<br>Max = 5<br>Min = 3<br>SD = 0.68090<br>SE = 0.08138<br>N = 70	No
**B5_Tru_pre**<br>**B5_Tru_post**<br>**B5_Tru_follow**	Composite Trust	B5___Part_1_12 (R) +<br>B5___Part_2_7 +<br>BF___Part_3_2 (R) +<br>BF___Part_3_17<br><br>Pretest<br>M = 3.2823<br>Max = 5<br>Min = 1.25<br>SD = 0.89247<br>SE = 0.09978<br>N = 80<br><br>Post-test<br>M = 3.3079<br>Max = 5<br>Min = 1.25<br>SD = 0.89792<br>SE = 0.10582<br>N = 72<br><br>Follow-up<br>M = 3.2310	No

		Max = 5<br>Min = 1<br>SD = 0.94379<br>SE = 0.11280<br>N = 70	
**B5_Org_pre**<br>**B5_Org_post**<br>**B5_Org_follow**	Composite Organization	B5___Part_1_3 (R) +<br>B5___Part_1_18 +<br>B5___Part_2_13 +<br>BF___Part_3_8 (R)<br><br>Pretest<br>M = 3.8708<br>Max = 5<br>Min = 1<br>SD = 0.93154<br>SE = 0.10415<br>N = 80<br><br>Post-test<br>M = 3.7674<br>Max = 5<br>Min = 1.75<br>SD = 0.96353<br>SE = 0.11355<br>N = 72<br><br>Follow-up<br>M = 3.7524<br>Max = 5<br>Min = 1<br>SD = 0.99384<br>SE = 0.11879<br>N = 70	No
**B5_Prod_pre**<br>**B5_Prod_post**<br>**B5_Prod_follow**	Compositive Productiveness	B5___Part_1_8 (R) +<br>B5___Part_2_3 (R) +<br>B5___Part_2_18 +<br>BF___Part_3_13<br><br>Pretest<br>M = 3.7813<br>Max = 5<br>Min = 1.50<br>SD = 0.85070<br>SE = 0.09511<br>N = 80<br><br>Post-test<br>M = 3.8819<br>Max = 5<br>Min = 2.25<br>SD = 0.80160<br>SE = 0.09447<br>N = 72	No

		Follow-up<br>M = 3.9274<br>Max = 5<br>Min = 2.25<br>SD = 0.87584<br>SE = 0.10468<br>N = 70	
**B5_Resp_pre**<br>**B5_Resp_post**<br>**B5_Resp_follow**	Composite Responsibility	B5___Part_1_13 +<br>B5___Part_2_8 (R) +<br>BF___Part_3_3 +<br>BF___Part_3_18 (R)<br><br>Pretest<br>M = 4.0125<br>Max = 5<br>Min = 1.50<br>SD = 0.91480<br>SE = 0.10228<br>N = 80<br><br>Post-test<br>M = 4.0694<br>Max = 5<br>Min = 1.75<br>SD = 0.81169<br>SE = 0.09566<br>N = 72<br><br>Follow-up<br>M = 4.0357<br>Max = 5<br>Min = 2.25<br>SD = 0.77235<br>SE = 0.09231<br>N = 70	No
**B5_Anx_pre**<br>**B5_Anx_post**<br>**B5_Anx**	Composite Anxiety	B5___Part_1_4 (R) +<br>B5___Part_1_19 +<br>B5___Part_2_14 +<br>BF___Part_3_9 (R)<br><br>Pretest<br>M = 3.3813<br>Max = 5<br>Min = 1<br>SD = 0.99124<br>SE = 0.11082<br>N = 80<br><br>Post-test<br>M = 3.2500<br>Max = 5<br>Min = 1<br>SD = 0.97323	No

		SE = 0.11470<br>N = 72<br><br>Follow-up<br>M = 3.2250<br>Max = 5<br>Min = 1<br>SD = 1.02959<br>SE = 0.12307<br>N = 70	
**B5_Depr_pre**<br>**B5_Depr_post**<br>**B5_Depr_follow**	Composite Depression	B5___Part_1_9 (R) +<br>B5___Part_2_4 (R) +<br>B5___Part_2_19 +<br>BF___Part_3_14<br><br>Pretest<br>M = 2.5354<br>Max = 5<br>Min = 1<br>SD = 1.10314<br>SE = 0.12333<br>N = 80<br><br>Post-test<br>M = 2.4317<br>Max = 4.75<br>Min = 1<br>SD = 0.96052<br>SE = 0.11320<br>N = 72<br><br>Follow-up<br>M = 2.5429<br>Max = 4.25<br>Min = 1<br>SD = 0.97149<br>SE = 0.11612<br>N = 70	No
**B5_Emot_pre**<br>**B5_Emot_post**<br>**B5_Emot_follow**	Composite Emotional Volatility	B5___Part_1_14 +<br>B5___Part_2_9 (R) +<br>BF___Part_3_4 (R) +<br>BF___Part_3_19<br><br>Pretest<br>M = 2.6625<br>Max = 5<br>Min = 1<br>SD = 1.08943<br>SE = 0.12180<br>N = 80<br><br>Post-test<br>M = 2.7269<br>Max = 5	No

		Min = 1<br>SD = 1.09275<br>SE = 0.12878<br>N = 72<br><br>Follow-up<br>M = 2.6179<br>Max = 5<br>Min = 1<br>SD = 1.02657<br>SE = 0.12270<br>N = 70	
**B5_Intel_pre<br>B5_Intel_post<br>B5_Intel_follow**	Composite Intellectual Curiosity	B5___Part_1_10 +<br>B5___Part_2_5 (R) +<br>B5___Part_2_20 +<br>BF___Part_3_15 (R)<br><br>Pretest<br>M = 4.1156<br>Max = 5<br>Min = 1.75<br>SD = 0.76175<br>SE = 0.08517<br>N = 80<br><br>Post-test<br>M = 3.9780<br>Max = 5<br>Min = 1.25<br>SD = 0.90166<br>SE = 0.10626<br>N = 72<br><br>Follow-up<br>M = 4.0012<br>Max = 5<br>Min = 1.75<br>SD = 0.82049<br>SE = 0.09807<br>N = 70	No
**B5_Aesth_pre<br>B5_Aesth_post<br>B5_Aesth_follow**	Composite Aesthetic Sensitivity	B5___Part_1_5 (R) +<br>B5___Part_1_20 +<br>B5___Part_2_15 +<br>BF___Part_3_10 (R)<br><br>Pretest<br>M = 3.8927<br>Max = 5<br>Min = 1.50<br>SD = 0.84944<br>SE = 0.09497<br>N = 80<br><br>Post-test	No

		M = 3.8785<br>Max = 5<br>Min = 1.50<br>SD = 0.85167<br>SE = 0.10037<br>N = 72<br><br>Follow-up<br>M = 3.7619<br>Max = 5<br>Min = 1.25<br>SD = 0.91433<br>SE = 0.10928<br>N = 70	
**B5_Cre_pre**<br>**B5_Cre_post**<br>**B5_Cre_follow**	Composite Creative Imagination	B5___Part_1_15 +<br>B5___Part_2_10 (R) +<br>BF___Part_3_5 (R) +<br>BF___Part_3_20<br><br>Pretest<br>M = 4.1583<br>Max = 5<br>Min = 2.25<br>SD = 0.76670<br>SE = 0.08572<br>N = 80<br><br>Post-test<br>M = 4.1146<br>Max = 5<br>Min = 1.75<br>SD = 0.77589<br>SE = 0.09144<br>N = 72<br><br>Follow-up<br>M = 4.0857<br>Max = 5<br>Min = 2.25<br>SD = 0.78827<br>SE = 0.09422<br>N = 70	No

**Exercise Self-efficacy**			
Variable Names:<br>• **Pretest**<br>• **Posttest**<br>• **Follow-up**	Variable Label	Values	Recoded such that higher scores = higher self-efficacy
**Exercise_SE_1**<br>**Q27_1**<br>**Q26_1**	How sure are you that you will do each of the following: - Exercise	1 = Very sure<br>2 = Pretty sure<br>3 = A little sure	1=4<br>2=3<br>3=2

	regularly (e.g., 3 times a week for 20 minutes)	4 = Not at all sure<br>5 = Not applicable	4=1<br>5 = Missing<br><br>Pre: M=2.8974<br>SE=0.11626<br>SD=1.02678 Min=1<br>Max=4 N=78<br>Post: M=2.6528<br>SE=0.12535<br>SD=1.06361 Min=1<br>Max=4 N=72<br>Follow: M=2.6667<br>SE=0.12838<br>SD=1.06642 Min=1<br>Max=4 N=69
**Exercise_SE_2<br>Q27_2<br>Q26_2**	How sure are you that you will do each of the following: - Exercise when you are feeling tired	1 = Very sure<br>2 = Pretty sure<br>3 = A little sure<br>4 = Not at all sure<br>5 = Not applicable	1=4<br>2=3<br>3=2<br>4=1<br>5 = Missing<br><br>Pre: M=2.3816<br>SE=0.12561<br>SD=1.09504 Min=1<br>Max=4 N=76<br>Post: M=2.1389<br>SE=0.10889<br>SD=0.92395 Min=1<br>Max=4 N=72<br>Follow: M=2.1429<br>SE=0.11382<br>SD=0.95228 Min=1<br>Max=4 N=70
**Exercise_SE_3<br>Q27_3<br>Q26_3**	How sure are you that you will do each of the following: - Exercise when you are feeling under pressure to get things done	1 = Very sure<br>2 = Pretty sure<br>3 = A little sure<br>4 = Not at all sure<br>5 = Not applicable	1=4<br>2=3<br>3=2<br>4=1<br>5 = Missing<br><br>Pre: M=2.3896<br>SE=0.12560<br>SD=1.10210 Min=1<br>Max=4 N=77<br>Post: M=2.1268<br>SE=0.11335<br>SD=0.95513 Min=1<br>Max=4 N=71<br>Follow: M=2.0145<br>SE=0.11021<br>SD=0.91544 Min=1<br>Max=4 N=69
**Exercise_SE_4<br>Q27_4**	How sure are you that you will do each of the	1 = Very sure<br>2 = Pretty sure	1=4<br>2=3

**Q26_4**	following: - Exercise when you are feeling down or depressed	3 = A little sure<br>4 = Not at all sure<br>5 = Not applicable	3=2<br>4=1<br>5 = Missing<br><br>Pre: M=2.5132 SE=0.13178 SD=1.14884 Min=1 Max=4 N=76<br>Post: M=2.0833 SE=0.11491 SD=0.97504 Min=1 Max=4 N=72<br>Follow: M=2.0290 SE=0.10919 SD=0.90702 Min=1 Max=4 N=69
**Exercise_SE_5**<br>**Q27_5**<br>**Q26_5**	How sure are you that you will do each of the following: - Exercise when you have too much work to do at home	1 = Very sure<br>2 = Pretty sure<br>3 = A little sure<br>4 = Not at all sure<br>5 = Not applicable	1=4<br>2=3<br>3=2<br>4=1<br>5 = Missing<br><br>Pre: M=2.2179 SE=0.11917 SD=1.05249 Min=1 Max=4 N=78<br>Post: M=1.9565 SE=0.10814 SD=0.89828 Min=1 Max=4 N=69<br>Follow: M=1.8986 SE=0.10557 SD=0.87691 Min=1 Max=4 N=69
**Exercise_SE_6**<br>**Q27_6**<br>**Q26_6**	How sure are you that you will do each of the following: - Exercise when there are other more interesting things to do	1 = Very sure<br>2 = Pretty sure<br>3 = A little sure<br>4 = Not at all sure<br>5 = Not applicable	1=4<br>2=3<br>3=2<br>4=1<br>5 = Missing<br><br>Pre: M=2.4359 SE=0.12979 SD=1.14631 Min=1 Max=4 N=78<br>Post: M=2.2676 SE=0.11690 SD=0.98500 Min=1 Max=4 N=71<br>Follow: M=2.1176 SE=0.12132 SD=1.00044 Min=1 Max=4 N=68

**Exercise_SE_7**<br>**Q27_7**<br>**Q26_7**	How sure are you that you will do each of the following: - Exercise when your family or friends do not provide any kind of support	1 = Very sure<br>2 = Pretty sure<br>3 = A little sure<br>4 = Not at all sure<br>5 = Not applicable	1=4<br>2=3<br>3=2<br>4=1<br>5 = Missing<br><br>Pre: M=2.6351<br>SE=0.14344<br>SD=1.23393 Min=1<br>Max=4 N=74<br>Post: M=2.5571<br>SE=0.13595<br>SD=1.13745 Min=1<br>Max=4 N=70<br>Follow: M=2.4412<br>SE=0.13148<br>SD=1.08425 Min=1<br>Max=4 N=68
**Exercise_SE_8**<br>**Q27_8**<br>**Q26_8**	How sure are you that you will do each of the following: - Exercise when you don't really feel like it	1 = Very sure<br>2 = Pretty sure<br>3 = A little sure<br>4 = Not at all sure<br>5 = Not applicable	1=4<br>2=3<br>3=2<br>4=1<br>5 = Missing<br><br>Pre: M=2.2692<br>SE=0.11494<br>SD=1.01512 Min=1<br>Max=4 N=78<br>Post: M=2.1831<br>SE=0.12253<br>SD=1.03247 Min=1<br>Max=4 N=71<br>Follow: M=2.0725<br>SE=0.10389<br>SD=0.86294 Min=1<br>Max=4 N=69
**Exercise_SE_9**<br>**Q27_9**<br>**Q26_9**	How sure are you that you will do each of the following: - Exercise when you are away from home (e.g., traveling, visiting, on vacation)	1 = Very sure<br>2 = Pretty sure<br>3 = A little sure<br>4 = Not at all sure<br>5 = Not applicable	1=4<br>2=3<br>3=2<br>4=1<br>5 = Missing<br><br>Pre: M=2.4533<br>SE=0.11747<br>SD=1.01733 Min=1<br>Max=4 N=75<br>Post: M=2.2143<br>SE=0.12690<br>SD=1.06176 Min=1<br>Max=4 N=70<br>Follow: M=2.2899<br>SE=0.12405<br>SD=1.03044 Min=1<br>Max=4 N=69

Composite Exercise Self Efficacy			
Variable Names:<br>• **Pretest**<br>• **Posttest**<br>• **Follow-up**	Variable Label	Values	Recode?
**ExerSelfEff_pre**<br>**ExerSelfEff_post**<br>**ExerSelfEff_follow**	Composite Exercise Self Efficacy	Exercise_SE_1 +<br>Exercise_SE_2 +<br>Exercise_SE_3 +<br>Exercise_SE_4 +<br>Exercise_SE_5 +<br>Exercise_SE_6 +<br>Exercise_SE_7 +<br>Exercise_SE_8 +<br>Exercise_SE_9<br><br>Pretest<br>M = 2.4489<br>Max = 4<br>Min = 1<br>SD = 0.90922<br>SE = 0.10230<br>N = 79<br><br>Post-test<br>M = 2.2445<br>Max = 3.89<br>Min = 1<br>SD = 0.78705<br>SE = 0.09276<br>N = 72<br><br>Follow-up<br>M = 2.1992<br>Max = 3.89<br>Min = 1<br>SD = 0.77390<br>SE = 0.09250<br>N = 70	No

Satisfaction with Life			
Variable Names:<br>• **Pretest**<br>• **Posttest**<br>• **Follow-up**	Variable Label	Values	Recode?
**Life_sat_1**<br>**Q75_1**<br>**Q74_1**	Below are five statements that you may agree or disagree with. Using the scale below, indicate your agreement with each item. - In most	9 = Strongly disagree<br>10 = Disagree<br>11 = Slightly disagree<br>12 = Neither agree nor disagree<br>13 = Slightly agree	Recode<br>9 = 1<br>10 = 2<br>11 = 3<br>12 = 4<br>13 = 5

	ways my life is close to my ideal	14 = Agree<br>15 = Strongly agree<br>16 = Not applicable	14 = 6<br>15 = 7<br>16 = Missing<br><br>Pre: M=3.9620<br>SE=0.21350<br>SD=1.89766 Min=1<br>Max=7 N=79<br>Post: M=4.2535<br>SE=0.21108<br>SD=1.77859 Min=1<br>Max=7 N=71<br>Follow: M=4.3143<br>SE=0.21004<br>SD=1.75733 Min=1<br>Max=7 N=70
**Life_sat_2**<br>**Q75_2**<br>**Q74_2**	Below are five statements that you may agree or disagree with. Using the scale below, indicate your agreement with each item. - The conditions of my life are excellent	9 = Strongly disagree<br>10 = Disagree<br>11 = Slightly disagree<br>12 = Neither agree nor disagree<br>13 = Slightly agree<br>14 = Agree<br>15 = Strongly agree<br>16 = Not applicable	Recode<br>9 = 1<br>10 = 2<br>11 = 3<br>12 = 4<br>13 = 5<br>14 = 6<br>15 = 7<br>16 = Missing<br><br>Pre: M=4.4875<br>SE=0.19648<br>SD=1.75740 Min=1<br>Max=7 N=80<br>Post: M=4.6620<br>SE=0.21034<br>SD=1.77236 Min=1<br>Max=7 N=71<br>Follow: M=4.6232<br>SE=0.20134<br>SD=1.67245 Min=1<br>Max=7 N=69
**Life_sat_3**<br>**Q75_3**<br>**Q74_3**	Below are five statements that you may agree or disagree with. Using the scale below, indicate your agreement with each item. - I am satisfied with my life	9 = Strongly disagree<br>10 = Disagree<br>11 = Slightly disagree<br>12 = Neither agree nor disagree<br>13 = Slightly agree<br>14 = Agree<br>15 = Strongly agree<br>16 = Not applicable	Recode<br>9 = 1<br>10 = 2<br>11 = 3<br>12 = 4<br>13 = 5<br>14 = 6<br>15 = 7<br>16 = Missing<br><br>Pre: M=4.7250<br>SE=0.18740<br>SD=1.67615 Min=1<br>Max=7 N=80<br>Post: M=4.6761<br>SE=0.20237

			SD=1.70524 Min=1<br>Max=7 N=71<br>Follow: M=4.7571<br>SE=0.18919<br>SD=1.58284 Min=1<br>Max=7 N=70
**Life_sat_4**<br>**Q75_4**<br>**Q74_4**	Below are five statements that you may agree or disagree with. Using the scale below, indicate your agreement with each item. - So far I have gotten the important things I want in life	9 = Strongly disagree<br>10 = Disagree<br>11 = Slightly disagree<br>12 = Neither agree nor disagree<br>13 = Slightly agree<br>14 = Agree<br>15 = Strongly agree<br>16 = Not applicable	Recode<br>9 = 1<br>10 = 2<br>11 = 3<br>12 = 4<br>13 = 5<br>14 = 6<br>15 = 7<br>16 = Missing<br><br>Pre: M=4.9000<br>SE=0.19044<br>SD=1.70331 Min=1<br>Max=7 N=80<br>Post: M=5.0282<br>SE=0.20057<br>SD=1.69007 Min=1<br>Max=7 N=71<br>Follow: M=4.8824<br>SE=0.19825<br>SD=1.63478 Min=1<br>Max=7 N=68
**Life_sat_5**<br>**Q75_5**<br>**Q74_5**	Below are five statements that you may agree or disagree with. Using the scale below, indicate your agreement with each item. - If I could live my life over, I would change almost nothing	9 = Strongly disagree<br>10 = Disagree<br>11 = Slightly disagree<br>12 = Neither agree nor disagree<br>13 = Slightly agree<br>14 = Agree<br>15 = Strongly agree<br>16 = Not applicable	Recode<br>9 = 1<br>10 = 2<br>11 = 3<br>12 = 4<br>13 = 5<br>14 = 6<br>15 = 7<br>16 = Missing<br><br>Pre: M=3.3750<br>SE=0.21887<br>SD=1.95762 Min=1<br>Max=7 N=80<br>Post: M=3.6429<br>SE=0.25419<br>SD=2.12668 Min=1<br>Max=7 N=70<br>Follow: M=3.5714<br>SE=0.25462<br>SD=2.13033 Min=1<br>Max=7 N=70

**Satisfaction with Life Composite Variable**			
Variable Names:	Variable Label	Values	Recode?

• **Pretest**<br>• **Posttest**<br>• **Follow-up**			
**Life_Sat_pre**<br>**Life_Sat_post**<br>**Life_Sat_follow**	Composite Life Satisfaction	Life_sat_1 +<br>Life_sat_2 +<br>Life_sat_3 +<br>Life_sat_4 +<br>Life_sat_5<br><br>Pretest<br>M = 4.2888<br>Max = 7<br>Min = 1<br>SD = 1.46097<br>SE = 0.16334<br>N = 80<br><br>Post-test<br>M = 4.4472<br>Max = 7<br>Min = 1<br>SD = 1.58990<br>SE = 0.18869<br>N = 71<br><br>Follow-up<br>M = 4.4274<br>Max = 7<br>Min = 1<br>SD = 1.52354<br>SE = 0.18210<br>N = 70	No

**Pretest- Additional Health Rating**			
Variable Name	Variable Label	Values	Recode?
**AHR**	In general, compared to most men/women your age, would you say your health is...	1 = Much better<br>2 = Somewhat better<br>3 = About the same<br>4 = Somewhat worse<br>5 = Much worse<br>6 = Don't know<br>7 = Not applicable	Recode 7 = Missing<br><br>M = 2.7500<br>Max = 5<br>Min = 1<br>SD = 1.01258<br>SE = 0.11321<br>N = 80

**MIDUS Sense of Control**			
Variable Name:<br>• **Pretest**<br>• **Posttest**<br>• **Follow-up**	Variable Label	Values	Recode?

**Sense_of_Control_1**<br>**Q34_1**<br>**Q33_1**	For each statement below please click the answer that best represents your level of agreement. - There is little I can do to change many of the important things in my life.	1 = Strongly disagree<br>2 = Somewhat disagree<br>3 = Disagree a little<br>4 = Don't know<br>5 = Agree a little<br>6 = Somewhat agree<br>7 = Strongly agree<br>8 = Not applicable	Recode 8 = Missing<br><br>Pre: M=2.8250 SE=0.20108 SD=1.79856 Min=1 Max=7 N=80<br>Post: M=3.0556 SE=0.22281 SD=1.89059 Min=1 Max=7 N=72<br>Follow: M=3.1714 SE=0.23020 SD=1.92596 Min=1 Max=7 N=70
**Sense_of_Control_2**<br>**Q34_2**<br>**Q33_2**	For each statement below please click the answer that best represents your level of agreement. - I often feel helpless in dealing with the problems of life.	1 = Strongly disagree<br>2 = Somewhat disagree<br>3 = Disagree a little<br>4 = Don't know<br>5 = Agree a little<br>6 = Somewhat agree<br>7 = Strongly agree<br>8 = Not applicable	Recode 8 = Missing<br><br>Pre: M=3.3750 SE=0.23220 SD=2.07685 Min=1 Max=7 N=80<br>Post: M=3.5000 SE=0.22982 SD=1.95008 Min=1 Max=7 N=72<br>Follow: M=3.4857 SE=0.23795 SD=1.99087 Min=1 Max=7 N=70
**Sense_of_Control_3**<br>**Q34_3**<br>**Q33_3**	For each statement below please click the answer that best represents your level of agreement. - I can do just about anything I really set my mind to do.	1 = Strongly disagree<br>2 = Somewhat disagree<br>3 = Disagree a little<br>4 = Don't know<br>5 = Agree a little<br>6 = Somewhat agree<br>7 = Strongly agree<br>8 = Not applicable	Recode 8 = Missing<br><br>Pre: M=5.6875 SE=0.17393 SD=1.55567 Min=1 Max=7 N=80<br>Post: M=5.5000 SE=0.20171 SD=1.71160 Min=1 Max=7 N=72<br>Follow: M=5.4857 SE=0.20626 SD=1.72570 Min=1 Max=7 N=70
**Sense_of_Control_4**<br>**Q34_4**<br>**Q33_4**	For each statement below please click the answer that best represents your level of agreement. - Other people determine most what I can and cannot do.	1 = Strongly disagree<br>2 = Somewhat disagree<br>3 = Disagree a little<br>4 = Don't know<br>5 = Agree a little<br>6 = Somewhat agree<br>7 = Strongly agree<br>8 = Not applicable	Recode 8 = Missing<br><br>Pre: M=2.3671 SE=0.18651 SD=1.65775 Min=1 Max=7 N=79<br>Post: M=2.7500 SE=0.21914 SD=1.85950 Min=1 Max=7 N=72

			Follow: M=2.7571<br>SE=0.21092<br>SD=1.76468 Min=1<br>Max=7 N=70
**Sense_of_Control_5<br>Q34_5<br>Q33_5**	For each statement below please click the answer that best represents your level of agreement. - What happens in my life is often beyond my control.	1 = Strongly disagree<br>2 = Somewhat disagree<br>3 = Disagree a little<br>4 = Don't know<br>5 = Agree a little<br>6 = Somewhat agree<br>7 = Strongly agree<br>8 = Not applicable	Recode 8 = Missing<br><br>Pre: M=3.4000<br>SE=0.22474<br>SD=2.01010 Min=1<br>Max=7 N=80<br>Post: M=3.0556<br>SE=0.20640<br>SD=1.75137 Min=1<br>Max=7 N=72<br>Follow: M=3.2143<br>SE=0.22466<br>SD=1.87966 Min=1<br>Max=7 N=70
**Sense_of_Control_6<br>Q34_6<br>Q33_6**	For each statement below please click the answer that best represents your level of agreement. - When I really want to do something, I usually find a way to succeed at it.	1 = Strongly disagree<br>2 = Somewhat disagree<br>3 = Disagree a little<br>4 = Don't know<br>5 = Agree a little<br>6 = Somewhat agree<br>7 = Strongly agree<br>8 = Not applicable	Recode 8 = Missing<br><br>Pre: M=6.0506<br>SE=0.12853<br>SD=1.14241 Min=2<br>Max=7 N=79<br>Post: M=6.0000<br>SE=0.13704<br>SD=1.16280 Min=1<br>Max=7 N=72<br>Follow: M=5.9275<br>SE=0.14645<br>SD=1.21654 Min=2<br>Max=7 N=69
**Sense_of_Control_7<br>Q34_7<br>Q33_7**	For each statement below please click the answer that best represents your level of agreement. - There are many things that interfere with what I want to do.	1 = Strongly disagree<br>2 = Somewhat disagree<br>3 = Disagree a little<br>4 = Don't know<br>5 = Agree a little<br>6 = Somewhat agree<br>7 = Strongly agree<br>8 = Not applicable	Recode 8 = Missing<br><br>Pre: M=4.2750<br>SE=0.21713<br>SD=1.94204 Min=1<br>Max=7 N=80<br>Post: M=4.5139<br>SE=0.22202<br>SD=1.88391 Min=1<br>Max=7 N=72<br>Follow: M=4.2206<br>SE=0.23504<br>SD=1.93819 Min=1<br>Max=7 N=68
**Sense_of_Control_8<br>Q34_8<br>Q33_8**	For each statement below please click the answer that best represents your level of agreement. - Whether or not I am able to get	1 = Strongly disagree<br>2 = Somewhat disagree<br>3 = Disagree a little<br>4 = Don't know<br>5 = Agree a little<br>6 = Somewhat agree	Recode 8 = Missing<br><br>Pre: M=5.2750<br>SE=0.18740<br>SD=1.67615 Min=1<br>Max=7 N=80

	what I want is in my own hands.	7 = Strongly agree<br>8 = Not applicable	Post: M=5.2917 SE=0.18176 SD=1.54225 Min=1 Max=7 N=72<br>Follow: M=5.3429 SE=0.18748 SD=1.56858 Min=1 Max=7 N=70
**Sense_of_Control_9**<br>**Q34_9**<br>**Q33_9**	For each statement below please click the answer that best represents your level of agreement. - I have little control over the things that happen to me.	1 = Strongly disagree<br>2 = Somewhat disagree<br>3 = Disagree a little<br>4 = Don't know<br>5 = Agree a little<br>6 = Somewhat agree<br>7 = Strongly agree<br>8 = Not applicable	Recode 8 = Missing<br><br>Pre: M=3.3000 SE=0.21153 SD=1.89202 Min=1 Max=7 N=80<br>Post: M=3.0278 SE=0.21024 SD=1.78390 Min=1 Max=7 N=72<br>Follow: M=2.9571 SE=0.21092 SD=1.76468 Min=1 Max=7 N=70
**Sense_of_Control_10**<br>**Q34_10**<br>**Q33_10**	For each statement below please click the answer that best represents your level of agreement. - There is really no way I can solve some of the problems I have.	1 = Strongly disagree<br>2 = Somewhat disagree<br>3 = Disagree a little<br>4 = Don't know<br>5 = Agree a little<br>6 = Somewhat agree<br>7 = Strongly agree<br>8 = Not applicable	Recode 8 = Missing<br><br>Pre: M=3.4375 SE=0.23320 SD=2.08578 Min=1 Max=7 N=80<br>Post: M=3.3611 SE=0.23843 SD=2.02314 Min=1 Max=7 N=72<br>Follow: M=3.2429 SE=0.25613 SD=2.14297 Min=1 Max=7 N=70
**Sense_of_Control_11**<br>**Q34_11**<br>**Q33_11**	For each statement below please click the answer that best represents your level of agreement. - Sometimes I feel that I am being pushed around in life.	1 = Strongly disagree<br>2 = Somewhat disagree<br>3 = Disagree a little<br>4 = Don't know<br>5 = Agree a little<br>6 = Somewhat agree<br>7 = Strongly agree<br>8 = Not applicable	Recode 8 = Missing<br><br>Pre: M=3.3125 SE=0.22892 SD=2.04750 Min=1 Max=7 N=80<br>Post: M=3.4028 SE=0.22696 SD=1.92580 Min=1 Max=7 N=72<br>Follow: M=3.8143 SE=0.25194 SD=2.10791 Min=1 Max=7 N=70
**Sense_of_Control_12**<br>**Q34_12**	For each statement below please click the	1 = Strongly disagree<br>2 = Somewhat disagree	Recode 8 = Missing

**Q33_12**	answer that best represents your level of agreement. - What happens to me in the future mostly depends on me.	3 = Disagree a little<br>4 = Don't know<br>5 = Agree a little<br>6 = Somewhat agree<br>7 = Strongly agree<br>8 = Not applicable	Pre: M=5.7500 SE=0.17201 SD=1.53853 Min=1 Max=7 N=80<br>Post: M=5.7222 SE=0.17382 SD=1.47488 Min=1 Max=7 N=72<br>Follow: M=5.6000 SE=0.17195 SD=1.43860 Min=1 Max=7 N=70

**Composite Sense of Control**			
Variable Names:<br>• **Pretest**<br>• **Posttest**<br>• **Follow-up**	Variable Label	Values	Recode?
**Per_mas_pre**<br>**Per_mas_post**<br>**Per_mas_follow**	Personal Mastery	Sense_of_Control_3 +<br>Sense_of_Control_6 +<br>Sense_of_Control_8 +<br>Sense_of_Control_12<br><br>Pretest<br>M = 5.6938<br>Max = 7<br>Min = 1.50<br>SD = 1.22795<br>SE = 0.13729<br>N = 80<br><br>Post-test<br>M = 5.6285<br>Max = 7<br>Min = 3.25<br>SD = 104729<br>SE = 0.12342<br>N = 72<br><br>Follow-up<br>M = 5.5845<br>Max = 7<br>Min = 1.75<br>SD = 1.16022<br>SE = 0.13867<br>N = 70	No
**Per_con_pre**<br>**Per_con_post**<br>**Per_con_follow**	Perceived Constraints	Sense_of_Control_1 +<br>Sense_of_Control_2 +<br>Sense_of_Control_4 +<br>Sense_of_Control_5 +<br>Sense_of_Control_7 +	No

		Sense_of_Control_9 +<br>Sense_of_Control_10+<br>Sense_of_Control_11<br><br>Pretest<br>M = 3.2853<br>Max = 6.75<br>Min = 1<br>SD = 1.33804<br>SE = 0.14960<br>N = 80<br><br>Post-test<br>M = 3.3333<br>Max = 6.38<br>Min = 1<br>SD = 1.42506<br>SE = 0.16795<br>N = 72<br><br>Follow-up<br>M = 3.3612<br>Max = 7<br>Min = 1<br>SD = 1.47661<br>SE = 0.17649<br>N = 70	

**Word List Immediate Free Response**			
Variable Names:<br>• **Pretest**<br>• **Posttest**<br>• **Follow-up**	Variable Label	Values	Recode?
WLI_Free_Response<br>Q152<br>Q153	Now please enter as many words as you can remember.<br><br>Write all of the words on the same line below with a space or comma between the words. You can write them in any order you wish.	Manual Entry	Yes – Will need to be manually reviewed for correctness

**Composite Free Response Word List Immediate**			
Variable Names:<br>• **Pretest**<br>• **Posttest**<br>• **Follow-up**	Variable Label	Values	Recode?
WL_Tot_Uni_pre<br>WL_Tot_Uni_post<br>WL_Tot_Uni_follow	Total number unique responses	Will need to be manually reviewed	No

		Pretest<br>M = 6.7500<br>Max = 15<br>Min = 0<br>SD = 3.01683<br>SE = 0.33729<br>N = 80<br><br>Post-test<br>M = 6.7639<br>Max = 15<br>Min = 0<br>SD = 2.90940<br>SE = 0.34288<br>N = 72<br><br>Follow-up<br>M = 7.4143<br>Max = 14<br>Min = 1<br>SD = 2.72664<br>SE = 0.32590<br>N = 70	
WL_Tot_Rep_pre<br>WL_Tot_Rep_post<br>WL_Tot_Rep_follow	Total repetitions	Will need to be manually reviewed<br><br>Pretest<br>M = 0<br>Max = 0<br>Min = 0<br>SD = 0<br>SE = 0<br>N = 80<br><br>Post-test<br>M = 0.0417<br>Max = 1<br>Min = 0<br>SD = 0.20123<br>SE = 0.02372<br>N = 72<br><br>Follow-up<br>M = 0.0286<br>Max = 1<br>Min = 0<br>SD = 0.16780<br>SE = 0.02006<br>N = 70	No
WL_Tot_Int_pre<br>WL_Tot_Int_post<br>WL_Tot_Int_follow	Total intrusions	Will need to be manually reviewed<br><br>Pretest<br>M = 0.4250	No

		Max = 7<br>Min = 0<br>SD = 0.96489<br>SE = 0.10788<br>N = 80<br><br>Post-test<br>M = 0.3056<br>Max = 4<br>Min = 0<br>SD = 0.70489<br>SE = 0.08307<br>N = 72<br><br>Follow-up<br>M = 0.2714<br>Max = 3<br>Min = 0<br>SD = 0.61199<br>SE = 0.07315<br>N = 70	

**Word List Immediate Recognition**			
Variable Names:<br>• **Pretest**<br>• **Posttest**<br>• **Follow-up**	Variable Label	Values	Recode?
WL_Recog_1<br>Q386<br>Q385	Door	1 = Yes<br>2 = No	Yes
WL_Recog_2<br>Q387<br>Q386	Garden	1 = Yes<br>2 = No	Yes
WL_Recog_3<br>Q388<br>Q387	Gabin	1 = Yes<br>2 = No	Yes
WL_Recog_4<br>Q389<br>Q388	Coin	1 = Yes<br>2 = No	Yes
WL_Recog_5<br>Q390<br>Q389	Pipe	1 = Yes<br>2 = No	Yes
WL_Recog_6<br>Q391<br>Q390	Baby	1 = Yes<br>2 = No	Yes
WL_Recog_7<br>Q392<br>Q391	Palace	1 = Yes<br>2 = No	Yes
WL_Recog_8<br>Q393<br>Q392	Friend	1 = Yes<br>2 = No	Yes
WL_Recog_9<br>Q394	Color	1 = Yes<br>2 = No	Yes

Q393			
WL_Recog_10<br>Q395<br>Q394	Bar	1 = Yes<br>2 = No	Yes
WL_Recog_11<br>Q396<br>Q395	Lake	1 = Yes<br>2 = No	Yes
WL_Recog_12<br>Q397<br>Q396	Curtain	1 = Yes<br>2 = No	Yes
WL_Recog_13<br>Q398<br>Q397	Lip	1 = Yes<br>2 = No	Yes
WL_Recog_14<br>Q399<br>Q398	Bell	24 = Yes<br>25 = No	Yes
WL_Recog_15<br>Q400<br>Q399	Dress	1 = Yes<br>2 = No	Yes
WL_Recog_16<br>Q401<br>Q400	Window	1 = Yes<br>2 = No	Yes
WL_Recog_17<br>Q402<br>Q401	Village	1 = Yes<br>2 = No	Yes
WL_Recog_18<br>Q403<br>Q402	Moon	1 = Yes<br>2 = No	Yes
WL_Recog_19<br>Q404<br>Q403	Steam	1 = Yes<br>2 = No	Yes
WL_Recog_20<br>Q405<br>Q404	River	1 = Yes<br>2 = No	Yes
WL_Recog_21<br>Q406<br>Q405	Farmer	1 = Yes<br>2 = No	Yes
WL_Recog_22<br>Q407<br>Q406	Star	1 = Yes<br>2 = No	Yes
WL_Recog_23<br>Q408<br>Q407	Ocean	1 = Yes<br>2 = No	Yes
WL_Recog_24<br>Q409<br>Q408	Clock	1 = Yes<br>2 = No	Yes
WL_Recog_25<br>Q410<br>Q409	Parent	1 = Yes<br>2 = No	Yes
WL_Recog_26<br>Q411<br>Q410	Storm	1 = Yes<br>2 = No	Yes
WL_Recog_27<br>Q412<br>Q411	Street	1 = Yes<br>2 = No	Yes

WL_Recog_28<br>Q413<br>Q412	Nose	1 = Yes<br>2 = No	Yes
WL_Recog_29<br>Q414<br>Q413	Pupil	1 = Yes<br>2 = No	Yes
WL_Recog_30<br>Q415<br>Q414	School	1 = Yes<br>2 = No	Yes
WL_Recog_31<br>Q447<br>Q476	Rock	1 = Yes<br>2 = No	Yes
WL_Recog_32<br>Q448<br>Q477	House	1 = Yes<br>2 = No	Yes
WL_Recog_33<br>Q449<br>Q478	Grass	1 = Yes<br>2 = No	Yes
WL_Recog_34<br>Q450<br>Q479	Wife	1 = Yes<br>2 = No	Yes
WL_Recog_35<br>Q451<br>Q480	City	1 = Yes<br>2 = No	Yes
WL_Recog_36<br>Q452<br>Q481	Hat	1 = Yes<br>2 = No	Yes
WL_Recog_37<br>Q453<br>Q482	Fire	1 = Yes<br>2 = No	Yes
WL_Recog_38<br>Q454<br>Q483	Skin	1 = Yes<br>2 = No	Yes
WL_Recog_39<br>Q455<br>Q484	Building	1 = Yes<br>2 = No	Yes
WL_Recog_40<br>Q456<br>Q485	Coffee	1 = Yes<br>2 = No	Yes
WL_Recog_41<br>Q457<br>Q486	Army	1 = Yes<br>2 = No	Yes
WL_Recog_42<br>Q458<br>Q487	Cell	1 = Yes<br>2 = No	Yes
WL_Recog_43<br>Q459<br>Q488	Turkey	1 = Yes<br>2 = No	Yes
WL_Recog_44<br>Q460<br>Q489	Drum	1 = Yes<br>2 = No	Yes
WL_Recog_45<br>Q460<br>Q490	Arm	1 = Yes<br>2 = No	Yes

Composite Word List Immediate Recognition			
Variable Names:<br>• **Pretest**<br>• **Posttest**<br>• **Follow-up**	Variable Label	Values	Recode?
WL_Tot_Recog_pre<br>WL_Tot_Recog_post<br>WL_Tot_Recog_follow	Total correct responses on Word List Immediate Recognition Test	Range: 0 – 45<br><br>Pretest<br>M = 37.3375<br>Max = 45<br>Min = 0<br>SD = 8.12239<br>SE = 0.90811<br>N = 80<br><br>Post-test<br>M = 38.7917<br>Max = 45<br>Min = 26<br>SD = 4.62674<br>SE = 0.54527<br>N = 72<br><br>Follow-up<br>M = 38.0857<br>Max = 45<br>Min = 21<br>SD = 5.24955<br>SE = 0.62744<br>N = 70	No

Digits Backwards			
Variable Names:<br>• **Pretest**<br>• **Posttest**<br>• **Follow-up**	Variable Label	Values	Recode?
DBL1.1Answer<br>Q161<br>Q169	Answer: (after you submit the next trial will immediately begin)	Manual Entry	Yes
DBL1.2_Answer<br>Q166<br>Q168	Answer: (after you submit the next trial will immediately begin)	Manual Entry	Yes
DBL2.1_Answer<br>Q285<br>Q176	Answer: (after you submit the next trial will immediately begin)	Manual Entry	Yes
DBL2.2_Answer<br>Q181<br>Q177	Answer: (after you submit the next trial will immediately begin)	Manual Entry	Yes
DBL_3.1_Answer<br>Q190<br>Q185	Answer: (after you submit the next trial will immediately begin)	Manual Entry	Yes

DBL_3.2_Answer<br>Q199<br>Q184	Answer: (after you submit the next trial will immediately begin)	Manual Entry	Yes
DBL_4.1_Answer<br>Q210<br>Q203	Answer: (after you submit the next trial will immediately begin)	Manual Entry	Yes
DBL_4.2_Answer<br>Q211<br>Q202	Answer: (after you submit the next trial will immediately begin)	Manual Entry	Yes
DBL_5.1_Answer<br>Q212<br>Q237	Answer: (after you submit the next trial will immediately begin)	Manual Entry	Yes
DBL_5.2_Answer<br>Q213<br>Q250	Answer: (after you submit the next trial will immediately begin)	Manual Entry	Yes
DBL_6.1_Answer<br>Q214<br>Q311	Answer: (after you submit the next trial will immediately begin)	Manual Entry	Yes
DBL6.2_Answer<br>Q215<br>Q312	Answer: (after you submit the next trial will immediately begin)	Manual Entry	Yes
DBL7.1_Answer<br>Q216<br>Q313	Answer: (after you submit the next trial will immediately begin)	Manual Entry	Yes
DBL7.2_Answer<br>Q217<br>Q314	Answer:	Manual Entry	Yes

**Composite Digits Backwards**			
DBL_Comp_pre<br>DBL_Comp_post<br>DBL_Comp_follow	Highest number of digits reached	Range: 0, 2 – 8<br>Manually Reviewed<br><br>Pretest<br>M = 4.9125<br>Max = 8<br>Min = 0<br>SD = 2.82033<br>SE = 0.31532<br>N = 80<br><br>Post-test<br>M = 4.2361<br>Max = 8<br>Min = 0<br>SD = 2.88997<br>SE = 0.34059<br>N = 72<br><br>Follow-up<br>M = 3.8143<br>Max = 8<br>Min = 0<br>SD = 3.07535<br>SE = 0.36757<br>N = 70	No

Red Green Test			
Variable Name<br>• **Pretest**<br>• **Post-test**<br>• **Follow-Up**	Variable Label	Values	Recode?
nb_1_time	None	Reaction Time (Latency)	Yes<br>Recoded such that value is listed as missing if trial was completed incorrectly, only correct trials listed
nb_1_correct	None	1 = Correct<br>0 = Incorrect	No
nb_2_time	None	Reaction Time (Latency)	Yes<br>Recoded such that value is listed as missing if trial was completed incorrectly, only correct trials listed
nb_2_correct	None	1 = Correct<br>0 = Incorrect	No
nb_3_time	None	Reaction Time (Latency)	Yes<br>Recoded such that value is listed as missing if trial was completed incorrectly, only correct trials listed
nb_3_correct	None	1 = Correct<br>0 = Incorrect	No
nb_4_time	None	Reaction Time (Latency)	Yes<br>Recoded such that value is listed as missing if trial was completed incorrectly, only correct trials listed
nb_4_correct	None	1 = Correct	No

		0 = Incorrect	
nb_5_time	None	Reaction Time (Latency)	Yes Recoded such that value is listed as missing if trial was completed incorrectly, only correct trials listed
nb_5_correct	None	1 = Correct 0 = Incorrect	No
nb_6_time	None	Reaction Time (Latency)	Yes Recoded such that value is listed as missing if trial was completed incorrectly, only correct trials listed
nb_6_correct	None	1 = Correct 0 = Incorrect	No
nb_7_time	None	Reaction Time (Latency)	Yes Recoded such that value is listed as missing if trial was completed incorrectly, only correct trials listed
nb_7_correct	None	1 = Correct 0 = Incorrect	No
nb_8_time	None	Reaction Time (Latency)	Yes Recoded such that value is listed as missing if trial was completed incorrectly, only correct trials listed
nb_8_correct	None	1 = Correct 0 = Incorrect	No
nb_9_time	None	Reaction Time (Latency)	Yes Recoded such that value is listed as missing if trial was completed incorrectly, only correct trials listed
nb_9_correct	None	1 = Correct 0 = Incorrect	No
nb_10_time	None	Reaction Time (Latency)	Yes Recoded such that value is listed as missing if trial was completed incorrectly, only correct trials listed
nb_10_correct	None	1 = Correct 0 = Incorrect	No
rb_1_time	None	Reaction Time (Latency)	Yes Recoded such that value is listed as missing if trial was completed incorrectly, only correct trials listed

rb_1_correct	None	1 = Correct<br>0 = Incorrect	No
rb_2_time	None	Reaction Time (Latency)	Yes<br>Recoded such that value is listed as missing if trial was completed incorrectly, only correct trials listed
rb_2_correct	None	1 = Correct<br>0 = Incorrect	No
rb_3_tine	None	Reaction Time (Latency)	Yes<br>Recoded such that value is listed as missing if trial was completed incorrectly, only correct trials listed
rb_3_correct	None	1 = Correct<br>0 = Incorrect	No
rb_4_time	None	Reaction Time (Latency)	Yes<br>Recoded such that value is listed as missing if trial was completed incorrectly, only correct trials listed
rb_4_correct	None	1 = Correct<br>0 = Incorrect	No
rb_5_time	None	Reaction Time (Latency)	Yes<br>Recoded such that value is listed as missing if trial was completed incorrectly, only correct trials listed
rb_5_correct	None	1 = Correct<br>0 = Incorrect	No
rb_6_time	None	Reaction Time (Latency)	Yes<br>Recoded such that value is listed as missing if trial was completed incorrectly, only correct trials listed
rb_6_correct	None	1 = Correct<br>0 = Incorrect	No
rb_7_time	None	Reaction Time (Latency)	Yes<br>Recoded such that value is listed as missing if trial was completed incorrectly, only correct trials listed
rb_7_correct	None	1 = Correct<br>0 = Incorrect	No
rb_8_time	None	Reaction Time (Latency)	Yes<br>Recoded such that value is listed as missing if trial was

			completed incorrectly, only correct trials listed
rb_8_correct	None	1 = Correct<br>0 = Incorrect	No
rb_9_time	None	Reaction Time (Latency)	Yes<br>Recoded such that value is listed as missing if trial was completed incorrectly, only correct trials listed
rb_9_correct	None	1 = Correct<br>0 = Incorrect	No
rb_10_time	None	Reaction Time (Latency)	Yes<br>Recoded such that value is listed as missing if trial was completed incorrectly, only correct trials listed
rb_10_correct	None	1 = Correct<br>0 = Incorrect	No
nsw_5_time	None	Reaction Time (Latency)	Yes<br>Recoded such that value is listed as missing if trial was completed incorrectly, only correct trials listed
nsw_5_correct	None	1 = Correct<br>0 = Incorrect	No
nsw_6_time	None	Reaction Time (Latency)	Yes<br>Recoded such that value is listed as missing if trial was completed incorrectly, only correct trials listed
nsw_6_correct	None	1 = Correct<br>0 = Incorrect	No
nsw_7_time	None	Reaction Time (Latency)	Yes<br>Recoded such that value is listed as missing if trial was completed incorrectly, only correct trials listed
nsw_7_correct	None	1 = Correct<br>0 = Incorrect	No
nsw_8_time	None	Reaction Time (Latency)	Yes<br>Recoded such that value is listed as missing if trial was completed incorrectly, only correct trials listed
nsw_8_correct	None	1 = Correct<br>0 = Incorrect	No
nsw_10_time	None	Reaction Time (Latency)	Yes<br>Recoded such that value is listed as

			missing if trial was completed incorrectly, only correct trials listed
nsw_10_correct	None	1 = Correct<br>0 = Incorrect	No
nsw_11_time	None	Reaction Time (Latency)	Yes<br>Recoded such that value is listed as missing if trial was completed incorrectly, only correct trials listed
nsw_11_correct	None	1 = Correct<br>0 = Incorrect	No
nsw_12_time	None	Reaction Time (Latency)	Yes<br>Recoded such that value is listed as missing if trial was completed incorrectly, only correct trials listed
nsw_12_correct	None	1 = Correct<br>0 = Incorrect	No
nsw_13_time	None	Reaction Time (Latency)	Yes<br>Recoded such that value is listed as missing if trial was completed incorrectly, only correct trials listed
nsw_13_correct	None	1 = Correct<br>0 = Incorrect	No
nsw_14_time	None	Reaction Time (Latency)	Yes<br>Recoded such that value is listed as missing if trial was completed incorrectly, only correct trials listed
nsw_14_correct	None	1 = Correct<br>0 = Incorrect	No
nsw_16_time	None	Reaction Time (Latency)	Yes<br>Recoded such that value is listed as missing if trial was completed incorrectly, only correct trials listed
nsw_16_correct	None	1 = Correct<br>0 = Incorrect	No
nsw_17_time	None	Reaction Time (Latency)	Yes<br>Recoded such that value is listed as missing if trial was completed incorrectly, only correct trials listed
nsw_17_correct	None	1 = Correct<br>0 = Incorrect	No
nsw_18_time	None	Reaction Time (Latency)	Yes

			Recoded such that value is listed as missing if trial was completed incorrectly, only correct trials listed
nsw_18_correct	None	1 = Correct<br>0 = Incorrect	No
nsw_20_time	None	Reaction Time (Latency)	Yes<br>Recoded such that value is listed as missing if trial was completed incorrectly, only correct trials listed
nsw_20_correct	None	1 = Correct<br>0 = Incorrect	No
nsw_21_time	None	Reaction Time (Latency)	Yes<br>Recoded such that value is listed as missing if trial was completed incorrectly, only correct trials listed
nsw_21_correct	None	1 = Correct<br>0 = Incorrect	No
nsw_22_time	None	Reaction Time (Latency)	Yes<br>Recoded such that value is listed as missing if trial was completed incorrectly, only correct trials listed
nsw_22_correct	None	1 = Correct<br>0 = Incorrect	No
nsw_23_time	None	Reaction Time (Latency)	Yes<br>Recoded such that value is listed as missing if trial was completed incorrectly, only correct trials listed
nsw_23_correct	None	1 = Correct<br>0 = Incorrect	No
nsw_25_time	None	Reaction Time (Latency)	Yes<br>Recoded such that value is listed as missing if trial was completed incorrectly, only correct trials listed
nsw_25_correct	None	1 = Correct<br>0 = Incorrect	No
nsw_26_time	None	Reaction Time (Latency)	Yes<br>Recoded such that value is listed as missing if trial was completed incorrectly, only correct trials listed
nsw_26_correct	None	1 = Correct<br>0 = Incorrect	No

nsw_27_time	None	Reaction Time (Latency)	Yes<br>Recoded such that value is listed as missing if trial was completed incorrectly, only correct trials listed
nsw_27_correct	None	1 = Correct<br>0 = Incorrect	No
nsw_28_time	None	Reaction Time (Latency)	Yes<br>Recoded such that value is listed as missing if trial was completed incorrectly, only correct trials listed
nsw_28_correct	None	1 = Correct<br>0 = Incorrect	No
nsw_30_time	None	Reaction Time (Latency)	Yes<br>Recoded such that value is listed as missing if trial was completed incorrectly, only correct trials listed
nsw_30_correct	None	1 = Correct<br>0 = Incorrect	No
nsw_31_time	None	Reaction Time (Latency)	Yes<br>Recoded such that value is listed as missing if trial was completed incorrectly, only correct trials listed
nsw_31_correct	None	1 = Correct<br>0 = Incorrect	No
nsw_32_time	None	Reaction Time (Latency)	Yes<br>Recoded such that value is listed as missing if trial was completed incorrectly, only correct trials listed
nsw_32_correct	None	1 = Correct<br>0 = Incorrect	No
sw_4_time	None	Reaction Time (Latency)	Yes<br>Recoded such that value is listed as missing if trial was completed incorrectly, only correct trials listed
sw_4_correct	None	1 = Correct<br>0 = Incorrect	No
sw_9_time	None	Reaction Time (Latency)	Yes<br>Recoded such that value is listed as missing if trial was completed incorrectly, only correct trials listed
sw_9_correct	None	1 = Correct	No

		0 = Incorrect	
sw_15_time	None	Reaction Time (Latency)	Yes<br>Recoded such that value is listed as missing if trial was completed incorrectly, only correct trials listed
sw_15_correct	None	1 = Correct<br>0 = Incorrect	No
sw_19_time	None	Reaction Time (Latency)	Yes<br>Recoded such that value is listed as missing if trial was completed incorrectly, only correct trials listed
sw_19_correct	None	1 = Correct<br>0 = Incorrect	No
sw_24_time	None	Reaction Time (Latency)	Yes<br>Recoded such that value is listed as missing if trial was completed incorrectly, only correct trials listed
sw_24_correct	None	1 = Correct<br>0 = Incorrect	No
sw_29_time	None	Reaction Time (Latency)	Yes<br>Recoded such that value is listed as missing if trial was completed incorrectly, only correct trials listed
sw_29_correct	None	1 = Correct<br>0 = Incorrect	No

**Red Green Test – Composite Scores**			
Variable Name<br>• **Pretest**<br>• **Post-test**<br>• **Follow-Up**	Variable Label	Values	Recode?
nb_AVERAGE_time_pre<br>nb_AVERAGE_time_post<br>nb_AVERAGE_time_follow	None	Pre-test:<br>M = .9773<br>SE = .04206<br>SD = .36427<br>Min = .50<br>Max = 2.81<br>N = 75<br><br>Post-test:<br>M = 1.0988<br>SE = .06452<br>SD = .54745<br>Min = .59<br>Max = 4.53	No

		N =72<br><br>Follow-up:<br>M = 1.0229<br>SE = .06024<br>SD = .50760<br>Min = .53<br>Max = 3.88<br>N = 71	
nb_MEDIAN_time_pre<br>nb_MEDIAN_time_post<br>nb_MEDIAN_time_follow	None	Pre-test:<br>M = .8916<br>SE = .03598<br>SD = .31161<br>Min = .48<br>Max = 2.8`<br>N = 75<br><br>Post-test:<br>M = .9974<br>SE = .06041<br>SD = .51257<br>Min = .48<br>Max = 4.26<br>N = 72<br><br>Follow-up:<br>M = .9313<br>SE = .05312<br>SD = .44763<br>Min = .53<br>Max = 3.65<br>N = 71	No
nb_COUNT_correct_pre<br>nb_COUNT_correct_post<br>nb_COUNT_correct_follow	Baseline Non-reverse Number Correct	Pre-test:<br>M = 9.4026<br>SE = .21018<br>SD = 1.84434<br>Min = 0<br>Max = 10<br>N = 77<br><br>Post-test:<br>M = 9.4189<br>SE = .20455<br>SD = 1.75957<br>Min = 0<br>Max = 10<br>N = 74<br><br>Follow-up:<br>M = 9.6901<br>SE = .10537<br>SD = .88788<br>Min = 5<br>Max = 10	No

		N = 71	
rb_AVERAGE_time_pre<br>rb_AVERAGE_time_post<br>rb_AVERAGE_time_follow	None	Pre-test:<br>M = 1.1952<br>SE = .06553<br>SD = .54432<br>Min = .31<br>Max = 3.42<br>N = 69<br><br>Post-test:<br>M = 1.1921<br>SE = .05349<br>SD = .44111<br>Min = .54<br>Max = 2.66<br>N = 68<br><br>Follow-up:<br>M = 1.1515<br>SE = .05522<br>SD = .44862<br>Min = .43<br>Max = 3.26<br>N = 66	No
rb_MEDIAN_time_pre<br>rb_MEDIAN_time_post<br>rb_MEDIAN_time_follow	None	Pre-test:<br>M = 1.0655<br>SE = .06054<br>SD = .50286<br>Min = .18<br>Max = 3.42<br>N = 69<br><br>Post-test:<br>M = 1.0542<br>SE = .04730<br>SD = .39004<br>Min = .54<br>Max = 2.66<br>N = 68<br><br>Follow-up:<br>M = 1.0387<br>SE = .04992<br>SD = .40552<br>Min = .43<br>Max = 3.26<br>N = 66	No
rb_COUNT_correct_pre<br>rb_COUNT_correct_post<br>rb_COUNT_correct_follow	Baseline Reverse Number Correct	Pre-test:<br>M = 8.0390<br>SE = .40437<br>SD = 3.54832<br>Min = 0<br>Max = 10	No

		N = 77<br><br>Post-test:<br>M = 8.3649<br>SE = .36385<br>SD = 3.12995<br>Min = 0<br>Max = 10<br>N = 74<br><br>Follow-up:<br>M = 8.1831<br>SE = .38131<br>SD = 3.21296<br>Min = 0<br>Max = 10<br>N = 71	
nsw_AVERAGE_time_pre<br>nsw_AVERAGE_time_post<br>nsw_AVERAGE_time_follow	None	Pre-test:<br>M = .9495<br>SE = .03094<br>SD = .26796<br>Min = .35<br>Max = 1.83<br>N = 75<br><br>Post-test:<br>M = 1.0663<br>SE = .06453<br>SD = .54756<br>Min = .49<br>Max = 3.58<br>N = 72<br><br>Follow-up:<br>M = 1.0278<br>SE = .05398<br>SD = .453=481<br>Min = .56<br>Max = 2.84<br>N = 71	No
nsw_MEDIAN_time_pre<br>nsw_MEDIAN_time_post<br>nsw_MEDIAN_time_follow	None	Pre-test:<br>M = .85-1<br>SE = .02428<br>SD = .21030<br>Min = .35<br>Max = 1.45<br>N = 75<br><br>Post-test:<br>M = .8922<br>SE = .03657<br>SD = .31032<br>Min = .46<br>Max = 2.24	No

		N = 72<br><br>Follow-up:<br>M = .8598<br>SE = .02724<br>SD = .22957<br>Min = .52<br>Max = 1.85<br>N = 71	
nsw_COUNT_correct_pre<br>nsw_COUNT_correct_post<br>nsw_COUNT_correct_follow	Mixed Non-revers Number Correct	Pre-test:<br>M = 20.8052<br>SE = .50798<br>SD = 4.45752<br>Min = 0<br>Max = 23<br>N = 77<br><br>Post-test:<br>M = 19.5135<br>SE = .57604<br>SD = 4.95526<br>Min = 0<br>Max = 23<br>N = 74<br><br>Follow-up:<br>M = 20.7042<br>SE = .42383<br>SD = 3.57129<br>Min = 9<br>Max = 23<br>N = 71	No
sw_AVERAGE_time_pre<br>sw_AVERAGE_time_post<br>sw_AVERAGE_time_follow	None	Pre-test:<br>M = 1.4553<br>SE = .07573<br>SD = .65584<br>Min = .55<br>Max = 4.88<br>N = 75<br><br>Post-test:<br>M = 1.5365<br>SE = .07051<br>SD = .59827<br>Min = .43<br>Max = 4<br>N = 72<br><br>Follow-up:<br>M = 1.3871<br>SE = .05856<br>SD = .49346<br>Min = .70<br>Max = 4.08	No

		N = 71	
sw_MEDIAN_time_pre<br>sw_MEDIAN_time_post<br>sw_MEDIAN_time_follow	None	Pre-test:<br>M = 1.2808<br>SE = .04909<br>SD = .42510<br>Min = .47<br>Max = 2.51<br>N = 75<br><br>Post-test:<br>M = 1.3854<br>SE = .06025<br>SD = .51127<br>Min = .42<br>Max = 3.37<br>N = 72<br><br>Follow-up:<br>M = 1.2380<br>SE = .03537<br>SD = .29801<br>Min = .70<br>Max = 1.95<br>N = 71	No
sw_COUNT_correct_pre<br>sw_COUNT_correct_post<br>sw_COUNT_correct_follow	Mixed Reverse Number Correct	Pre-test:<br>M = 5.2987<br>SE = .14912<br>SD = 1.30852<br>Min = 0<br>Max = 6<br>N = 77<br><br>Post-test:<br>M = 5.0811<br>SE = .15721<br>SD = 1.35239<br>Min = 0<br>Max = 6<br>N = 74<br><br>Follow-up:<br>M = 5.2676<br>SE = .11516<br>SD = .97039<br>Min = 2<br>Max = 6<br>N = 71	No
Mxf_AVERAGE_time_pre<br>Mxf_AVERAGE_time_post<br>Mxf_AVERAGE_time_follow	None	Pre-test:<br>M = 1.0516<br>SE = .03508<br>SD = .30384<br>Min = .38<br>Max = 2.11	No

		N = 75<br><br>Post-test:<br>M = 1.1655<br>SE = .06220<br>SD = .52782<br>Min = .48<br>Max = 3.67<br>N = 72<br><br>Follow-up:<br>M = 1.1005<br>SE = .04800<br>SD = .40447<br>Min = .59<br>Max = 2.72<br>N = 71	
mxf_MEDIAN_time_pre<br>mxf_MEDIAN_time_post<br>mxf_MEDIAN_time_follow	None	Pre-test:<br>M = .8886<br>SE = .02543<br>SD = .22027<br>Min = .44<br>Max = 1.67<br>N = 75<br><br>Post-test:<br>M = .9559<br>SE = .03969<br>SD = .33680<br>Min = .44<br>Max = 2.26<br>N = 72<br><br>Follow-up:<br>M = .9055<br>SE = .02697<br>SD = .22726<br>Min = .56<br>Max = 1.72<br>N = 71	No
mxf_COUNT_correct_pre<br>mxf_COUNT_correct_post<br>mxf_COUNT_correct_follow	Mixed All Correct	Pre-test:<br>M = 26.1039<br>SE = .64878<br>SD = 5.69299<br>Min = 0<br>Max = 29<br>N = 77<br><br>Post-test:<br>M = 24.5946<br>SE = .72377<br>SD = 6.22615<br>Min = 0<br>Max = 29	No

		N = 74<br><br>Follow-up:<br>M = 25.9718<br>SE = .51730<br>SD = 4.35881<br>Min = 12<br>Max = 29<br>N = 71	

Additional Posttest Questions			
Q474	Have you been wearing your Fitbit device on your dominant or non-dominant wrist?<br><br>(The dominant side is the side you choose to perform fine and gross motor tasks such as writing, cutting, catching and throwing a ball).	1 = Dominant Side<br>2 = Non-dominant Side	No
**Q475_post**	Have you ever smoked cigarettes regularly?	1 = Yes<br>2 = No<br>3 = Not applicable	3 = Missing
**Q476_post**	Do you currently smoke cigarettes regularly?	1 = Yes<br>2 = No<br>3 = Not applicable	3= Missing

Additional Computed Variables			
Variable Name	Variable Label	Values	Recode?
EducYrs	How many years of education have you had?	Range: 12 - 26	No
Change_Selfcontrol	Change in Selfcontrol between Pretest and Follow-up	M = 0.26<br>SD = 0.51<br>Min = -0.92<br>Max = 1.69	
Condition_pre		0 = Comparison Condition<br>1 = Self-control Treatment Group	
