# Supplementary material for: Effects of a digital self-control intervention to increase physical activity in middle-aged adults
Source: J Health Psychol. 2023 Apr 12;28(10):984–96. doi: 10.1177/13591053231166756 (PMC10466994; doi:10.1177/13591053231166756)
Supplement: sj-pdf-4-hpq-10.1177_13591053231166756 – Supplemental material for Effects of a digital self-control intervention to increase physical activity in middle-aged adults [file sj-pdf-4-hpq-10.1177_13591053231166756.pdf]

Read Me File

- **Codebook_FigShare**
  - Presents and explains (incl. descriptive statistics at pretest, posttest, and follow-up) all variables available in the dataet “PrePostFollowWide_FigShare”

- **PrePostFollowWide_FigShare.sav**
  - This is the raw dataset (.sav), which can be openend in SPSS or R. This dataset includes all variables which were collected during the pretest, posttest, and follow-up assessemnts. Additonally, it also includes recoded and calculated variables at the end of the dataset

- **PrePostFollowWide_FigShare.sps**
  - This syntax file (.sps) includes all syntax which was used to recode and calculate the variables used for the analyses (incl. reliability analyses)

- **PrePostFollowLong_FigShare.sav**
  - This is the dataset in a long format, which can be used for the multilevel analyses conducted in R. All relevant variables for the multilevel analyses to analyse changes from pretest to posttest to follow-up are included in this dataset.

- **PrePostFollowAnalyses_FigShare.R**
  - This is the syntax file which can be opened in R. This file includes the syntax to conduct the pre-post-follow multilevel analyses.

- **DailyData_FigShare.sav**
  - This is the dataset in a long format which included the daily activity data collected on the Fitbit devices.

- **StepAnalyses_FigShare.R**
  - This is the syntax file which can be opened in R. This file includes the syntax to conduct the multilevel analyses to examine changes in daily activity.
